# Towards Understanding the Reactivity and Optical Properties of Organosilicon Sulfide Clusters

**DOI:** 10.1002/anie.202011370

**Published:** 2020-12-15

**Authors:** Katharina Hanau, Sebastian Schwan, Moritz R. Schäfer, Marius J. Müller, Christof Dues, Niklas Rinn, Simone Sanna, Sangam Chatterjee, Doreen Mollenhauer, Stefanie Dehnen

**Affiliations:** ^1^ Fachbereich Chemie und Wissenschaftliches Zentrum für Materialwissenschaften (WZMW) Philipps-Universität Marburg Hans-Meerwein-Str. 4 35043 Marburg Germany; ^2^ Institute of Physical Chemistry Justus Liebig University Giessen Heinrich-Buff-Ring 17 35392 Giessen Germany; ^3^ Center for Materials Research (LaMa) Justus Liebig University Giessen Heinrich-Buff-Ring 16 35392 Giessen Germany; ^4^ Institute of Experimental Physics I and Center for Materials Research (LaMa) Justus Liebig University Giessen Heinrich-Buff-Ring 17 35392 Giessen Germany; ^5^ Institute of Theoretical Physics and Center for Materials Research (LaMa) Justus Liebig University Giessen Heinrich-Buff-Ring 16 35392 Giessen Germany

**Keywords:** DFT calculations, gold, nonlinear optics, organosilicon chalcogenide clusters, X-ray diffraction

## Abstract

We report the extension of the class of organotetrel sulfide clusters with further examples of the still rare silicon‐based species, synthesized from RSiCl_3_ with R=phenyl (Ph, **I**), naphthyl (Np, **II**), and styryl (Sty, **III**) with Na_2_S. Besides known [(PhSi)_4_S_6_] (**IV**), new compounds [(NpSi)_4_S_6_] (**1**) and [(StySi)_4_S_6_] (**2**) were obtained, the first two of which underwent reactions with [AuCl(PPh_3_)] to form ternary complexes. DFT studies of cluster dimers helped us understand the differences between the habit of {Si_4_S_6_}‐ and {Sn_4_S_6_}‐based compounds. Crystalline **1** showed a pronounced nonlinear optical response, while for intrinsically amorphous **2**, the chemical damage threshold seems to inhibit a corresponding observation. Calculations within the independent particle approximation served to rationalize and compare electronic and optical excitations of [(RSi)_4_S_6_] clusters (R=Ph, Np). The calculations reproduced the measured data and allowed for the interpretation of the main spectroscopic features.

## Graphical abstract

To gain insight into the nonlinear optical properties and reactivity of organotetrel chalcogenide clusters, we studied the silicon homologues, [(RSi)_4_S_6_] (R=Ph, Np, Sty), and their products upon reaction with [AuCl(PPh_3_)], [{RSi(μ‐S)}_2_{AuPPh_3_(μ‐S)}_2_]. Quantum chemical studies of cluster dimers elucidated the differences in the habit of silicon and tin compounds, and DFT calculations of crystalline models confirmed the applicability of these methods.

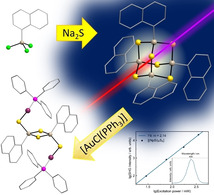

## Introduction

The chemical and structural properties of tetrel chalcogenide clusters with organic substituents have been extensively studied in the past.^[1]^ In particular, a large variety of compounds has been reported for the element combinations Sn/E[^1a^, ^2^] (E=S, Se, Te) and Ge/E,[^1c^, ^3^] whereas significantly fewer compounds with Si/E‐based cluster cores have been known so far.[^1b^, ^4^] Several studies investigating the reactivity and properties of tin chalcogenide clusters were undertaken, showing the possible derivatization of the organic substituents[^2f^, ^5^] as well as the formation of ternary inorganic cluster cores by introducing transition metal complexes.^[6]^

In addition to the chemical features of such clusters, the styryl‐substituted cluster [(StySn)_4_S_6_] (Sty=4‐vinylphenyl), which was determined via DFT calculations to have a heteroadamantane‐type molecular structure, was recently shown to possess an extreme nonlinear optical behavior.^[7]^ These findings led to the investigation of further compounds of the type [(RSn)_4_S_6_] with cyclic and/or aromatic substituents R to understand how the substituents influence the compounds’ properties. Generally, it was shown that distinct order within the solid material (crystallinity or pronounced π‐stacking interactions) leads to second‐harmonic generation (SHG), while a high degree of amorphousness results in white‐light generation (WLG).^[8]^

In this context, we intended to find out, whether the principles of Sn/E chemistry can also be applied to the Si/E elemental combination, and whether a replacement of Sn atoms with Si atoms leads to changes in the cluster's reactivity and properties. Herein, we present the first results of these studies.

The preparation of corresponding silicon analogs from RSiCl_3_ (R=organic substituent) and a corresponding chalcogen source is much more challenging than the corresponding tin or germanium chemistry, as the use of the most appropriate reactant, E(SiMe_3_)_2_ (E=S, Se, Te), logically lacks the driving force of Si−Cl bond formation. Here, a Si−Cl bond needs to be cleaved at the same time in the other reactant. So, the use of binary chalcogen salts such as A_2_E (A=alkali metal) is required, which leads to a more complicated work‐up. However, besides the reproduction of known [(PhSi)_4_S_6_] (**IV**) we herein report on the successful synthesis of two new organosilicon sulfide compounds, [(NpSi)_4_S_6_] (**1**) and [(StySi)_4_S_6_] (**2**), their characterization and follow‐up chemistry with gold complexes to form ternary complexes [{RSi(μ‐S)}_2_{AuPPh_3_(μ‐S)}_2_] (**3**: R=Ph, **4**: R=Np). We used density functional theory (DFT) methods to gain insight in structural features of the adamantane‐based compounds, in particular regarding inter‐cluster interactions that contribute to the macroscopic habitus of the solid. All new compounds were investigated regarding their linear and nonlinear optical properties, which were additionally studied by means of quantum chemistry. The calculations explain the preferred habitus of the compounds, and reveal the deep impact of the ligands in the optical response

## Results and Discussion

### Syntheses, Crystal Structures and Spectroscopic Data

**Scheme 1:**
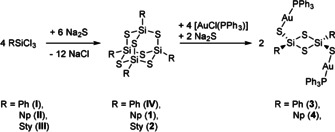
Summary of reactions done to produce compounds **IV** and **1**–**5**.

The reactions of RSiCl_3_ with R=phenyl (Ph, **I**), naphthyl (Np, **II**), and styryl (Sty, **III**) with Na_2_S discussed herein are summarized in Scheme 1.

**Figure 1:**
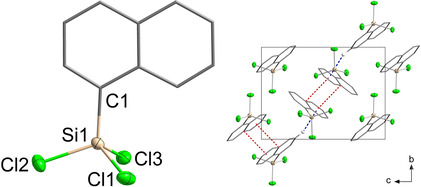
Molecular structure (left) and crystal structure (right) of **II**. Ellipsoids drawn at 50 % probability level. H atoms omitted for clarity; π‐stacking and CH/π‐interactions are indicated as dashed lines. Selected distances and angles [Å, °]: Si−Cl 2.0313(7)–2.0385(7)), Si−C 1.8426(18); Cl−Si−Cl 106.42(3)–107.58(3), Cl−Si−C 111.42(6)–112.56(6), C_Np_⋅⋅⋅π (red) 3.4397(18), C_Np_‐H⋅⋅⋅π (blue) 2.9371(1).^[15]^

While synthesizing NpSiCl_3_ (**II**) as a precursor for [(NpSi)_4_S_6_] (**1**), single crystals were obtained upon distillation, and the molecular structure (Figure 1, left) was determined via single crystal X‐ray diffraction. As expected, the Si atom exhibits a trigonal pyramidal coordination environment. Furthermore, π‐interactions between the naphthyl substituents are observed in the unit cell, alternating between parallel‐displaced π‐stacking and T‐shaped CH/π‐interactions (Figure 1, right). The distance between the adjacent naphthyl rings with π‐stacking is slightly larger (3.4397(18) Å) than the distance between the layers in graphene (3.35 Å),^[9]^ whereas the distance between the center of one of the naphthyl rings to the closest H atom of the next perpendicular naphthyl ring (2.9371(1) Å) is in the typical range of strong CH/π‐interactions.^[10]^

**Figure 2:**
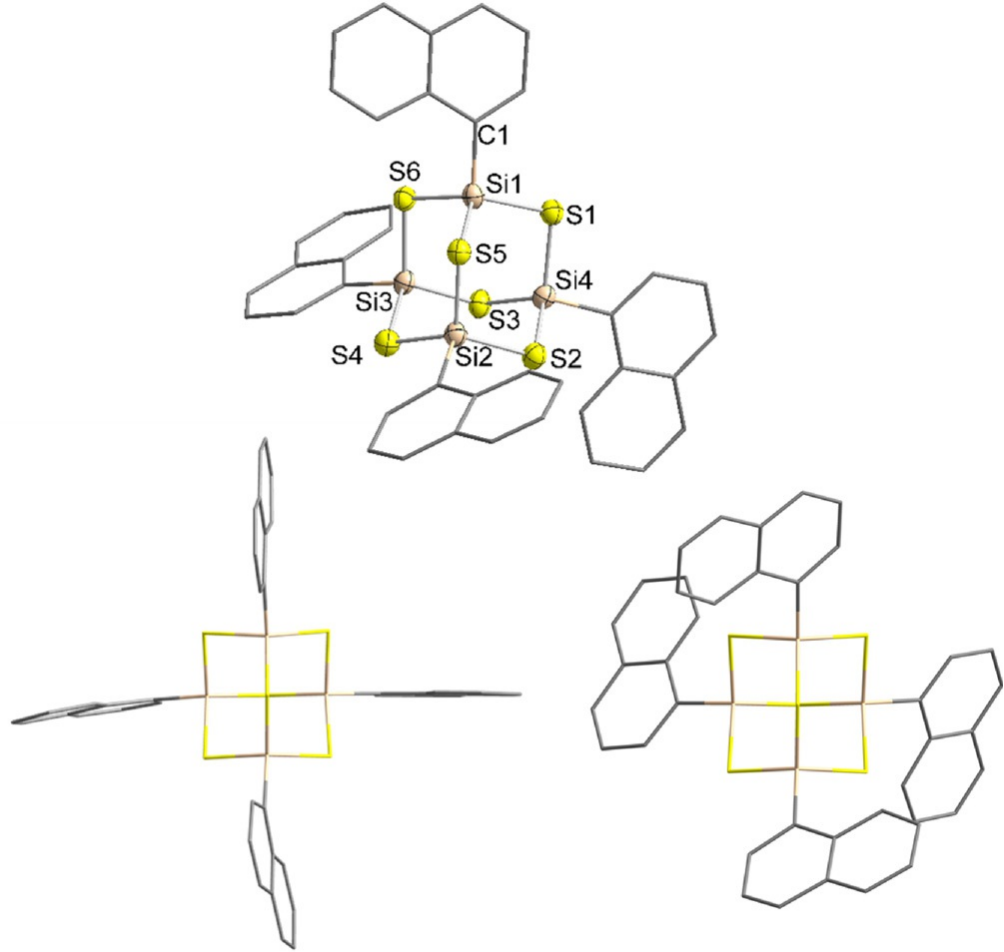
Molecular structure of **1** (top) and visualization of the two different orientations of the organic substituents in the two individuals within the asymmetric unit (bottom); the structure at the top refers to the bottom right individual. Ellipsoids drawn at 50 % probability level. H atoms omitted for clarity. Selected distances and angles [Å, °]: Si−S 2.111(3)–2.153(3), Si−C 1.837(8)–1.876(8); Si−S−Si 103.42(11)–105.64(13), S−Si−S 110.31(12)–113.28(12), S−Si−C 104.1(3)–110.1(2).^[15]^

The reaction of **II** with 1.5 equivalents of Na_2_S affords a crystalline, colorless solid. Upon dissolution in toluene and cooling to −25 °C, single crystals of [(NpSi)_4_S_6_]⋅0.5 C_7_H_8_ (**1⋅**0.5 C_7_H_8_) suitable for X‐ray diffraction were obtained. The molecular structure of **1** is shown in Figure 2. **1** crystallizes in the triclinic space group *P* $\bar{1}$ with two molecules per asymmetric unit. Like [(PhSi)_4_S_6_], **1** is an adamantane‐type Si/S cluster with four organic substituents, each bound to one Si atom. The molecule shows minor deviations from *T_d_* symmetry, due to slightly different angles in the inorganic core and different orientations of the organic substituents. Furthermore, the two individuals differ greatly in the orientation of the organic substituents (see Figure 2, bottom), indicating a very low energy barrier for rotation of the organic substituents about the Sn−C bond, which in most cases leads to intrinsic amorphousness. Notably, while both [(PhSn)_4_S_6_] and [(NpSn)_4_S_6_] are amorphous (with the Np compound comprising a considerable amount of order according to its optical response, see also below),^[7b]^ the two Si analogs are crystalline. Initially, this was ascribed to the smaller radius of the Si/S core with respect to the Sn/S core only, as the corresponding smaller volume per given number of cluster molecules causes the aromatic rings of neighboring clusters to approach more closely overall, and thus form more efficient π‐stacking interactions. In this work, we present a refined version of this interpretation (see below).

The reaction of StySiCl_3_ (**III**) with 1.5 equivalents of Na_2_S should proceed similarly and lead to the formation of [(StySi)_4_S_6_] (**2**). However, only the crude product could be isolated upon evaporation of the solvent in form of a colorless “solid” with the consistency of cotton candy or honey—depending on the duration of the reaction and drying process. We attribute this to the high polymerization tendency of the styryl substituents, and the corresponding oligomerization/polymerization that proceeds visibly as time goes by. Notably, this was not the case for the Sn analogs, which we take as a further hint for the correlation of the nature of the T/E cluster core with the properties of the organic substituents: in the Sn/S clusters, the styryl substituents of neighboring cluster units are obviously not close enough to allow for a chemical reaction of neighboring vinyl groups. As a consequence of the different situation with an underlying Si/S cluster core, **2** could neither be obtained as single crystals, nor could individual clusters be detected by means of liquid injection field desorption/ionization (LIFDI) or electrospray ionization (ESI) mass spectrometry. However, we were able to collect ^29^Si NMR data of a freshly prepared sample of **2** that turned out to be sufficiently soluble. A downfield shift of ≈9 ppm of the observed singlet with respect to the precursor signal is in accordance with the NMR data of other Si/S adamantane‐type clusters and their corresponding organosilicon trichloride precursors reported previously, such as [(EtSi)_4_S_6_]/EtSiCl_3_, [(PhSi)_4_S_6_]/PhSiCl_3_, or [(CH_2_=CHSi)_4_S_6_]/CH_2_=CHSiCl_3_.^[11]^ Another indication is given by the ^1^H and ^13^C NMR spectra, which clearly show the signals of the styryl substituent at a significant downfield‐shift compared to the starting compound **III**, with the *ipso*‐ and *meta*‐atoms being the most affected, which indicates a reaction at the Si atom to have taken place. Hence, the NMR data indicate the formation of a compound comprising individual adamantane‐type clusters [(StySi)_4_S_6_]. Yet we assume that they start to unstoppably form oligo‐/polymers shortly upon synthesis.

**Figure 3:**
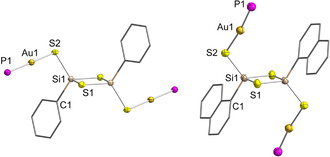
Molecular structure of **3** (left) and **4** (right) Ellipsoids drawn at 50 % probability level. H atoms and P‐bound phenyl groups omitted for clarity. Selected distances and angles [Å, °]: **3**: Si−S 2.091(2)–2.144(2), S−Au 2.3136(16), Au−P 2.2534(16), Si−C 1.870(7); Si−S−Si 82.40(9), S−Si−S 97.60(9)–115.54(10), Si−S−Au 90.97(7), S−Au−P 178.03(6). **4**: Si−S 2.095(2)–2.1589(3), S−Au 2.297(2), Au−P 2.256(2), Si−C 1.865(8), Si−S−Si 82.58(10), S−Si−S 97.42(10)–115.64(10), Si−S−Au 103.57(9), S−Au−P 173.35(7).^[15]^

Further studies on the reactivity of the adamantane‐like organosiliconsulfide clusters were undertaken. The addition of Na_2_S and [AuCl(PPh_3_)] leads to the fragmentation of the clusters and the formation of the four‐membered rings [{RSi(μ‐S)}_2_{AuPPh_3_(μ‐S)}_2_] (**3**: R=Ph, **4**: R=Np). Colorless single crystals of **3**⋅CH_2_Cl_2_ and **4** were obtained by layering the respective reaction solutions with *n*‐hexane. Their molecular structures are shown in Figure 3. **3**⋅CH_2_Cl_2_ crystallizes in the monoclinic space group *C*2/*c*, and **4** crystallizes in the triclinic space group *P* $\bar{1}$ , both with half a molecule per asymmetric unit. The molecules in these compounds have an inversion center in the center of an {RSi(μ‐S)}_2_ ring, with nearly linear {Au(PPh_3_)(μ‐S)} substituents attached to the Si atoms. Although the structural parameters of the central unit in both compounds are quite similar, they differ significantly in the orientation of the {Au(PPh_3_)(μ‐S)} units: whereas they point away from the central {Si_2_S_2_} ring in **3** with a C1−Si1−S2−Au1 *cis* arrangement, the corresponding atoms in **4** show a *trans* arrangement. Thus, while the interatomic distances in these compounds are similar, the Si1−S2−Au1 and S2−Au1−P1 bond angles are different. Homologous Sn/S compounds exhibiting this structural motif as well as a *cis* or *trans* arrangement depending on the organic moiety at the tetrel atom were described previously, showing the same trends in the T−S−Au and S−Au−P bond angles.^[12]^

Upon fragmentation of the adamantane‐type cluster and formation of **4**, a further downfield shift by 5.6 ppm was observed in the ^29^Si NMR spectrum. Table 1 summarizes the respective NMR data for compounds **I**–**IV**, **1**, **2**, and **4**. Note that NMR data of compound **3** were not obtained owing to very poor solubility of the crystals.

**Table 1:** ^29^Si NMR shifts of compounds **I**–**IV**, **1**, **2**, and **4** reported in this work.

Compound	**I**	**II**	**III**	**IV**	**1**	**2**	**4**
δ (^29^Si)/ppm	−0.9	−0.4	−0.9	10.9	6.8	8.7	12.4

### Quantum Chemical Investigation of Structural Features

It was experimentally shown, that [(PhSi)_4_S_6_] (**IV**) and [(NpSi)_4_S_6_] (**1**) are observed in a crystalline phase, while [(PhSn)_4_S_6_] and [(NpSn)_4_S_6_] are intrinsically amorphous. Nevertheless, it was suggested that [(NpSn)_4_S_6_] features a structure with a higher amount of order compared to [(PhSn)_4_S_6_], owing to more efficient π‐stacking interactions between the Np substituents as compared to the Ph substituents.^[7]^ This assumption was based on the different nonlinear optical responses of the two compounds, which showed SHG (requiring phase matching) for the Np compound but not for the Ph analog, for which WLG was reported.

To explain the general difference observed in the [(RSn)_4_S_6_] and [(RSi)_4_S_6_] cluster compounds with R=Ph, Np, one may consider the smaller radius of the {Si_4_S_6_} core with respect to the {Sn_4_S_6_} core to enable a stronger interaction of the neighboring clusters through more efficient π‐stacking interactions. To investigate this hypothesis, quantum chemical calculations of the interaction in cluster dimers as a minimal model were carried out, which already provide valuable insights into the cluster interaction that can be transferred to the extended crystalline or amorphous material.

**Figure 4:**
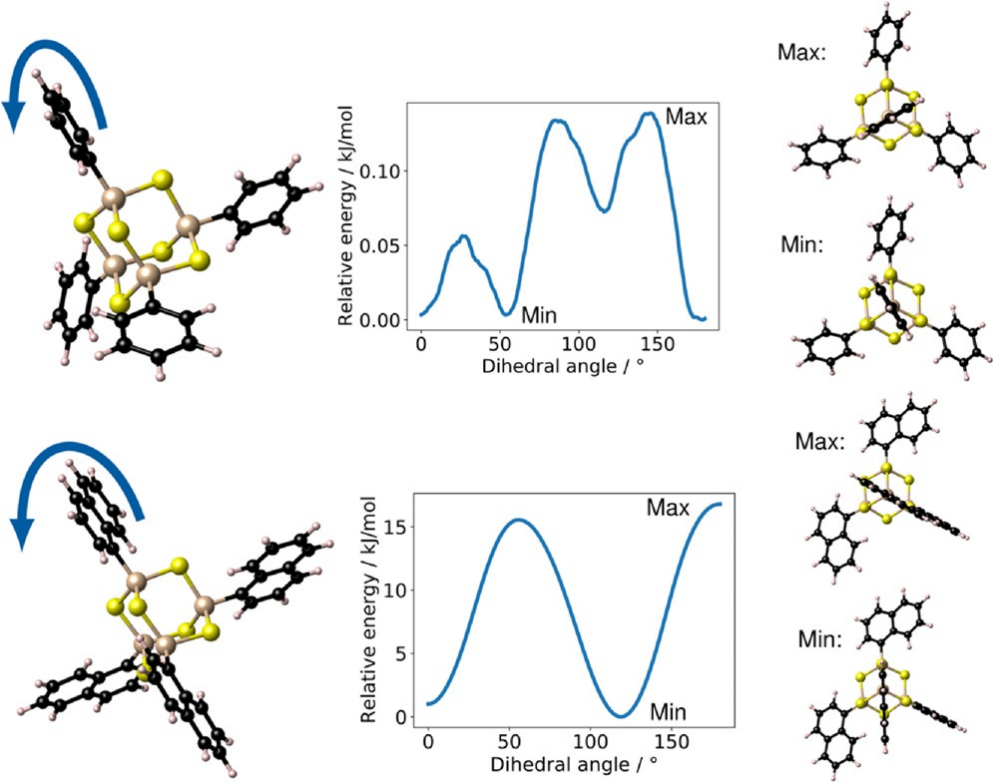
Scan of the phenyl group (top) and naphthyl group (bottom) rotations by variation of the dihedral angle for corresponding clusters [(PhSi)_4_S_6_] and [(NpSi)_4_S_6_] calculated at the BP86‐D3/cc‐pVDZ(‐PP) level of theory. Structures at the minimum and maximum positions of the scan are shown on the right hand side. The corresponding scans for the Sn/S core cluster analoges are given in Figure S12.

**Figure 5:**
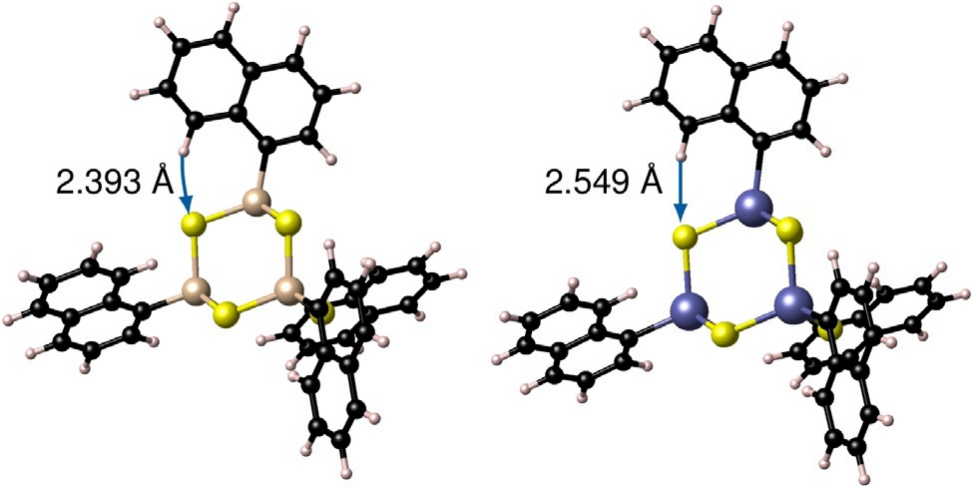
Comparison of the S−H distances at the energetic maximum of the rotational scan for [(NpSi)_4_S_6_] (left) and [(NpSn)_4_S_6_] (right), calculated at the BP86‐D3/cc‐pVDZ(‐PP) level of theory.

In order to classify the flexibility of the substituents, we initially calculated the rotation barriers of the substituents for both cluster cores. Rotation barriers of the substituent at the single cluster are hardly present (<1 kJ mol^−1^) for the clusters with phenyl substituents, while for the clusters with naphthyl substituents, a barrier height of 17 kJ mol^−1^ is obtained for the [(NpSi)_4_S_6_] cluster and 9 kJ mol^−1^ for the [(NpSn)_4_S_6_] cluster (see Figure 4 and Figure S12). The larger rotation barrier for the cluster with an {Si_4_S_6_} core as compared to the cluster with an {Sn_4_S_6_} core can indeed be explained by the smaller core radius. The smaller Si−C distance in this cluster (1.889 Å) in comparison with the corresponding Sn−C distance (2.088 Å) increases the barrier by a stronger interaction between the hydrogen atom of the substituent and the sulfur atoms of the cluster. The relative positions of S and H atoms at the rotation barrier are visualized in Figure 5.

Furthermore, the substituent–substituent interaction can increase the rotation barrier, but this is of minor importance for the considered conformers. In summary, the calculations indicate a high orientational flexibility for phenyl substituents on both Si/S and Sn/S clusters, as opposed to a comparatively lower freedom of orientation for naphthyl substituents. The latter show a higher orientational flexibility for the cluster with larger core radius (Sn/S) than for those with smaller core radius (Si/S), which is in agreement with the tendency for (a) higher order in compounds with R=Np than in compounds with R=Ph, and (b) higher order in {Si_4_S_6_}‐based clusters than in {Sn_4_S_6_}‐based clusters.

**Figure 6:**
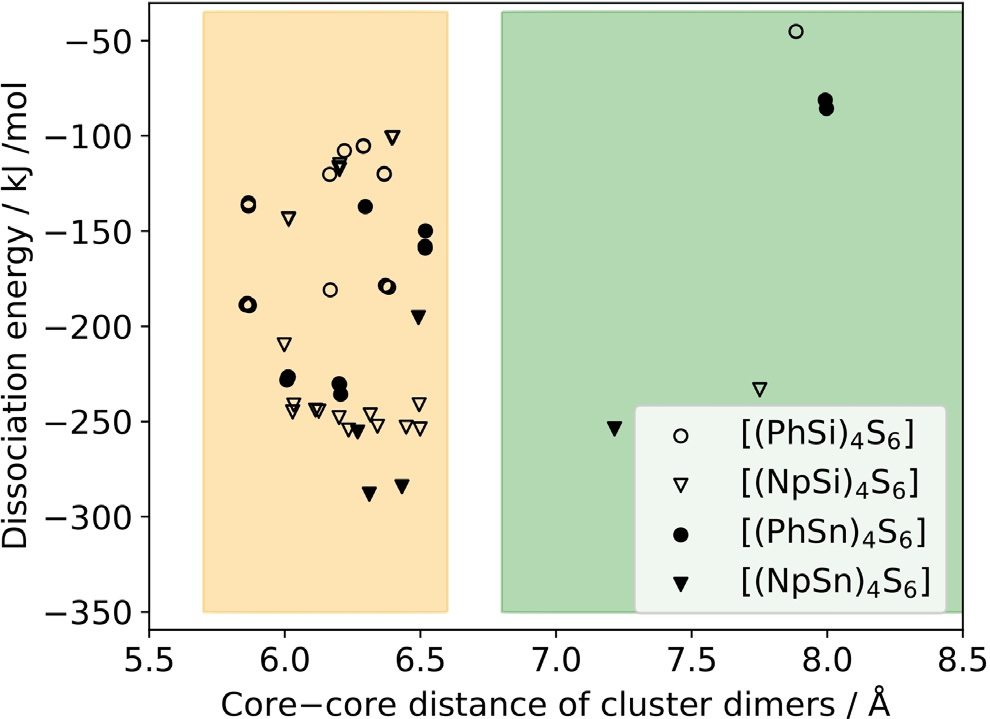
Dissociation energy of cluster dimers, plotted against the corresponding core–core distance calculated at the BP86‐D3/cc‐pVDZ(‐PP) level of theory. Medium core–core distances are indicated by an orange background, large core–core distances by a green background. The region of small core–core distances is plotted in Figure S13.

**Figure 7:**
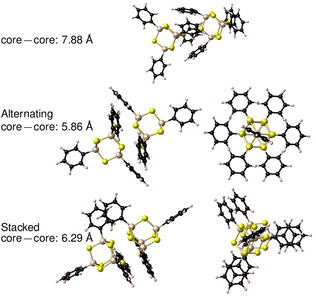
Examples for cluster dimers emerging from the calculations, shown for dimers of [(PhSi)_4_S_6_] calculated at the BP86‐D3/cc‐pVDZ(‐PP) level of theory. Top: cluster dimer with a large core–core distance. Centre: cluster dimer with medium core–core distance and alternating substituents (two views). Bottom: cluster dimer with medium core–core distance and stacked substituents (two views).

The investigation of the cluster dimer structures was performed to analyze the interaction between the molecules with different cluster cores and substituents. The cluster dimer structures were determined by two computational approaches and their results were combined to obtain a larger number of possible conformers. The dissociation energies of all cluster dimers are plotted against their core–core distance in Figure 6, and selected cluster dimer structures are plotted in Figure 7.

**Figure 8:**
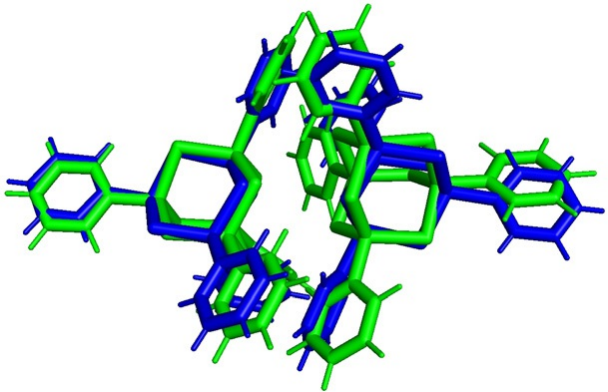
Comparison of the relative orientation of [(PhSi)_4_S_6_], as an example of the generated dimer structures (green, core–core distance 6.37 Å) calculated at the BP86‐D3/cc‐pVDZ(‐PP) level of theory, and a dimer extracted from the crystal structure of [(PhSi)_4_S_6_]^[7a]^ (blue, core–core distance 7.05 Å), indicating the good agreement of the model with the experimental data.

First, we discuss the obtained cluster dimers in regard of their core–core distances and geometric structures. Most of the cluster dimers have a distance of the two adamantane cores between 5.9 and 6.5 Å (orange region in Figure 6). The [(PhSi)_4_S_6_] cluster dimers show a core–core distance range that is shifted to slightly smaller values than the range calculated for [(NpSi)_4_S_6_]. The same trend, but even more pronounced, is observed for the {Sn_4_S_6_}‐based cluster dimers. Cluster dimers in this region of inter‐cluster distances yielded stacked or alternating substituents and different orientations of the cluster cores (Figure 7). In all of them, a minimum of two substituents of one cluster interact with at least two substituents of the other cluster. In the “stacked dimer” conformers, the substituents are arranged directly towards each other. In the “alternating dimer” conformers, the substituents of one cluster are located in a void between the substituents of the other cluster. Interestingly, the “alternating dimer” structure is slightly preferred on average for clusters with phenyl substituents, while for naphthyl substituents the “stacked dimer” structure is slightly preferred. The core–core distances between Si/S and Sn/S clusters, respectively, differ only slightly for analogous cluster dimers (by ≈0.15 Å on average). The “alternating dimers” show a high similarity to the arrangement of the cluster in the crystal structures, as demonstrated for [(PhSi)_4_S_6_] in Figure 8. Although similar structural units are present, the core–core distance in the crystal structure (closest core–core distances: 7.05–7.46 Å in **IV**, 7.44–7.58 Å in **1**) is much larger (by 0.5 to 1.0 Å) as compared to the calculated cluster dimers, as in the crystal, the interaction is shared between more than two clusters. Besides the majority of cluster dimers with a core–core distance between 6.0 and 6.5 Å, there are a few cluster dimers with core–core distances between 7.2 and 8.0 Å, with a poor relative orientation of the clusters to each other (green region in Figure 6; example of a structure shown in Figure 7, top). The Si/S cluster dimers with phenyl and naphthyl substituents listed in this green region show larger core–core distances than the corresponding crystal structures. On the other hand, for some of the Sn/S‐based cluster dimers, we found a fusion of cluster structures (purple region in Figure S13). Since we do not consider them to be relevant for the solid‐state structures of this study, we have not considered the fused clusters further.

Second, we discuss the obtained cluster dimers depending on their dissociation energy. Considering each individual cluster composition, the majority of dimer clusters with core–core distances between 5.9 and 6.5 Å yield higher absolute dissociation energies as compared to the absolute dissociation energies of the cluster dimers with core–core distances between 7.2 and 8.0 Å. The difference in dissociation energies between the medium and large core–core cluster dimers is smaller for clusters with Np ligands than for those with Ph ligands.

**Figure 9:**
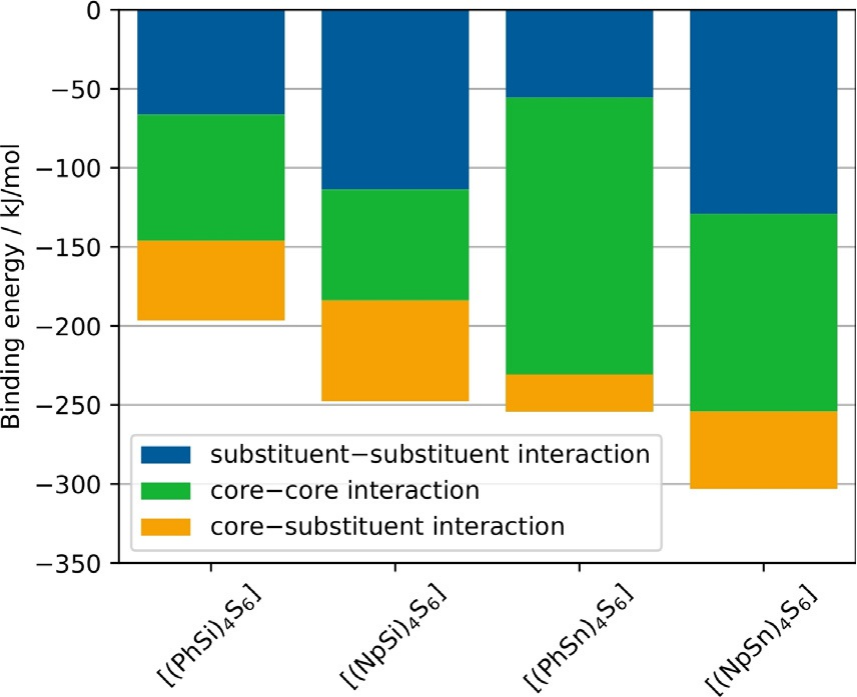
Decomposition of the binding energy contributions of cluster dimers into substituent–substituent interaction, the substituent–core and core–core interaction calculated at the BP86‐D3/cc‐pVDZ(‐PP) level of theory.

Considering the cluster dimers with core–core distances between 5.9 and 6.5 Å, there are 2–3 regions of dissociation energies for the individual cluster compositions in Figure 6, each of which correlates with a certain number of substituents interacting with each other. Thus, the highest dissociation energies were obtained for cluster dimers in which three substituents of one cluster interact with three substituents of the other one. The “stacked dimer” and “alternating dimer” conformers for individual cluster compositions yield similar dissociation energies. Among the cluster dimers with highest dissociation energies, [(NpSi)_4_S_6_] exhibits a dissociation energy that is by about 65 kJ mol^−1^ higher than that of [(PhSi)_4_S_6_]. A similar, but slightly smaller, difference in dissociation energies is calculated for the corresponding {Sn_4_S_6_}‐based cluster dimers (≈53 kJ mol^−1^). Obviously, the larger Np substituents lead to a stronger interaction between cluster dimers than Ph substituents. Other than originally anticipated, the investigation of the cluster dimers did not indicate larger dispersive interaction for clusters comprising a smaller radius of the cluster core. The {Sn_4_S_6_}‐based clusters have a higher absolute dissociation energy than the {Si_4_S_6_}‐based cluster dimers, which illustrates a stronger interaction overall. To understand this result, we performed a decomposition analysis of the cluster dimer binding energy contributions into the substituent–substituent interaction, the substituent–core and core–core interaction (see Figure 9).

The decomposition of the binding energy reveals similar substituent–substituent binding energies for cluster dimers with the same substituent. The larger naphthyl substituents show a higher binding energy than the smaller phenyl substituents due to larger dispersive interactions. In contrast, the core–core binding energy is larger for the Sn/S‐based cluster dimers than for the Si/S based cluster dimers due to the larger adamantane core. This stronger core–core interaction for the larger Sn/S‐based cluster dimers leads to higher absolute values of the dissociation energies than observed for the Si/S‐based analogs. For the cluster dimers with larger core–core distances (green region in Figure 6), the same trend in dissociation energies is observed, although larger core–core distances are found here than for crystalline [(PhSi)_4_S_6_] and [(NpSi)_4_S_6_]. We therefore believe that these insights can be transferred to crystalline and amorphous cluster materials with core–core distances between the medium and large core–core distance regions of Figure 6.

In conclusion, we propose that the predominance of the relatively isotropic core–core interactions in [(PhSn)_4_S_6_] outplay the rather directional interactions involving the substituents, which explains why this compound shows a distinctly lower tendency for order in the solid than the crystalline {Si_4_S_6_}‐based homologue. This trend is also visible for the clusters with Np substituents, yet for [(NpSn)_4_S_6_] the relatively similar strengths of core–core and substituent–substituent interactions allow for a higher degree of intermolecular order, which is in agreement with the (virtually contradicting) experimental findings: the powder produces SHG (as a sign of phase matching) in spite of an apparently amorphous nature according to X‐ray diffraction. The results of this study thus contribute significantly to the overall understanding of the origin of (dis)order in compounds of the type [(RT)_4_E_6_] (T=Si, Ge, Sn; E=S, Se, Te).

### Optical Properties

**Figure 10:**
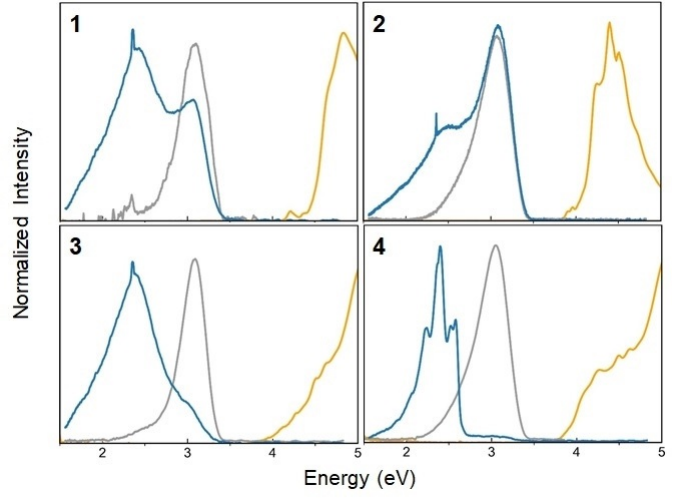
Linear absorption spectra in solution (yellow line) and photoluminescence spectra (solid=blue line, solution=gray line) of compounds (**1**–**4**).

We explored the optical response of compounds **1**–**4** by optical spectroscopy. The absorption and emission spectra are summarized in Figure 10.

Clearly, the emission and absorption data are mirror‐image‐like for the solution data. The emission spectra of the solid phase show an additional resonance about 500 meV below the emission maximum in solution. Such features are commonly attributed to excitons or an ensemble thereof. Notably, the higher‐energy emission maximum persists in the condensed matter phase for all samples. However, it is significantly quenched by the existence of Au/S moieties in compounds **3** and **4** in contrast to **1** and **2**. This apparently enhances the lower‐energy emission channels. In particular, for compound **4**, several distinct emission channels are observed which are tentatively attributed to transitions involving atomic orbitals of the Au^I^ atom.

**Figure 11:**
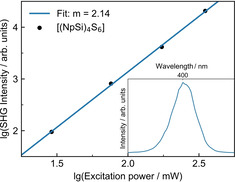
Nonlinear optical response of compound **1**. Excitation power dependency of SHG. SHG spectrum from excitation at 800 nm (inset).

The crystalline compound **1** shows a clear nonlinear optical response. In agreement with the observation made for **IV**,^[7b]^ we observe SHG here in spite of the centrosymmetric space group, which therefore is attributed to surface effects or defects of the crystal (see Figure 11). The other compounds, in general, do not show a nonlinear response that is clearly distinguishable from luminescence effects owing to chemical transformations. Hence, we can neither confirm nor exclude that the adamantane‐based compound **2** exhibits WLG, but its significantly higher chemical sensitivity to oxygen and humidity as compared to the heavier homologue hampers a clear statement to date (note that it is technically not possible to prevent that the samples are exposed to air for a very short moment prior to the measurement).

### Calculation of Optical Properties

We investigated the role of the substituents (phenyl vs. naphthyl) on the optical response of the molecular clusters. Thereby, we modeled two of the synthesized clusters with the same core but different substituents, [(PhSi)_4_S_6_] and [(NpSi)_4_S_6_]. The structural relaxation of single clusters and molecular crystals performed by the plane‐wave implementation of the DFT within the periodic supercell method leads to geometries of the same symmetry and in overall close agreement with the structures calculated with a localized basis. The optimized geometries were employed for the calculation of the electronic and optical excitations.

**Figure 12:**
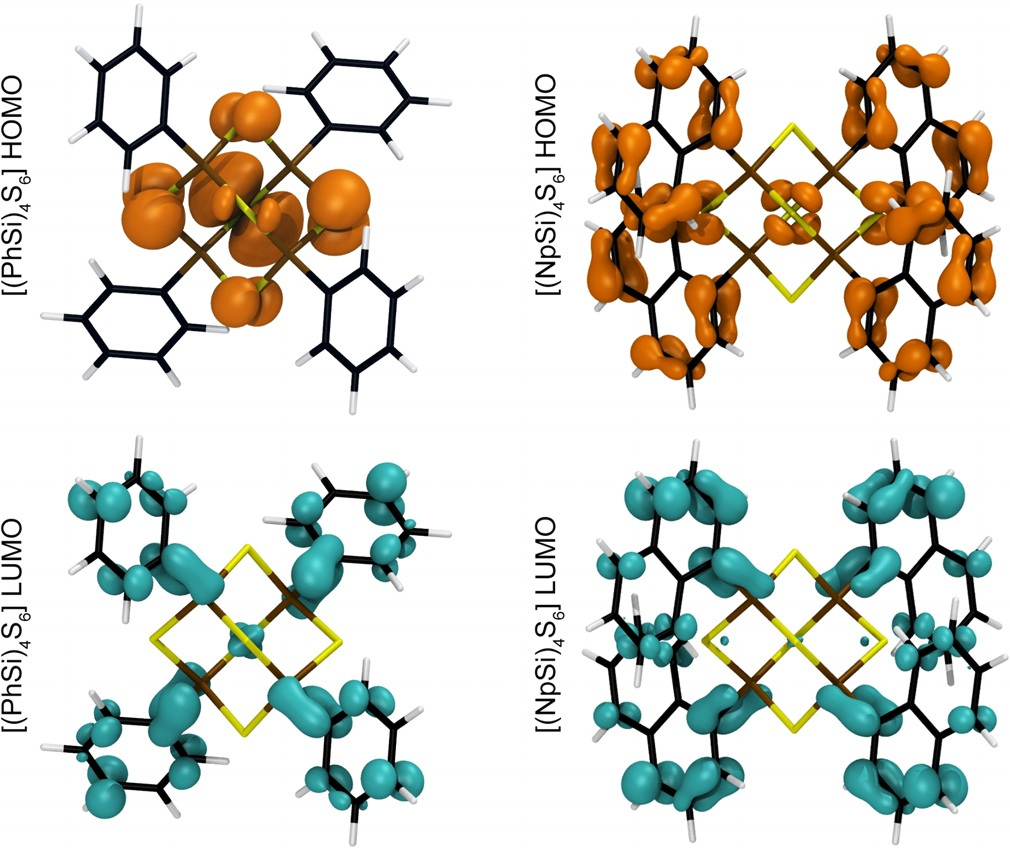
Optimized molecular structures and orbital characters of the HOMO and LUMO states of [(PhSi)_4_S_6_] (left) and [(NpSi)_4_S_6_] (right) single clusters.

Figure 12 shows the relaxed geometry as well as the HOMO and LUMO states for the [(PhSi)_4_S_6_] and [(NpSi)_4_S_6_] clusters. The structural differences between the cluster cores of the two molecules are limited. The Si−S distance amounts to 2.15 Å in both clusters, while the S−C bond length is 1.87 Å in [(PhSi)_4_S_6_] and 1.88 Å in [(NpSi)_4_S_6_]. However, the different substituents have a deeper impact on the molecular electronic structure. The (degenerate) HOMO of the [(PhSi)_4_S_6_] molecule is localized at the S atoms in the cluster core, while the HOMO of the [(NpSi)_4_S_6_] cluster is localized on the naphthyl rings. The LUMO of both systems is similar and localized on the substituents (see Figure 12). Thus, the DFT‐calculated HOMO–LUMO gap of the two systems is rather different and amounts to 3.76 eV for [(PhSi)_4_S_6_] and 2.96 eV for the [(NpSi)_4_S_6_] cluster. Quasiparticle effects, calculated in an approximate manner by the Δ*S*CF method as described in Ref. [13], open up the fundamental independent‐particle‐approximation (IPA) gap to a value of 6.54 eV for [(PhSi)_4_S_6_] and 5.09 eV for the [(NpSi)_4_S_6_] cluster (see Figure S15 in the Supporting Information). The energy of the lowest excitonic excitation, describing the transition of one electron to the LUMO leaving a hole behind in the HOMO, is calculated following the procedure described in Ref. [13]. Excitonic excitations of 3.92 eV for [(PhSi)_4_S_6_] and 2.89 eV for the [(NpSi)_4_S_6_] cluster, respectively, are predicted. These energies are rather close to the HOMO–LUMO gap, suggesting that the quasiparticle shifts are nearly canceled out by the electron‐hole attraction. Thus, as many‐body effects due to the electron‐electron and the electron‐hole interaction counterbalance, the IPA‐calculated optical excitation spectra are expected to yield a reasonable description of the measured optical response, at least for the low‐energy excitations.

**Figure 13:**
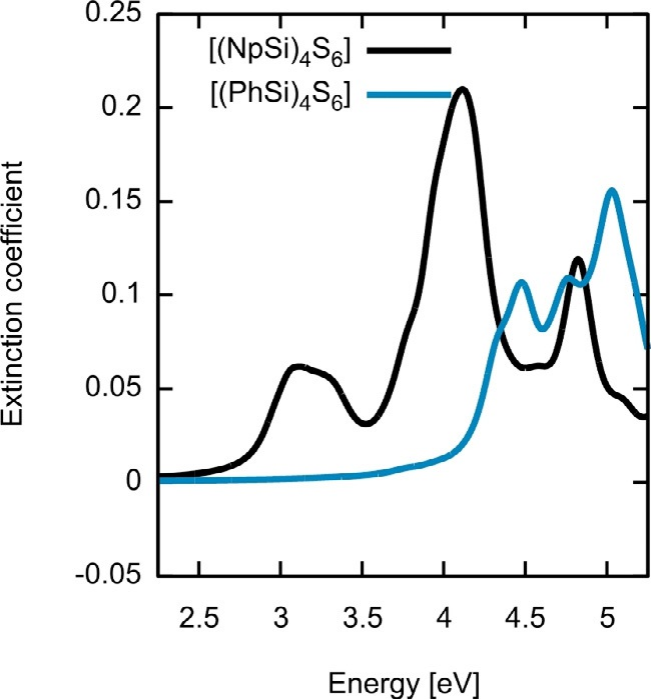
Extinction coefficients calculated in the independent‐particle approximation for isolated [(PhSi)_4_S_6_] (blue line) and [(NpSi)_4_S_6_] (black line) clusters.

The extinction coefficient of [(PhSi)_4_S_6_] (blue line) and [(NpSi)_4_S_6_] (black line) single clusters is shown in Figure 13. The components of the dielectric tensor are averaged to allow for a direct comparison with experimental data later in the manuscript. The general shapes of the dielectric functions are rather different, although the structures of the two clusters are similar. In order to rationalize these differences and understand their origin, we analyzed the electronic transitions of the two clusters. Indeed, spectral resonances can be directly related to electronic transitions between occupied and empty states at the IPA level.

The onset of the optical absorption corresponds exactly to the DFT‐calculated HOMO–LUMO transition in the case of the [(NpSi)_4_S_6_] cluster (black line). Structures in the first peak match quite well the energy of the HOMO–LUMO+1,2 (degenerate) and HOMO–LUMO+3. The main peak at about 4 eV is mainly due to transitions from the HOMO to a group of energetically close states localized at the C atoms of the naphthyl rings. Instead, in the case of the [(PhSi)_4_S_6_] cluster (blue line), the onset of the optical absorption is located at much higher energies than the DFT‐calculated HOMO–LUMO difference. Indeed, the HOMO–LUMO transition probability is small, due to the different spatial localization and orbital character of the two states. We assign the first spectral peak to transitions from the HOMO to three molecular orbitals about 4.4 eV above the HOMO and with a spatial extension partially overlapping the HOMO. Summarizing, the absorption spectra of [(PhSi)_4_S_6_] and [(NpSi)_4_S_6_] clusters are rather different concerning the positions and line shapes of the spectral features. The deviations in the optical response originate from the qualitatively different and differently localized electronic states involved in the transitions resulting in the main spectral peaks of the two compounds.

**Figure 14:**
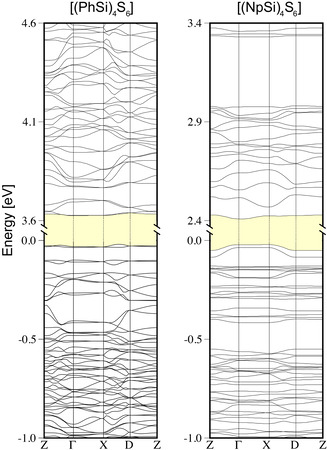
Electronic structure of the crystals comprising [(PhSi)_4_S_6_] and [(NpSi)_4_S_6_] molecules calculated within the IPA. The fundamental band gap is indicated in yellow.

The optical answer of the isolated clusters represents the basis for understanding the absorption spectra of the solid material comprising the molecules. According to the calculations, which started from the experimental crystal data, the optimized geometries show only small modifications of the geometrical parameters determined for the isolated molecules. The largest bond length deviation is 1.4 pm, while the bond angles of the substituents are rotated by 13.2° with respect to the single molecules due to the presence of toluene. When the single clusters aggregate to form the crystalline material, the discrete energy levels broaden to become energy bands. These have been calculated in the IPA approximation along the directions in reciprocal space shown in Figure S16, and are illustrated in Figure 14. The electronic band gap of the crystals is slightly smaller than the HOMO–LUMO energy differences of the corresponding molecules; however, the orbital characters of valence and conduction band edges closely resembles those of the HOMO and LUMO of the parent molecules (see Figure 12). As the band dispersion is rather flat and no major rearrangement is expected upon inclusion of quasiparticle effects, self‐energy correction mainly serves to widen the band gap, and may be replaced by a numerically less costly scissors‐shift for the calculation of the optical response.

**Figure 15:**
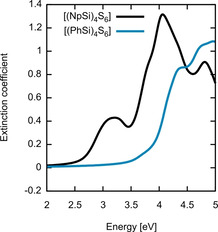
Extinction coefficient of the [(PhSi)_4_S_6_] derived (blue line) and [(NpSi)_4_S_6_] derived (black line) molecular crystals calculated within the IPA at the DFT equilibrium geometry.

We then proceeded with the optical characterization of the synthesized crystalline compounds. Owing to the synthesis method, the experimental crystal structure of [(NpSi)_4_S_6_] (**1**) comprises toluene molecules (0.5 C_7_H_8_ per formula unit of **1**, see Table S1). As illustrated in Figure S17, the extinction coefficient calculated with and without toluene does not substantially differ, demonstrating that the optical response of the molecular cluster is not affected by the presence of solvent. The extinction coefficient calculated within the IPA for the [(PhSi)_4_S_6_] (**IV**) derived and [(NpSi)_4_S_6_] (**1**) derived molecular crystals is shown in Figure 15.

For both compounds, the spectral signatures of the isolated clusters are clearly recognizable in the optical answer of the corresponding molecular crystals. They barely shift in energy, and the relative intensity compares well with that predicted for the single clusters. It can thus be concluded that the transitions leading to the absorption peaks of the single clusters are chiefly responsible for the major peaks in the dielectric function of the semiconducting crystals. The main difference between molecules and crystals comprising these molecules is that the crystal absorption features are broadened with respect to the molecular peaks due to the energy dispersion of the molecular states upon aggregation. As the onset of the molecular and crystalline absorption coincides, we can exclude new intermolecular low‐energy electronic transitions to occur when the clusters aggregate to form crystals. Thus, for the investigated compounds, the molecules seem to individually interact with light rather than jointly. However, the relative orientation of the molecules in the solids (ordered vs. non‐ordered) controls the nature of the nonlinear optical response (with or without phase matching).

**Figure 16:**
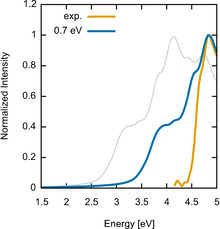
Extinction coefficient of the crystals comprising [(NpSi)_4_S_6_] molecules, calculated within the IPA with scissor‐shifts of 0.7 eV (blue line). The measured curve (yellow line) as well as the IPA calculation without scissors‐shift (gray line) are shown for comparison.

In order to compare the calculated spectra of the crystalline material comprising [(NpSi)_4_S_6_] cluster (**1**) with the measured data, we accounted for many‐body effects in an approximated manner by means of a scissors‐shift.^[14]^ We chose a scissor‐shift of 0.7 eV, correcting the DFT underestimation of the experimentally measured band gap of the molecular crystals. The corresponding results are shown by the blue line Figure 16 together with experimental results (yellow line) from Figure 10. The comparison with the experimental data shows that calculations at the IPA level with scissors‐shifts already agree with the measurements.

The calculations fairly describe the main absorption peak, originating from transitions between the valence band edge and bands localized at the C atoms of the naphthyl rings, including the shoulder observed at about 4.6 eV. However, the intensity of the peak at about 3.5 eV corresponding to the transition from the valence band maximum to the conduction band minimum is overestimated in the calculations in comparison with the measured spectra. More refined and computationally demanding approaches accounting for quasiparticle effects in a perturbative manner and accounting for the electron‐hole attraction by means of the Bethe‐Salpeter equation might further improve the agreement with the experiment.

The calculation of the second‐order polarizability tensor for the crystalline phases of [(PhSi)_4_S_6_] (**IV**) and [(NpSi)_4_S_6_] (**1**) shows vanishing optical nonlinearities for all tensor components, as expected for a centrosymmetric crystal structure. Deviations from this behavior in experiments thus arise from structural defects and amorphous regions in the samples, as well as from surface‐related contributions. At least for centrosymmetric crystals, they are not inherent bulk properties.

## Conclusion

We presented the synthesis of new members of the rare class of organosilicon sulfide clusters of the general formula [(RSi)_4_S_6_]. We added new members with R=naphthyl (Np, **1**) and styryl (Sty, **2**), which were investigated with respect to their reactivity towards [AuCl(PPh_3_)], their structures, and their optical properties by a combination of comprehensive experimental and theoretical studies. The investigations contributed to characterize and further understand the chemical behavior of such clusters, which showed to behave differently from the much better known germanium and tin homologues. Our studies indicate a much more pronounced tendency to polymerization in case of **2** than observed for its Sn homologue, and confirm the expected, much higher air and water sensitivity of the Si compounds. Second, the observation that clusters with the combination Ph/Si/S (reported previously) and Np/Si/S (**1**) are crystalline, while the Sn homologues are amorphous was studied on the bases of cluster dimer models calculated with DFT methods. Different types of intra‐cluster interactions were found, that led to different dissociation energies as a function of the nature of the substituents, their relative orientation, and the core compositions {Si_4_S_6_} versus {Sn_4_S_6_}. The detailed study ultimately helped to explain the experimental findings on the basis of much stronger isotropic interactions in the {Sn_4_S_6_}‐based cases in comparison with directional interactions that play a much more notable role in the {Si_4_S_6_}‐based materials. Regarding the optical properties of the new compounds and the products of their reaction with the gold complex, we could show that the emission is quenched upon fragmentation and formation of the gold compounds. Moreover, the crystalline compound **1** was proved to exhibit SHG as a consequence of high order that allows for phase matching; however, the SHG signal does not stem from the crystalline bulk but should be attributed to surface phenomena or structural defects. The behavior of **2** is clearly different, but its chemical nature inhibited a statement regarding the compound's nonlinear response. The experimental findings were corroborated by static and time‐dependent DFT calculations of both the molecules and the crystalline material, which are in full agreement with the measurements and therefore indicate the applicability of the used methods and models.

## Conflict of interest

The authors declare no conflict of interest.

## Supporting information

As a service to our authors and readers, this journal provides supporting information supplied by the authors. Such materials are peer reviewed and may be re‐organized for online delivery, but are not copy‐edited or typeset. Technical support issues arising from supporting information (other than missing files) should be addressed to the authors.

Supplementary
