# Supplementary material for: Towards Understanding the Reactivity and Optical Properties of Organosilicon Sulfide Clusters
Source: Angew Chem Int Ed Engl. 2020 Dec 15;60(3):1176–86. doi: 10.1002/anie.202011370 (PMC7839700; doi:10.1002/anie.202011370)
Supplement: Supplementary file 1 — Supplementary [file ANIE-60-1176-s001.pdf]

Angewandte Chemie
Eine Zeitschrift der Gesellschaft Deutscher Chemiker

Supporting Information

Towards Understanding the Reactivity and Optical Properties of Organosilicon Sulfide Clusters

Katharina Hanau, Sebastian Schwan, Moritz R. Schäfer, Marius J. Müller, Christof Dues, Niklas Rinn, Simone Sanna, Sangam Chatterjee,* Doreen Mollenhauer,* and Stefanie Dehnen**

anie_202011370_sm_miscellaneous_information.pdf

1 Methods

General synthetic methods: All syntheses were performed under exclusion of air and moisture using standard Schlenk techniques. All solvents were dried and freshly distilled prior to use. $NpSiCl_3$,[1] $StySiCl_3$,[2] $[(PhSi)_4S_6]$,[3] Na_2S,[4] and $[AuCl(PPh_3)]$[5] were prepared according to literature procedures. Further chemicals were purchased from Sigma Aldrich.

Spectroscopy and spectrometry: ^{1}H, ^{13}C, ^{29}Si, and ^{31}P NMR spectra were recorded with a Bruker AV 500 (**2**, **3**, **4**) or a Bruker AV III HD 300 (**1**) spectrometer. The chemical shifts are given in ppm relative to the residual protons of deuterated solvents for ^{1}H spectra and relative to the solvent signal for ^{13}C spectra. ^{13}C, ^{29}Si, and ^{31}P spectra were measured with ^{1}H decoupling. LIFDI mass spectra were recorded with an AccuTOF GCv mass spectrometer (JEOL). Micro X-ray fluorescence spectroscopy (µ-XRF) was carried out using a Bruker M4 Tornado spectrometer with an Rh target X-ray tube, poly-capillary optics and a Si drift detector. Elemental analysis was performed on an Elementar vario MICRO CUBE apparatus.

Single-crystal X-ray diffraction: Crystals suitable for X-ray diffraction were investigated with a STOE STADIVARI (**II**, **1**) or a STOE IPDS2 (**3**, **4**) diffractometer at 100 K. The STADIVARI uses Cu-K_α radiation (λ = 1.54186) from an X-ray micro source with X-ray optics and a Pilatus 300K Si hybrid pixel array detector. The IPDS2 uses Mo-K_α radiation and a graphite monochromator (λ = 0.71073). Upon spherical (STADIVARI) or numerical (IPDS2) absorption correction and scaling (STOE LANA), the structure solution was performed by direct methods followed by full-matrix-least-squares refinement against F^2, using SHELXT15, SHELXL15, and OLEX2 software.[6]

Single crystals of $NpSiCl_3$ (II) suitable for X-ray diffraction were isolated after distillation of the product.

Synthesis of $[(NpSi)_4S_6]$ (1): $NpSiCl_3$ (2.277 g, 8.7 mmol) was dissolved in 5 mL THF and Na_2S (1.019 g, 13.1 mmol) was suspended in 17 mL THF. Both flasks were cooled to 0 °C and the $NpSiCl_3$-solution was added slowly to the Na_2S-suspension. The reaction mixture was stirred for 18 h and allowed to warm to room temperature. After removal of the solvent *in vacuo*, the resulting white solid was extracted with 36 mL toluene and NaCl was filtered off. The volume of the filtrate was reduced *in vacuo* until the first crystals formed and the filtrate was stored at -25 °C for 2 d to obtain **1** as a colorless, crystalline solid. Some solid was redissolved in toluene to yield single crystals of **1** suitable for single crystal x-ray diffractometry. Yield: 22.1%. ^{1}H NMR (300 MHz, C_6D_6, 25 °C): δ = 2.11 (s, 1.5H, CH_3 (Tol)), 7.02 (m, 1H, *o-/p*-Tol), 7.13 (m, 1H, *m*-Tol), 7.30-7.35 (m, 2H, Np), 7.54-7.62 (m, 3H, Np), 8.32-8.34 (m, 1H, Np), 9.34-9.40 (m, 1H, Np) ppm. ^{13}C NMR (75.5 MHz, C_6D_6, 25 °C): δ = 21.4 (s, Tol), 125.2 (s, Np), 125.7 (*p*-Tol), 126.1 (s, Np), 127.3 (s, Np), 128.6 (s, *m*-Tol), 129.3 (s, *o*-Tol), 129.6 (s, Np), 131.5 (s, Np), 133.4 (s, Np), 134.4 (s, Np), 135.8 (s, Np), 137.9 (s, *i*-Tol) ppm. **^{29}Si NMR** (59.65 MHz, C_6D_6, 25 °C): δ = 6.8 (s) ppm. **HR-LIFDI-MS:** $C_{40}H_{28}S_6Si_4$ (**1**): calc. 811.95923, found: 811.95619. 0 **Elemental analysis:** Calculated for $C_{40}H_{28}S_6Si_4 \cdot 0.5(C_7H_8)$: C 60.79, H 3.75, S 22.38; found: C 60.70, H 3.934, S 19.351. The relatively large deviation of S is a systematic error found for our sulfide clusters.

Synthesis of $[(StySi)_4S_6]$ (2): Na_2S (0.4984 g, 6.39 mmol) was suspended in 10 mL THF and $StySiCl_3$ (1.012 g, 4.26 mmol) was added slowly at 0 °C. The reaction mixture was stirred for 18 h and allowed to warm to room temperature. After removal of the solvent *in vacuo*, the resulting colorless "oil" was extracted with 20 mL toluene and NaCl was filtered off. The solvent was removed under reduced pressure to yield **2** as crude product. Yield: 85.9%. **^{1}H NMR** (500.2 MHz, C_6D_6, 25 °C): δ = 5.07 (d, J_{HH} = 10.9 Hz, 1H, $C_6H_4CHCH_2$), 5.54 (d, J_{HH} = 17.7 Hz, 1H, $C_6H_4CHCH_2$), 6.43 (dd, J_{HH} = 10.9 Hz, J_{HH} = 17.6 Hz, 1H, $C_6H_4CHCH_2$), 7.19 (d, $^3J_{HH}$ = 8.1 Hz, 2H, *m*-Ph), 7.97 (d, $^3J_{HH}$ = 8.1 Hz, 2H, *o*-Ph) ppm. **^{13}C NMR** (125.8 MHz, C_6D_6, 25 °C): δ = 116.5 (s, vinyl CH_2), 127.0 (s, *m*-Ph), 133.4 (s, *o*-Ph), 134.5 (s, *p*-Ph), 136.4 (s, vinyl CH), 141.6 (s, *ipso*-Ph) ppm. **^{29}Si NMR** (99.4 MHz, C_6D_6, 25 °C): δ = 8.7 ppm. **Elemental analysis:** Calculated for $C_{32}H_{28}S_6Si_4$: C 53.58, H 3.93, S 26.82; found: C 51.95, H 4.104, S

21.933. Elemental analyses did not provide satisfactory result, as it has so far not been possible to isolate the product in a pure (or defined) form owing to its immediate oligomerization/polymerization tendency, and its very different to handle macroscopic consistency (see main text).

Synthesis of [{PhSi(μ-S)}$_2${AuPPh$_3$(μ-S)}$_2$] (3): [(PhSi)$_4$S$_6$] (100 mg, 0.163 mmol), Na_2S (51 mg, 0.652 mmol), and [AuCl(PPh$_3$)] (184 mg, 0.372 mmol) were dissolved in 20 mL dichloromethane at −40 °C. The mixture was stirred and allowed to warm to room temperature for 18 h. After filtration, the colorless solution was layered with *n*pentane to give colorless blocks of **3** (82.5 mg, 20%). Due to the low solubility of the crystals, NMR spectra of **3** could not be recorded. **Elemental analysis:** Calculated for $C_{48}H_{40}Au_2P_2S_4Si_2{\cdot}CH_2Cl_2$: C 43.85, H 3.15, S 9.56; found: C 43.88, H 3.141, S 8.973.

Synthesis of [{NpSi(μ-S)}$_2${AuPPh$_3$(μ-S)}$_2$] (4): [(NpSi)$_4$S$_6$] (114 mg, 0.140 mmol), Na_2S (44 mg, 0.561 mmol), and [AuCl(PPh$_3$)] (139 mg, 0.390 mmol) were dissolved in 20 mL dichloromethane at −40 °C. The mixture was stirred and allowed to warm to room temperature for 18 h. After filtration, the colorless solution was layered with 10 mL *n*pentane to give colorless blocks of **4** (60 mg, 15.8%). **^{29}Si-NMR** (99.4 MHz, CD_2Cl_2, 25 °C): δ = 12.4 ppm. **Elemental analysis:** Calculated for $C_{56}H_{44}Au_2P_2S_4Si_2{\cdot}CH_2Cl_2$: C 47.47, H 3.22, S 8.89; found: C 46.88, H 3.12, S 5.514.

Computational details for the structural studies: Density functional theory (DFT) calculations were performed using the Gaussian09 software package.[7] All structure optimizations and frequency calculations were carried out by applying the generalized gradient approximation density functional BP86 combined with the Grimme dispersion correction with Becke-Johnson damping.[8] All elements with the exception of Sn were described with an all-electron correlation consistent valence double-zeta Dunning-type basis set (cc-pVDZ).[9] For Sn, the effective core potential ECP28MDF in combination with the related cc-pVDZ-PP was applied.[10] Furthermore, density fitting was employed for all calculations. Frequency calculations have been performed for all optimized structures to confirm these as minimum structures. Imaginary frequencies (below 22 cm^{-1}) were obtained for several monomer and dimer cluster structures, however. These were identified as rotational contributions of non-interactive phenyl or naphthyl rings. The clusters with phenyl substituents exhibit smaller imaginary frequencies than naphthyl-substituted clusters, which correlates with the rotation barriers of these substituents.

Rotational scans of the phenyl- and naphthyl groups for optimized single cluster [(PhSi)$_4$S$_6$], [(NpSi)$_4$S$_6$], [(PhSn)$_4$S$_6$], and [(NpSn)$_4$S$_6$] were performed using the built-in scan tool from Gaussian09. The conformers with lowest energy serve for this study. The conformers of the monomers [(PhSi)$_4$S$_6$], [(NpSi)$_4$S$_6$], [(PhSn)$_4$S$_6$], and [(NpSn)$_4$S$_6$] are structurally very similar. One of the four substituents was rotated while the dihedral angles of the other three substituents with respect to the adamantane core were fixed.

Cluster interactions of dimers consisting of two [(PhSi)$_4$S$_6$], [(NpSi)$_4$S$_6$], [(PhSn)$_4$S$_6$], or [(NpSn)$_4$S$_6$] clusters, respectively, were investigated. The starting structures of the cluster dimers were generated for AdPh$_4$ and AdNp$_4$ dimers, with a small adamantane core in order to obtain a variety of conformers (rotation barrier is the largest). The elements, the structure and the core−core distances were adjusted to the desired compound. This way, a large number of starting structures were specified for all compounds.

Two different approaches were used to generate the cluster dimers. First, a conformer analysis was performed with the program CREST[11] with the xTB-GFN2 method,[12] which was developed by Grimme *et al*. With CREST, 70 to 120 conformers of AdPh$_4$ and AdNp$_4$ were obtained due to the flat energy potential curves. The dimer structures with a root-mean-square deviation (RMSD) of atomic positions smaller than 1.1 Å were considered as similar structures and assigned to a group. From each group, the structure with the lowest energy was chosen for the DFT

WILEY-VCH

calculation conducted thereupon. This left 7 conformers for cluster dimers with phenyl substituents and 10 conformers for cluster dimers with naphthyl substituents, for which the core was modified to a larger Si/S or Sn/S adamantane-type cores. The core−core distances were adjusted accordingly. To test this approach, the same calculations were repeated with CREST for [$(NpSi)_4S_6$], and very similar results were obtained.

In order to find additional conformers, cluster dimers were created by using dimer starting structures extracted from the crystal structure of $AdPh_4$.[13] Before optimization, the adamantane core was adjusted to the larger Si/S or Sn/S adamantane-type cores, and the core-core distances were increased to prevent steric hindrance between the phenyl substituents. In addition, the phenyl and naphthyl groups of these cluster dimers were systematically rotated. From the 2178 cluster dimers obtained, 13 dimers of each stoichiometry were randomly chosen for the DFT optimization.

For further characterization of the cluster dimer structures, we calculated the core−core distances as difference between the centers of mass of the monomers. The dissociation energy E_{Diss} was calculated by subtracting the energy of the monomer $E_{\mathrm{Mono,opt}}$ from the total energy of the dimer structure $E_{\mathrm{Dim,opt}}$:

$$E_{\mathrm{Diss}} = E_{\mathrm{Dim,opt}} - 2 \cdot E_{\mathrm{Mono,opt}}$$

Thus, 23 different conformers were determined for the [$(PhSi)_4S_6$] and [$(PhSn)_4S_6$] dimers and 20 different conformers for the [$(NpSi)_4S_6$] and [$(NpSn)_4S_6$] dimers.

The binding energy of the most favorable cluster dimers were calculated by subtracting the energies of the singly calculated monomers in the frozen structure of the dimer from the cluster dimer energy. To gain a deeper insight into the individual binding energy contributions, we calculated the binding energy of the core−core interaction and the substituent−core interaction. Starting from the optimized structure of the most favorable cluster dimer, we removed the substituents and added hydrogen atoms at the right distance and direction to the cluster cores. The binding energy of the frozen-core structures were calculated as descripted above. This procedure was repeated under consideration of the substituents. The outermost substituent was removed, because it does not contribute to the interaction. In order to calculate the binding energy between the substituents of the first cluster and the core of the second cluster, we subtracted the core−core binding energy and the substituent−core binding energy from the total binding energy. With this procedure, we assume to obtain a realistic estimate of the different binding energy contributions for cluster dimers.

Optical spectroscopy: The linear absorption data were acquired in a Cary 3 Bio UV vis spectrometer; compounds (**1**) and (**2**) were dissolved in THF, compounds (**3**) and (**4**) were dissolved in DCM. For the corresponding photoluminescence data, the solutions were excited using 0.9 mW of a frequency-quadrupled Nd:Yag laser emitting ns pulses at 4.66 eV (266 nm). The photoluminescence was imaged onto a 32 cm Cerny-Turner-type Spectrograph equipped with an open-electrode CCD camera. The same setup was used for measuring the solid state compounds, where the samples were encapsulated in fused silica slides.

The nonlinear optical response was measured using a Ti:sapphire laser oscillator emitting 80-fs pulses at a repetition rate of 78 MHz. Powers up to 350 mW impinged on the sample in a confocal microscope using a 0.6 NA long working distance objective lens. The second harmonic was spectrally separated using a 328 mm Cerny-Turner-type spectrograph and detected using a back-illuminated UV-VIS optimized CCD TE deep-cooled camera.

Computational details for the optical response: The optical response of [$(PhSi)_4S_6$] (**IV**) and [$(NpSi)_4S_6$] (**1**) was calculated with the Vienna Ab initio Simulation Package (VASP 5.4.4),[14] a plane-wave implementation of the DFT. The electron-ion interaction is described within the projector-augmented-wave formalism (PAW).[15] The exchange-

WILEY-VCH

correlation (XC) functional is parametrized in the popular version proposed by Perdew-Burke-Ernzerhof (PBE) of the generalized gradient approximation (GGA),[16] which represents a well-tempered balance between computational efficiency, numerical accuracy, and reliability. The number of valence electrons that were employed for the simulation of the H, C, S, and Si atoms amounted to 1 ($1s^1$), 4 ($2s^2\ 2p^2$), 6 ($3s^2\ 3p^4$) and 4 ($3s^2\ 3p^2$), respectively. Structural optimization was performed with an energy cutoff of 400 eV for the plane wave expansion of the wave functions and a mesh of 4×4×2 k points to sample the Brillouin zone. The ionic relaxation was performed starting from the positions as determined by X-ray diffraction until the forces are smaller than 1 meV/Å. As (semi)local XC functionals failed to correctly describe the long-range vdW interactions, we applied a semi-empirical DFT-D3 correction scheme with zero damping,[8a] which has been found to be sufficiently accurate. These parameters are thus on the same footing as in our previous study[17] and ensure well converged structures. We used the formalism proposed by Gajdoš et al. to calculate the imaginary part of the frequency dependent dielectric function by a summation over empty states.[18] The real part is derived by means of the Kramers-Kronig relations. The number of electronic states considered in the calculations was increased to n >2000 for both [$(PhSi)_4S_6$] and [$(NpSi)_4S_6$] for the calculation of the linear optical properties. Thus, all electronic states within a distance of at least 25 eV from the Fermi energy were included. As standard DFT calculations typically yield significantly too small band gaps, quasiparticle or scissors shifts deduced from the experimental HOMO-LUMO gap were applied to widen the [$(NpSi)_4S_6$] band gap. For the comparison with measured optical spectra we used real and imaginary parts of the dielectric function, $\varepsilon_r(\omega)$ and $\varepsilon_i(\omega)$, respectively. We obtained the extinction coefficient κ using the approximation

$$\kappa = \sqrt{\sqrt{\varepsilon_r^2 + \varepsilon_i^2} - \varepsilon_r(\omega)}$$

Our calculations were compared with optical absorption measurements in powder samples. In order to allow for comparison with the experimental data we averaged over the three Cartesian directions to

$$\varepsilon(\omega) = \frac{1}{3}\sum_{i=x,y,z} \varepsilon_{ii}(\omega).$$

The nonlinear optical coefficients were calculated at the IPA level both postprocessing the wave functions and eigenvalues from the electronic structure calculations as the sum of two-band and three-band contributions following the approach described in ref. [19] and from the Berry-phase formulation of the dynamical polarization within the real-time approach described in ref. [20].

WILEY-VCH

2 NMR Spectroscopy

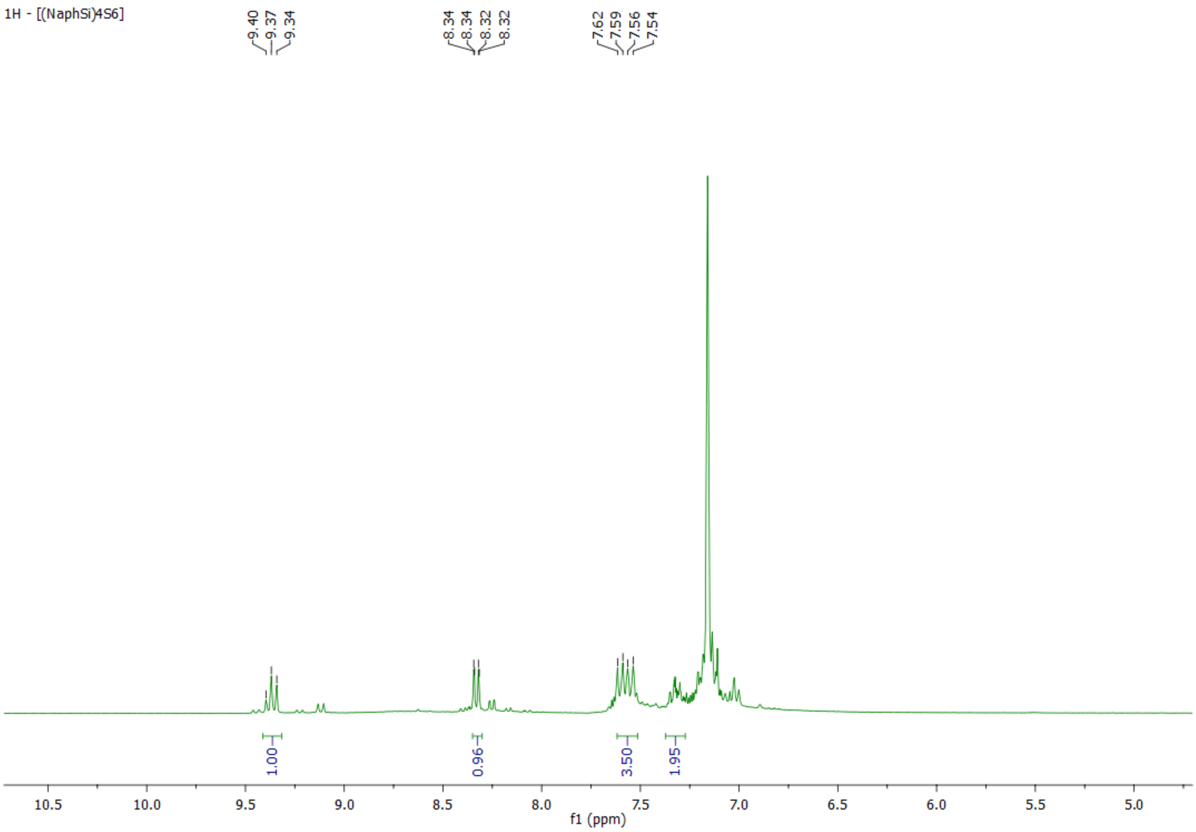

Figure S1. ^{1}H NMR spectrum of **1**.

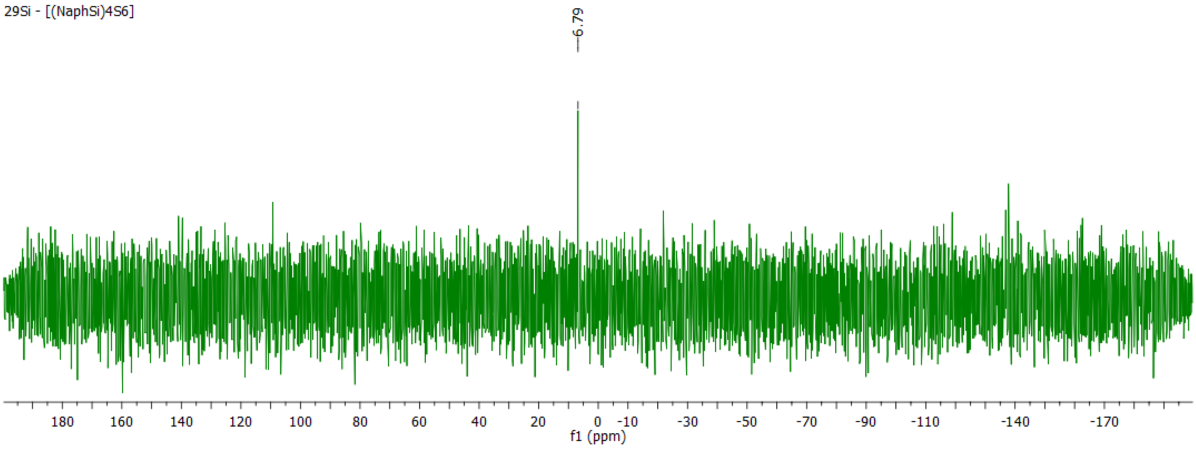

Figure S2. ^{29}Si NMR spectrum of **1**.

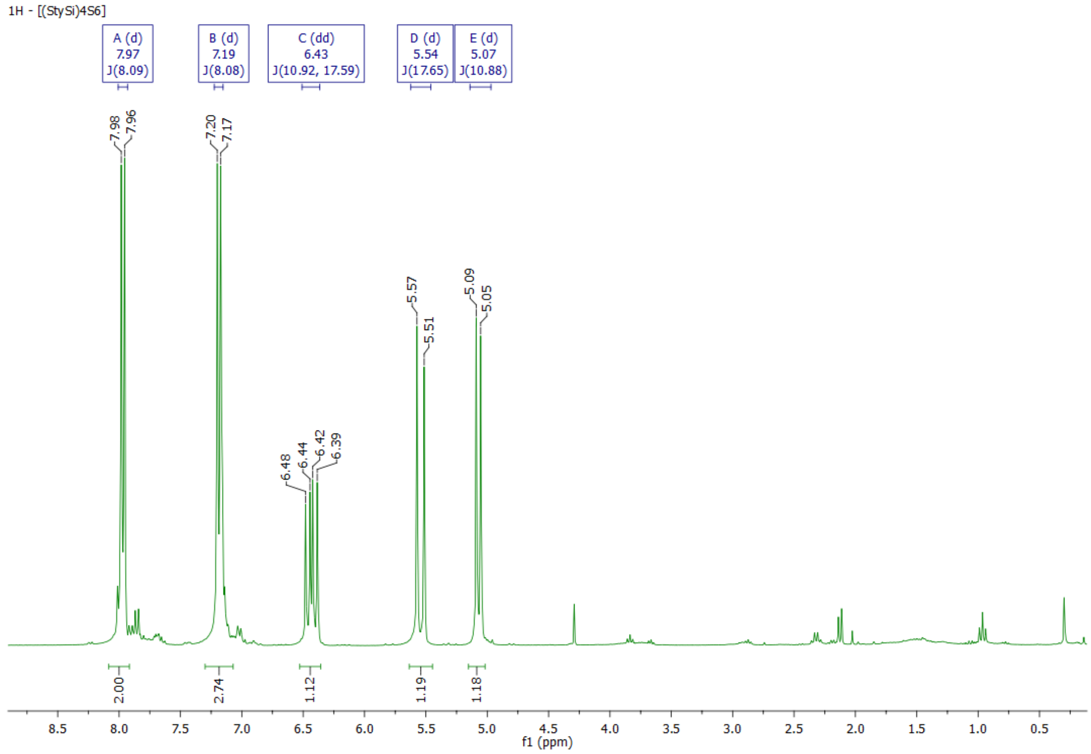

Figure S3. ^{1}H NMR spectrum of **2**.

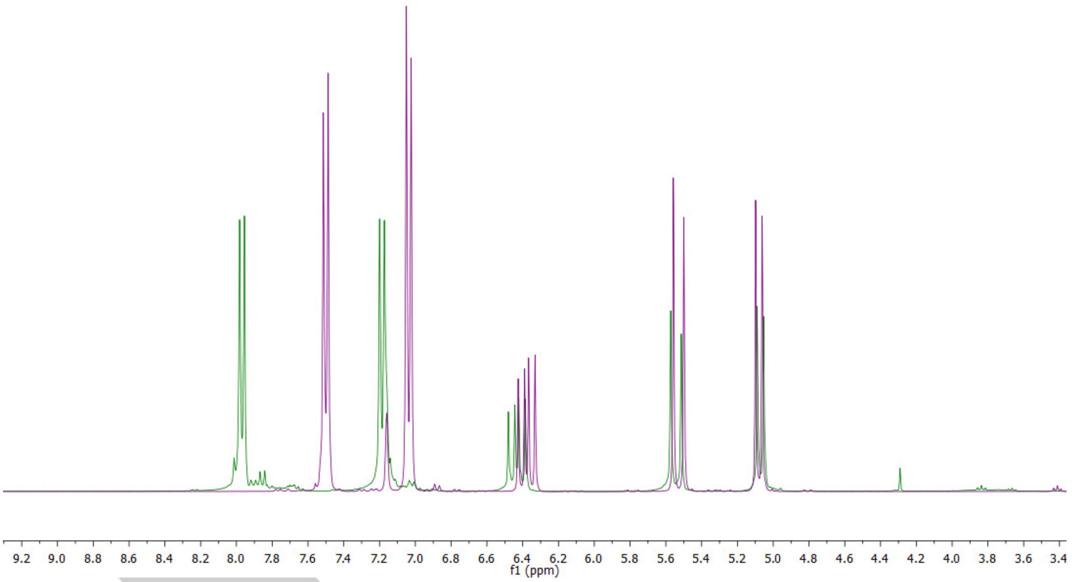

Figure S4. Comparison of the ^{1}H NMR spectra of **III** (purple) and **2** (green).

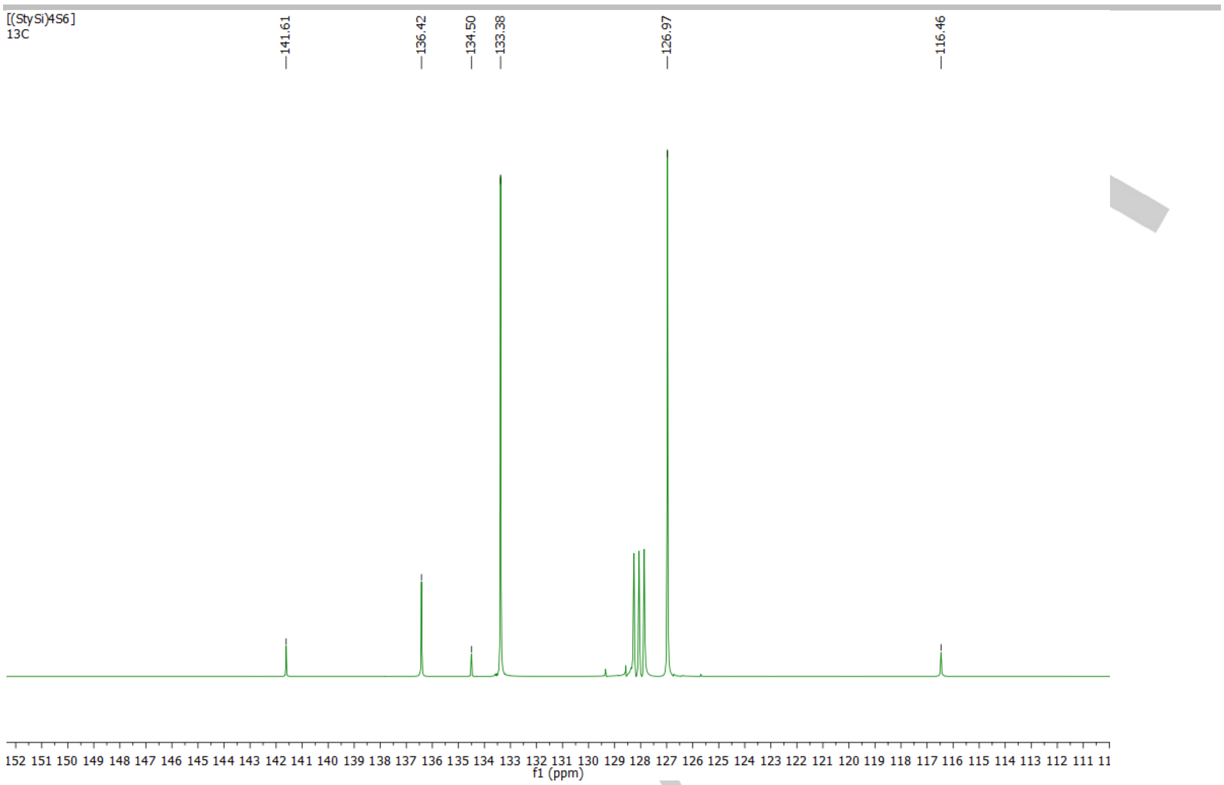

Figure S5. ^{13}C NMR spectrum of **2**.

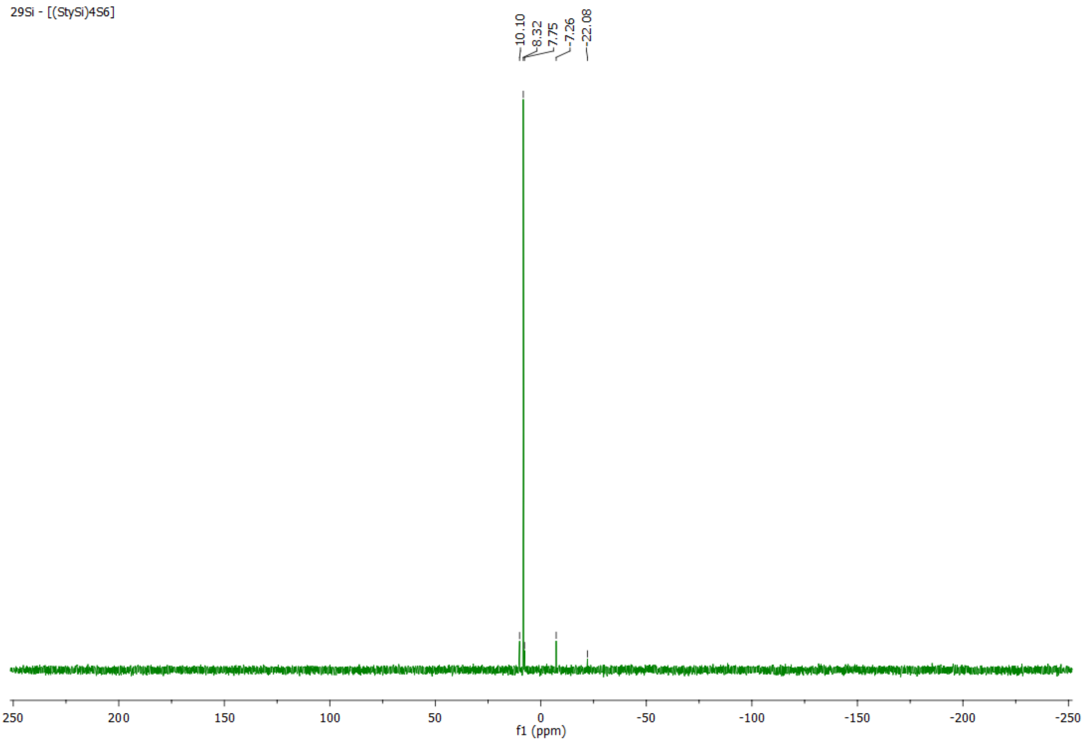

Figure S6. ^{29}Si NMR spectrum of **2**.

3 Mass Spectrometry

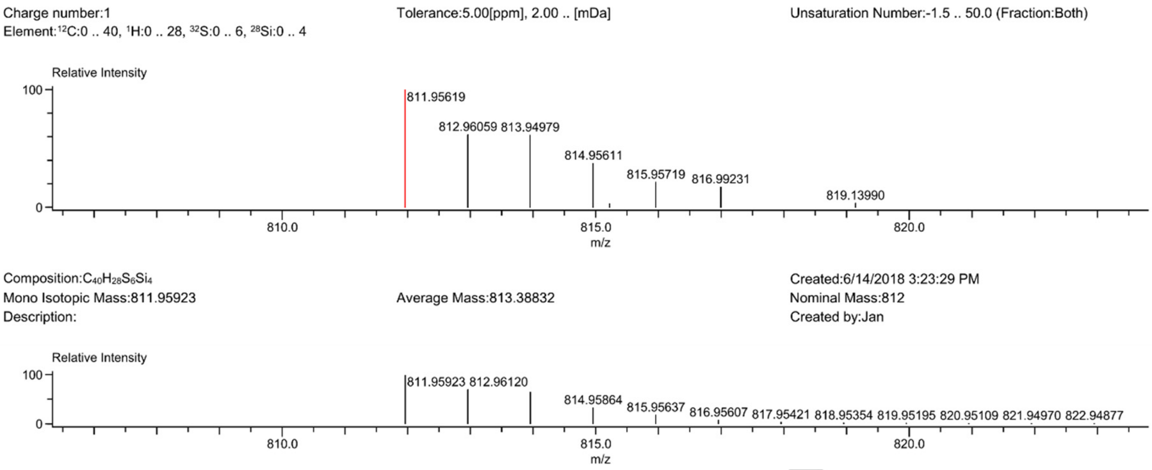

Figure S7. LIFDI mass spectrum of **1**.

WILEY-V

4 Details of the X-ray Diffraction Measurements, Structure Solution, and Refinements

Table S1. Crystallographic data and refinement results of **II** and **1**.

Compound	**II**	**1**·$0.5C_7H_8$
Empirical Formula	$C_{10}H_7Cl_3Si$	$C_{40}H_{28}S_6Si_4$, $0.5(C_7H_8)$
Formula weight /g·mol^{-1}	261.60	859.41
Crystal color and shape	colorless block	colorless block
Crystal size /mm³	0.106 x 0.103 x 0.102	0.1 x 0.1 x 0.01
Crystal system	monoclinic	triclinic
Space group [Flack parameter]	$P2_1/c$	$P\bar{1}$
a /Å	9.9459(3)	13.4738(3)
b / Å	9.0919(2)	13.9032(4)
c / Å	12.3406(4)	24.0970(5)
α /°	90	99.824(2)
β /°	93.103(2)	104.702(2)
γ /°	90	95.208(2)
V / Å³	1114.29(6)	4259.46(18)
Z	4	4
ρ_{calcd} / g·cm^{-3}	1.559	1.340
$\mu_{(Cu\ K\alpha)}$ / mm^{-1}	8.110	4.285
Absorption correction type	sphere	sphere
Min./max. transmission	0.0319 / 0.1521	0.1544 / 0.6874
2θ range / deg	8.904 – 142.904	6.516 – 129.992
No. of measured reflections	10819	100192
R(int)	0.0300	0.0668
Independent Reflections	2138	100192
Independent Reflections ($I>2\sigma(I)$)	1688	36510
No. of parameters	1271	966
R_1 ($I>2\sigma(I)$) / wR_2 (all data)	0.0228 / 0.0509	0.0464 / 0.1136
S (all data)	0.859	0.504
Max. peak / hole / e$^-$ Å³	0.44 / –0.23	0.55 / −0.35
CCDC number	2015922	2015925

WILEY-VCH

Crystal Structure of II

The highest peak of residual electron density on the difference Fourier map (0.44 e⁻/ Å^3) is found 1.01 Å from Si1. Excerpts of the crystal structure of **II** are shown in Figure S8.

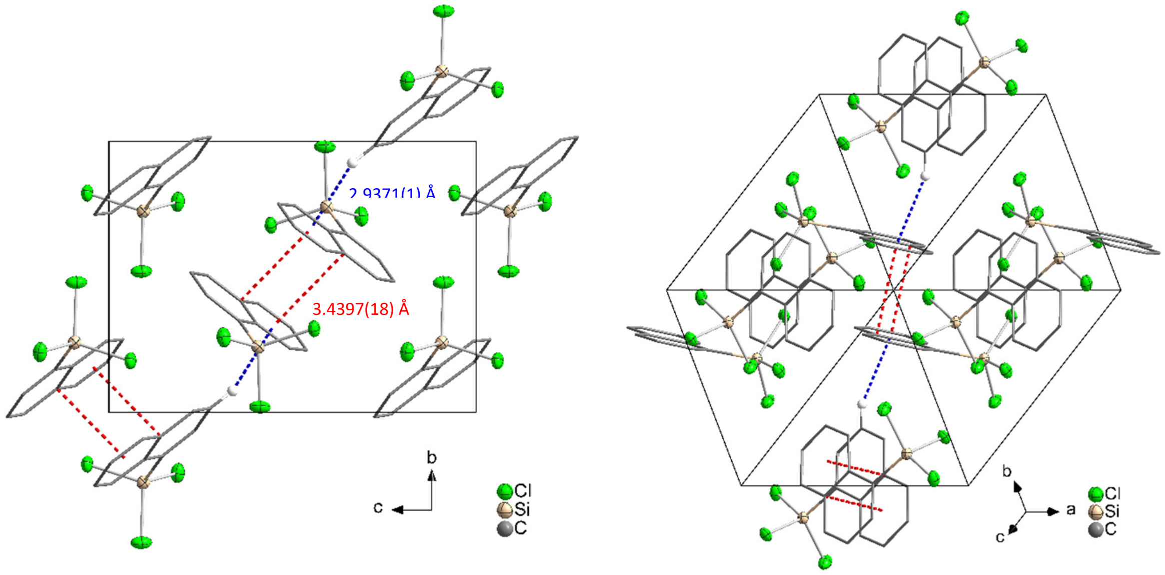

Figure S8. Excerpt of the crystal structure of **II** viewed along the *a* axis (left) and along [1,1,1] (right); π-stacking and CH/π-interactions are indicated as red and blue dashed lines, respectively.

WILEY

Table S2. Fractional atomic coordinates (×10[4]) and equivalent isotropic displacement parameters (Å[2]×10[3]) for **II**. U(eq) is defined as 1/3 of the trace of the orthogonalised U_{IJ} tensor.

Atom	*X*	*y*	*z*	U(eq)	Atom	*x*	*y*	*z*	U(eq)
Cl1	1528.9(5)	9799.2(5)	4115.1(4)	32.63(13)	C4	4775(2)	4943(2)	2687.4(15)	27.0(4)
Cl2	-468.3(4)	7229.6(6)	3124.8(4)	29.33(12)	C5	5077.7(18)	5879.2(19)	3588.3(15)	21.6(4)
Cl3	848.8(4)	6906.9(5)	5588.4(3)	25.61(12)	C6	6398.5(19)	5977(2)	4071.3(16)	25.9(4)
Si1	1242.8(5)	7579.7(5)	4063.6(4)	19.42(11)	C7	6682.1(19)	6857(2)	4947.6(16)	26.0(4)
C1	2685.0(18)	6591.5(18)	3533.0(14)	19.6(4)	C8	5648.7(18)	7694(2)	5381.5(15)	24.1(4)
C2	2456(2)	5661(2)	2659.4(15)	24.0(4)	C9	4361.6(18)	7630.0(19)	4932.4(14)	21.3(4)
C3	3503(2)	4836(2)	2234.2(15)	27.9(4)	C10	4026.3(18)	6723.0(18)	4023.4(14)	19.0(4)

Table S3. Bond lengths for **II** [Å].

Cl3−Si1	2.0367(6)	C10−C5	1.425(2)	C9−C8	1.368(3)
Cl2−Si1	2.0313(7)	C10−C9	1.417(3)	C2−C3	1.407(3)
Cl1−Si1	2.0385(7)	C1−C2	1.379(3)	C4−C3	1.359(3)
Si1−C1	1.8426(18)	C5−C4	1.419(3)	C7−C6	1.362(3)
C10−C1	1.440(3)	C5−C6	1.416(3)	C7−C8	1.408(3)

Table S4. Bond angles for **II** [°].

Cl3−Si1−Cl1	107.58(3)	C9−C10−C5	117.73(16)	C8−C9−C10	121.35(17)
Cl2−Si1−Cl3	106.42(3)	C10−C1−Si1	122.11(13)	C1−C2−C3	121.61(18)
Cl2−Si1−Cl1	106.51(3)	C2−C1−Si1	118.55(14)	C3−C4−C5	121.21(17)
C1−Si1−Cl3	111.98(6)	C2−C1−C10	119.30(16)	C6−C7−C8	119.69(17)
C1−Si1−Cl2	111.42(6)	C4−C5−C10	119.29(17)	C7−C6−C5	121.25(17)
C1−Si1−Cl1	112.56(6)	C6−C5−C10	119.38(17)	C9−C8−C7	120.61(17)
C5−C10−C1	118.62(16)	C6−C5−C4	121.33(16)	C4−C3−C2	119.97(18)
C9−C10−C1	123.64(15)				

WILEY-VCH

Crystal Structure of 1

The studied crystal was twinned. Therefore, the twin data refinement was carried out with a batch scale factor of 0.4770(6). Some of the co-crystallized solvent molecules could not be found/refined due to their disorder. They were thus detracted from the data by the back-Fourier-transform method.[21] The void is located at (0.000, 0.500, 0.000), has a volume of 357 Å^3 and contains 93 electrons, which amounts to a bit less than 2 molecules of toluene. The highest peak of residual electron density on the difference Fourier map (0.55 e$^-$/ Å^3) is found 3.991 Å from C44. Excerpts of the crystal structure of **1** are shown in Figure S9.

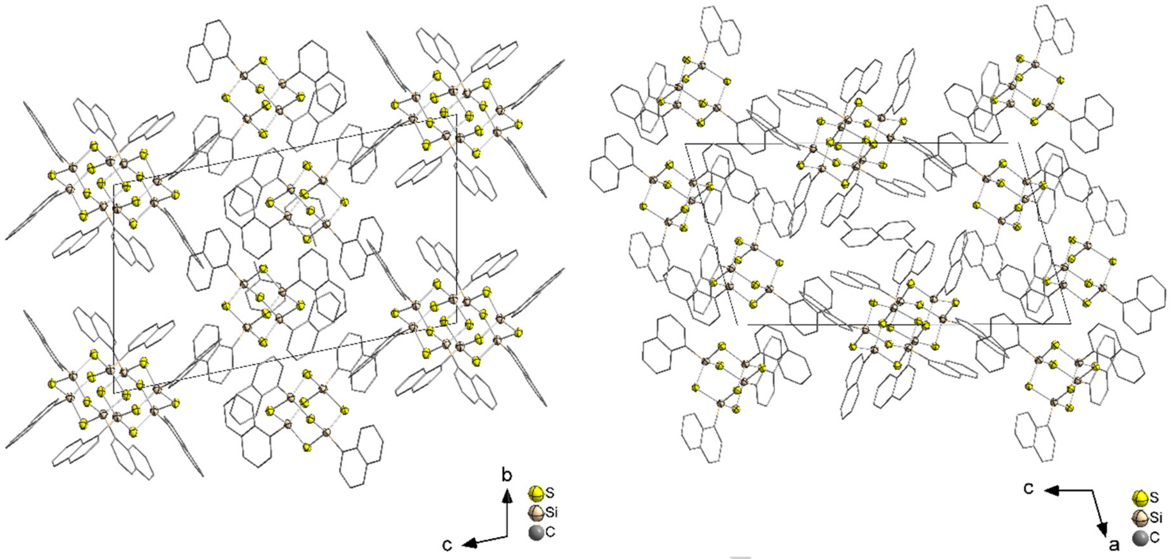

Figure S9. Excerpt of the crystal structure of **1** viewed along the *a* axis (left) and along the *b* axis (right).

WILEY-VCH

Table S5. Fractional atomic coordinates (×10[4]) and equivalent isotropic displacement parameters (Å[2]×10[3]) for **1**. U(eq) is defined as 1/3 of the trace of the orthogonalized U_{IJ} tensor.

Atom	*x*	*y*	*z*	U_{eq}	Atom	*x*	*y*	*z*	U_{eq}
S1	1077.2(14)	4504.1(15)	5633.8(8)	35.5(5)	S7	7773.1(15)	11174.4(16)	9120.9(9)	42.4(5)
S2	2244.3(14)	2321.6(16)	5555.6(8)	38.6(5)	S8	8845.8(14)	8993.5(16)	9348.0(8)	40.5(5)
S3	-170.6(14)	2384.2(16)	4552.8(8)	37.3(5)	S9	6567.7(14)	8921.7(15)	8200.0(8)	35.3(5)
S4	-58.2(14)	982.0(16)	5638.7(9)	40.4(5)	S10	6416.7(14)	7950.1(16)	9431.5(8)	41.1(5)
S5	1205.7(13)	3089.3(15)	6727.0(8)	34.1(5)	S11	7642.6(14)	10202.2(16)	10366.2(8)	38.3(5)
S6	-1206.0(13)	3171.8(15)	5738.6(8)	34.4(5)	S12	5375.1(13)	10142.2(15)	9226.3(8)	36.3(5)
Si1	290.4(15)	3962.9(16)	6204.2(9)	32.3(5)	Si5	6874.2(15)	10917.4(17)	9698.0(9)	34.4(5)
Si2	1383.4(15)	1850.5(17)	6106.6(9)	33.6(5)	Si6	7855.2(15)	8828.8(17)	9900.4(9)	34.6(5)
Si3	-913.8(15)	1953.8(17)	5169.8(9)	32.2(5)	Si7	5692.1(15)	8762.6(17)	8817.1(9)	33.9(5)
Si4	1276.5(14)	3199.8(16)	5079.4(9)	32.1(5)	Si8	8014.8(15)	9765.2(17)	8730.0(9)	33.7(5)
C1	36(5)	5065(6)	6684(3)	35.0(18)	C41	6699(6)	12148(6)	10047(3)	37.5(19)
C2	348(5)	5988(6)	6595(3)	40(2)	C42	5717(6)	12428(7)	9937(4)	48(2)
C3	111(6)	6869(6)	6908(4)	48(2)	C43	5529(6)	13378(6)	10170(4)	49(2)
C4	-476(6)	6798(7)	7285(4)	52(2)	C44	6337(7)	14048(7)	10528(4)	56(2)
C5	-840(5)	5888(7)	7390(3)	45(2)	C45	7360(6)	13822(6)	10665(4)	46(2)
C6	-1488(6)	5833(8)	7763(4)	54(2)	C46	8184(7)	14503(7)	11036(4)	57(2)
C7	-1858(6)	4941(8)	7852(4)	58(3)	C47	9172(7)	14291(7)	11170(4)	59(3)
C8	-1577(6)	4092(7)	7581(3)	50(2)	C48	9378(6)	13373(7)	10935(4)	49(2)
C9	-943(5)	4125(7)	7215(3)	45(2)	C49	8587(6)	12668(6)	10563(3)	46(2)
C10	-562(5)	5016(6)	7095(3)	38.2(19)	C50	7552(6)	12872(7)	10426(3)	43(2)
C11	2102(6)	1012(6)	6545(3)	39.1(19)	C51	8499(5)	8138(6)	10447(3)	37.5(19)
C12	1606(6)	82(6)	6519(4)	48(2)	C52	9485(6)	7865(6)	10443(4)	52(2)
C13	2065(7)	-584(6)	6861(4)	47(2)	C53	10013(6)	7350(7)	10844(4)	57(3)
C14	3034(7)	-312(7)	7215(4)	57(3)	C54	9596(6)	7087(6)	11262(4)	56(2)
C15	3611(7)	601(7)	7264(4)	49(2)	C55	8620(6)	7344(6)	11304(3)	45(2)
C16	4623(7)	877(8)	7622(4)	58(3)	C56	8177(7)	7086(7)	11733(4)	57(2)
C17	5193(7)	1748(8)	7666(4)	66(3)	C57	7256(7)	7339(7)	11785(4)	61(3)
C18	4749(6)	2409(8)	7322(4)	61(3)	C58	6727(7)	7866(7)	11391(4)	59(3)
C19	3762(6)	2178(6)	6953(3)	45(2)	C59	7107(6)	8136(6)	10959(3)	45(2)
C20	3163(6)	1281(7)	6917(3)	46(2)	C60	8074(5)	7877(6)	10895(3)	40(2)
C21	-2214(5)	1259(6)	4777(3)	35.7(19)	C61	4422(5)	8008(6)	8427(3)	38(2)
C22	-2495(6)	378(6)	4942(4)	45(2)	C62	3535(5)	8388(6)	8516(3)	39.2(19)
C23	-3482(6)	-208(6)	4661(4)	50(2)	C63	2531(5)	7806(6)	8268(3)	42(2)
C24	-4153(6)	86(6)	4213(4)	45(2)	C64	2427(6)	6908(7)	7959(3)	48(2)
C25	-3918(5)	957(7)	4029(3)	44(2)	C65	3283(6)	6475(6)	7833(3)	41(2)
C26	-4609(6)	1251(7)	3572(4)	54(2)	C66	3175(6)	5536(7)	7503(4)	51(2)
C27	-4379(6)	2119(8)	3396(4)	59(3)	C67	3993(7)	5128(7)	7383(4)	58(3)
C28	-3449(6)	2735(6)	3685(3)	47(2)	C68	4999(7)	5674(6)	7613(4)	53(2)
C29	-2740(5)	2476(6)	4149(3)	39.7(19)	C69	5151(6)	6581(6)	7949(4)	48(2)
C30	-2945(6)	1574(6)	4326(3)	38.5(19)	C70	4295(6)	7039(6)	8075(3)	39(2)
C31	1961(5)	3615(6)	4563(3)	31.8(18)	C71	8874(5)	10000(6)	8260(3)	38(2)
C32	2209(5)	4612(6)	4597(3)	37.5(19)	C72	9856(6)	9724(6)	8392(3)	45(2)
C33	2715(5)	4976(6)	4220(3)	42(2)	C73	10581(6)	9976(6)	8089(4)	48(2)
C34	2954(5)	4331(6)	3789(3)	38.6(19)	C74	10341(6)	10484(6)	7662(4)	50(2)
C35	2716(5)	3319(7)	3733(3)	40(2)	C75	9343(6)	10785(6)	7492(3)	42(2)
C36	2961(5)	2666(7)	3287(3)	44(2)	C76	9060(7)	11288(7)	7033(4)	54(2)
C37	2713(6)	1663(7)	3224(3)	52(2)	C77	8106(7)	11555(7)	6864(4)	60(3)
C38	2228(5)	1277(7)	3605(3)	46(2)	C78	7355(7)	11295(7)	7150(4)	58(3)
C39	1984(5)	1904(6)	4043(3)	41(2)	C79	7605(6)	10829(6)	7601(3)	47(2)
C40	2216(5)	2942(6)	4116(3)	35.2(18)	C80	8599(6)	10532(6)	7797(3)	40(2)
C81	5248(5)	5982(6)	4275(3)	37.0(19)					
C82	5338(5)	6376(6)	4854(3)	38.0(19)					
C83	4917(5)	7212(6)	5014(3)	46(2)					
C84	4397(6)	7661(6)	4586(4)	50(2)					
C85	4285(6)	7285(7)	4001(4)	56(2)					
C86	4722(6)	6445(6)	3851(3)	47(2)					
C87	5711(5)	5052(6)	4099(3)	43(2)					

Table S6. Selected bond lengths in **1** [Å].

S1−Si1	2.128(3)	S6−Si3	2.122(3)	S10−Si6	2.125(3)
S1−Si4	2.142(3)	Si1−C1	1.869(8)	S10−Si7	2.111(3)
S2−Si2	2.116(3)	Si2−C11	1.876(8)	S11−Si5	2.131(3)
S2−Si4	2.125(3)	Si3−C21	1.854(7)	S11−Si6	2.126(3)
S3−Si3	2.126(3)	Si4−C31	1.861(7)	S12−Si5	2.134(3)
S3−Si4	2.133(3)	S7−Si5	2.115(3)	S12−Si7	2.133(3)
S4−Si2	2.121(3)	S7−Si8	2.115(3)	Si5−C41	1.837(8)
S4−Si3	2.139(3)	S8−Si6	2.135(3)	Si6−C51	1.847(8)
S5−Si1	2.142(3)	S8−Si8	2.131(3)	Si7−C61	1.853(7)
S5−Si2	2.153(3)	S9−Si7	2.146(3)	Si8−C71	1.858(7)
S6−Si1	2.136(3)	S9−Si8	2.149(3)		

Table S7. Selected bond angles in **1** [°].

Si1−S1−Si4	104.12(12)	S6−Si3−S4	112.23(12)	C41−Si5−S11	108.2(3)
Si2−S2−Si4	103.67(11)	C21−Si3−S3	109.4(3)	C41−Si5−S12	107.6(3)
Si3−S3−Si4	104.13(11)	C21−Si3−S4	106.9(3)	S10−Si6−S8	111.74(12)
Si2−S4−Si3	104.47(13)	C21−Si3−S6	104.6(3)	S10−Si6−S11	111.70(12)
Si1−S5−Si2	104.76(11)	S2−Si4−S1	112.15(12)	S11−Si6−S8	112.71(13)
Si3−S6−Si1	104.90(11)	S2−Si4−S3	112.72(13)	C51−Si6−S8	106.8(3)
S1−Si1−S5	111.37(11)	S3−Si4−S1	111.96(12)	C51−Si6−S10	106.0(3)
S1−Si1−S6	111.90(12)	C31−Si4−S1	106.7(3)	C51−Si6−S11	107.4(3)
S6−Si1−S5	111.32(13)	C31−Si4−S2	106.5(2)	S10−Si7−S9	110.89(12)
C1−Si1−S1	106.7(3)	C31−Si4−S3	106.3(2)	S10−Si7−S12	112.39(12)
C1−Si1−S5	110.1(2)	Si8−S7−Si5	105.64(13)	S12−Si7−S9	112.47(12)
C1−Si1−S6	105.1(2)	Si8−S8−Si6	103.42(11)	C61−Si7−S9	109.1(2)
S2−Si2−S4	113.28(12)	Si7−S9−Si8	103.78(11)	C61−Si7−S10	104.8(3)
S2−Si2−S5	110.55(13)	Si7−S10−Si6	105.20(13)	C61−Si7−S12	106.8(3)
S4−Si2−S5	112.06(12)	Si6−S11−Si5	104.12(12)	S7−Si8−S8	113.05(12)
C11−Si2−S2	109.8(2)	Si7−S12−Si5	103.72(12)	S7−Si8−S9	110.31(12)
C11−Si2−S4	104.1(3)	S7−Si5−S11	112.55(12)	S8−Si8−S9	112.25(13)
C11−Si2−S5	106.6(3)	S7−Si5−S12	110.98(12)	C71−Si8−S7	105.4(3)
S3−Si3−S4	110.38(12)	S11−Si5−S12	112.00(13)	C71−Si8−S8	105.6(3)
S6−Si3−S3	112.93(13)	C41−Si5−S7	105.1(3)	C71−Si8−S9	109.8(2)

WILEY-VCH

Table S8. Crystallographic data and refinement results of **3** and **4**.

Compound	**3**·CH_2Cl_2	**4**
Empirical Formula	$C_{48}H_{40}Au_2P_2S_4Si_2$, CH_2Cl_2	$C_{56}H_{44}Au_2P_2S_4Si_2$
Formula weight /g·mol^{-1}	1342.02	1357.20
Crystal color and shape	colorless plate	colorless plate
Crystal size /mm³	0.279 x 0.258 x 0.0.168	0.594 x 0.204 x 0.102
Crystal system	monoclinic	triclinic
Space group [Flack parameter]	*C*2/*c*	$P\bar{1}$
a /Å	17.0944(6)	9.1114(7)
b / Å	14.7258(6)	9.2410(8)
c / Å	19.7490(7)	15.7564(13)
α /°	90	106.597(7)
β /°	108.047(3)	91.667(7)
γ /°	90	101.759(7)
V / Å³	4726.8(3)	1239.31(18)
Z	4	1
ρ_{calcd} / g·cm^{-3}	1.886	1.819
$\mu_{(Mo\ K\alpha)}$ / mm^{-1}	6.644	6.232
Absorption correction type	numerical	numerical
Min./max. transmission	0.2660 / 0.8204	0.0548 / 0.4320
2*θ* range / deg	3.732 – 58.500	4.586 – 51.998
No. of measured reflections	26951	13736
R(int)	0.0525	0.0791
Independent Reflections	6411	4883
Independent Reflections (I>2σ(I))	4872	3950
No. of parameters	285	334
R_1 (I>2σ(I)) / wR_2 (all data)	0.0223 / 0.0486	0.0473 / 0.1125
S (all data)	0.867	0.963
Max. peak / hole / e$^-$/Å³	1.51 / −1.48	3.01 / −2.26
CCDC number	2015923	2015924

WILEY-VCH

Crystal Structure of 3

The highest peak of residual electron density on the difference Fourier map (1.51 $e^{-}/Å^{3}$) is found 0.900 Å from Au1. Excerpts of the crystal structure of **3** are shown in Figure S10.

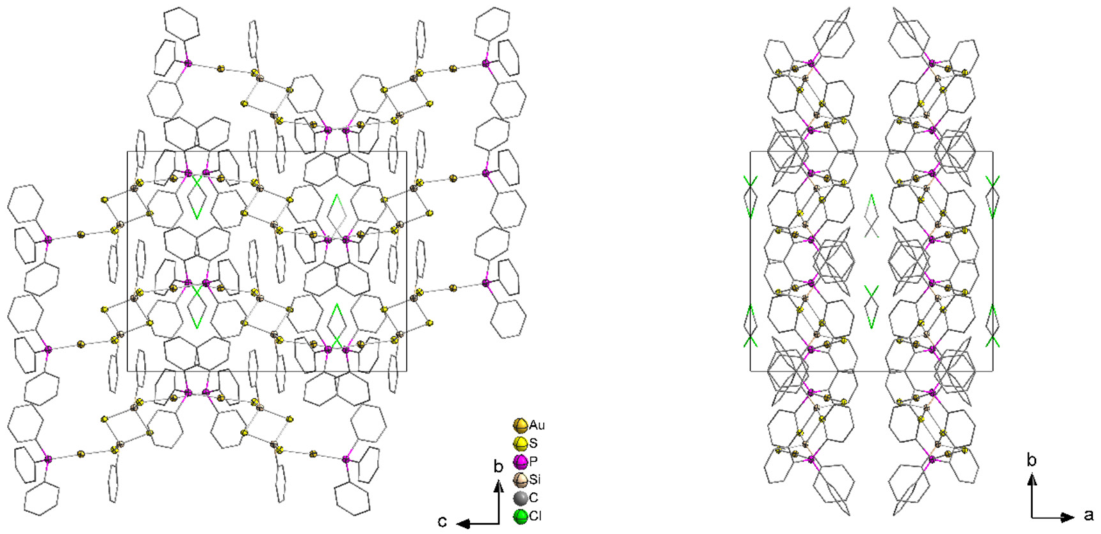

Figure S10. Excerpt of the crystal structure of **3** viewed along the *a* axis (left) and along the *c* axis (right).

WILEY

Table S9. Fractional atomic coordinates (×10[4]) and equivalent isotropic displacement parameters (Å^2×10^3) for **3**. U(eq) is defined as 1/3 of the trace of the orthogonalized U_{IJ} tensor.

Atom	*x*	*y*	*z*	U(eq)	Atom	*x*	*y*	*z*	U(eq)
Au1	8155.1(2)	6221.3(2)	3347.9(2)	19.92(4)	C12	6556(2)	4685(2)	2581.2(19)	33.4(8)
S1	7044.9(5)	7807.7(5)	4189.5(4)	20.65(15)	C13	6829(2)	6903(2)	1730.7(17)	24.3(6)
S2	8844.4(4)	6410.9(5)	4547.0(4)	21.18(15)	C14	6190(2)	6756(2)	1107(2)	31.2(7)
P1	7493.3(5)	5985.3(5)	2184.7(4)	21.23(16)	C15	5731(2)	7470(2)	739.6(19)	30.8(7)
Si1	7737.1(5)	6676.0(5)	4752.4(5)	18.57(16)	C16	5906(2)	8343(3)	986(2)	34.2(8)
C1	7062.0(19)	5648(2)	4602.1(17)	21.7(6)	C17	6526(3)	8498(3)	1608(2)	49.5(11)
C2	6207(2)	5732(2)	4441.3(19)	27.6(7)	C18	6985(2)	7785(2)	1987(2)	39.4(9)
C3	5707(2)	4977(2)	4340(2)	34.4(8)	C19	8222.2(19)	5835(2)	1690.8(17)	22.4(6)
C4	6054(2)	4124(2)	4402(2)	33.2(8)	C20	8437(2)	6552(2)	1334.0(19)	29.6(7)
C5	6891(2)	4022(2)	4566(2)	33.7(8)	C21	9036(2)	6446(2)	1006(2)	34.7(8)
C6	7393(2)	4777(2)	4658.8(18)	27.3(7)	C22	9424(2)	5627(2)	1025(2)	32.4(8)
C7	6864.6(19)	4967(2)	2040.6(18)	23.9(6)	C23	9213(2)	4907(2)	1372(2)	34.9(8)
C8	6688.1(19)	4467(2)	1416.7(18)	24.2(6)	C24	8625(2)	5006(2)	1718(2)	31.5(7)
C9	6201.8(19)	3692(2)	1330.7(18)	27.8(7)	Cl2	5000	8058.6(11)	2500	89.3(8)
C10	5893(2)	3420(2)	1863(2)	31.3(7)	Cl1	5263.7(13)	6134.1(13)	2660.5(18)	67.6(8)
C11	6063(2)	3916(3)	2486(2)	37.8(9)	C25	4725(6)	7033(5)	2168(5)	45(2)

Table S10. Selected bond lengths [Å] and angles [°] in **3**.

Au1−S2	2.3120(8)	P1−Au1−S2	178.03(3)	S1−Si1−Si1[1]	48.77(3)
Au1−P1	2.2510(8)	Si1−S1−Si1[1]	82.48(4)	S1[1]−Si1−Si1[1]	48.75(3)
S1−Si1[1]	2.1449(11)	Si1−S2−Au1	90.95(4)	S2−Si1−S1[1]	111.13(5)
S1−Si1	2.1444(11)	C7−P1−Au1	111.93(11)	S2−Si1−S1	115.57(5)
S2−Si1	2.0918(11)	C7−P1−C19	106.61(14)	S2−Si1−Si1[1]	126.93(5)
P1−C7	1.815(3)	C13−P1−Au1	115.43(11)	C1−Si1−S1	109.41(10)
P1−C13	1.813(3)	C13−P1−C7	106.76(15)	C1−Si1−S1[1]	110.72(11)
P1−C19	1.818(3)	C13−P1−C19	104.70(14)	C1−Si1−S2	111.67(10)
Si1−S1[1]	2.1449(11)	C19−P1−Au1	110.79(10)	C1−Si1−Si1[1]	121.36(11)
Si1−Si1[1]	2.8274(16)	S1−Si1−S1[1]	97.52(4)		
Si1−C1	1.870(3)				

[1]: 3/2-X, 3/2-Y, 1-Z

WILEY-VCH

Crystal Structure of 4

The highest peak of residual electron density on the difference Fourier map (3.01 e^{-}/Å^{3}) is found 0.89 Å from Au1. Excerpts of the crystal structure of **4** are shown in Figure S11.

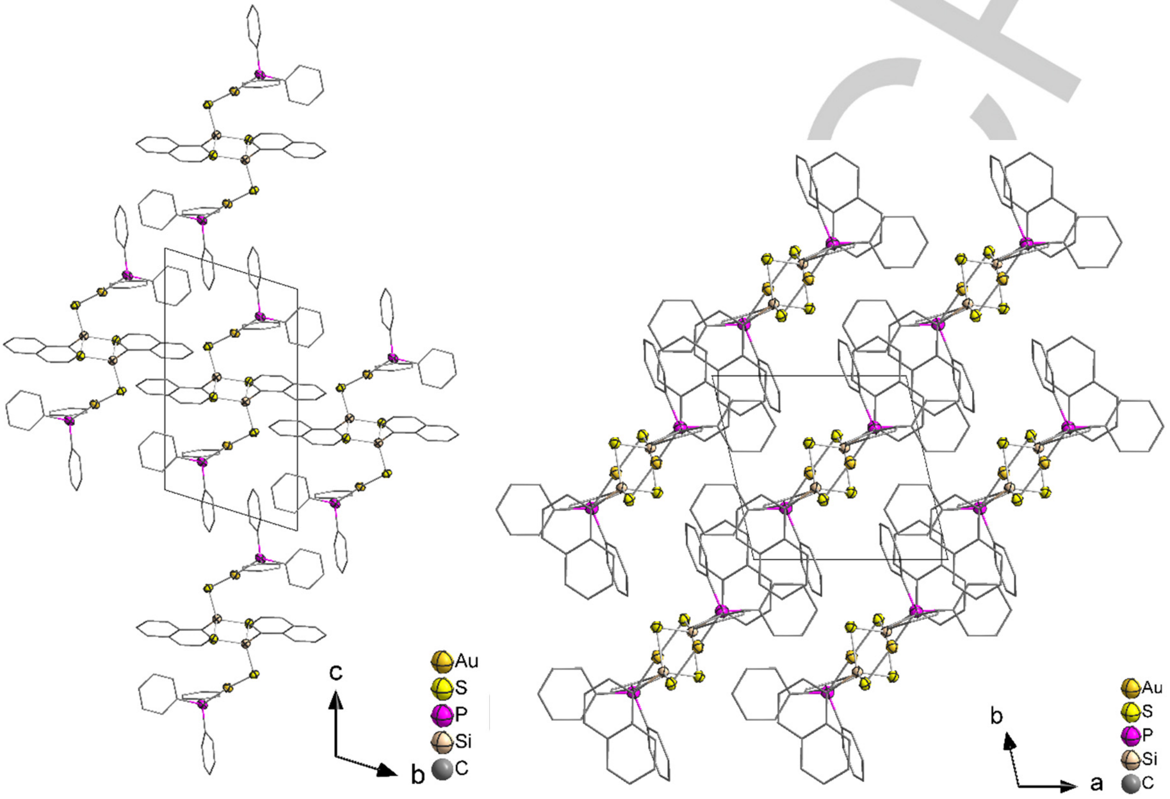

Figure S11. Excerpt of the crystal structure of **4** viewed along the *a* axis (left) and along the *c* axis (right).

WILEY-VCH

Table S11. Fractional atomic coordinates (×10^4) and equivalent isotropic displacement parameters (Å^2×10^3) for **4**. U(eq) is defined as 1/3 of the trace of the orthogonalized U_{IJ} tensor.

Atom	*x*	*y*	*z*	U(eq)	Atom	*x*	*y*	*z*	U(eq)
Au1	6103.2(3)	5277.9(3)	7370.2(2)	34.67(12)	C15	7340(20)	11687(17)	9323(9)	117(6)
S2	4281(2)	3274(2)	6481.9(12)	32.3(4)	C16	7569(18)	10204(15)	9283(7)	91(5)
S1	4202(2)	6343(2)	5547.3(12)	31.5(4)	C17	9696(9)	7274(9)	8178(5)	34.9(16)
P1	7755(2)	7194(2)	8377.7(13)	37.5(4)	C18	10765(10)	8668(10)	8388(5)	42.3(18)
Si1	4022(2)	3918(2)	5324.2(13)	27.8(4)	C19	12235(10)	8656(13)	8240(6)	53(2)
C1	2201(8)	2767(8)	4681(4)	28.5(14)	C20	12675(10)	7270(13)	7877(6)	52(2)
C2	1154(8)	3534(9)	4468(5)	32.4(15)	C21	11620(12)	5904(12)	7661(6)	53(2)
C3	-268(9)	2730(10)	4030(5)	38.9(18)	C22	10151(10)	5896(10)	7819(6)	43.2(19)
C4	-647(9)	1164(10)	3790(5)	38.7(17)	C23	7656(10)	6976(10)	9472(5)	46.1(19)
C5	395(9)	300(10)	3970(5)	36.4(16)	C24A	8930(30)	7226(18)	10098(12)	41(4)
C6	8(9)	-1330(10)	3743(5)	40.6(18)	C24B	8540(20)	6380(20)	9843(14)	61(5)
C7	997(10)	-2138(9)	3941(5)	40.9(18)	C25A	8810(40)	7030(20)	10914(15)	53(6)
C8	2433(10)	-1347(9)	4344(5)	41.2(18)	C25B	8430(30)	6240(30)	10692(17)	69(6)
C9	2830(9)	215(9)	4584(5)	33.1(15)	C26	7378(15)	6636(13)	11169(7)	69(3)
C10	1828(8)	1114(8)	4414(5)	30.8(15)	C27A	6160(30)	6260(30)	10602(16)	80(6)
C11	7418(9)	9095(10)	8475(5)	42.1(19)	C27B	6390(30)	7390(30)	10817(14)	66(5)
C12	6977(11)	9402(10)	7702(5)	47(2)	C28A	6250(30)	6410(30)	9737(13)	61(5)
C13	6732(13)	10840(12)	7748(7)	61(3)	C28B	6500(20)	7570(30)	9987(13)	58(5)
C14	6939(17)	11963(15)	8532(8)	83(4)					

Table S12. Selected bond lengths [Å] and angles [°] in **4**.

Au1−S2	2.297(2)	P1−Au1−S2	173.35(7)	S2−Si1−S1[1]	115.64(10)
Au1−P1	2.256(2)	Si1−S2−Au1	103.57(9)	S2−Si1−Si1[1]	130.00(13)
S2−Si1	2.095(2)	Si1−S1−Si11	82.58(10)	S1−Si1−S1[1]	97.42(10)
S1−Si1[1]	2.159(3)	C11−P1−Au1	113.0(3)	S1[1]−Si1−Si1[1]	48.39(7)
S1−Si1	2.137(3)	C17−P1−Au1	114.7(3)	S1−Si1−Si1[1]	49.04(7)
P1−C11	1.810(8)	C17−P1−C11	106.9(4)	C1−Si1−S2	109.1(2)
P1−C17	1.796(8)	C23−P1−Au1	111.6(3)	C1−Si1−S1	111.2(2)
P1−C23	1.795(8)	C23−P1−C11	105.8(4)	C1−Si1−S1[1]	108.4(2)
Si1−S1[1]	2.159(3)	C23−P1−C17	104.2(4)	C1−Si1−Si1[1]	120.9(2)
Si1−Si1[1]	2.835(4)	S2−Si1−S1	114.55(12)		
Si1−C1	1.865(8)				

[1]: 1-X, 1-Y, 1-Z

WILEY-VCH

5 Cartesian Coordinates of the Calculated Structures in the Gas Phase

Minimum structures of the cluster monomers calculated at BP86-D3/cc-pVDZ level of theory are given in Tables S13−S16

Table S13. Cartesian coordinates of [$(PhSi)_4S_6$].

S	2.55433628	-0.29872054	0.13015851
Si	1.02848834	-1.39222039	1.20513182
C	1.87458073	-2.65566202	2.30503137
C	3.27748826	-2.81000704	2.32652876
C	1.07614135	-3.47934861	3.13288149
S	0.29122564	2.51271157	0.13463471
Si	-1.10546566	1.24428128	1.22078195
C	-2.13349127	2.42413065	2.25716302
C	-2.13627669	2.37836850	3.66795738
C	-2.92308803	3.39439018	1.59648709
S	-2.51615927	0.25838467	-0.10973734
Si	-1.24154206	-1.04235823	-1.29524466
C	-2.38107572	-1.90987744	-2.50854879
C	-3.28402783	-1.13320898	-3.27165921
C	-2.34730087	-3.30879851	-2.69558833
S	0.14743511	0.08221688	-2.54310261
Si	1.39172284	1.09308344	-1.08466000
C	2.67791997	2.08560100	-2.02518957
C	2.99088960	1.79274407	-3.37099486
C	3.37637344	3.12256116	-1.36443164
S	-0.25947539	-2.56424631	-0.10375019
S	-0.11837439	-0.13865969	2.56808873
C	4.36600521	3.85355270	-2.03985055
C	3.98100647	2.52586463	-4.04483800
C	4.66849923	3.55591772	-3.38026029
C	-3.20238452	-3.92086712	-3.62653604
C	-4.13707931	-1.74676725	-4.20174611
C	-4.09688242	-3.14106809	-4.37932633
C	-3.70012600	4.29949355	2.33538858
C	-2.91496787	3.28509964	4.40564425
C	-3.69652328	4.24508353	3.74053617
C	1.67272043	-4.43831119	3.96564398
C	3.87240781	-3.77043827	3.16103478
C	3.07129790	-4.58419108	3.98004485
H	3.14217208	3.35996926	-0.31465352
H	2.45757374	0.98503689	-3.89632087
H	4.90301293	4.66020852	-1.51813905
H	4.21631881	2.29176566	-5.09415098
H	5.44389755	4.13046477	-3.90967448
H	-1.64747628	-3.92564486	-2.11049316
H	-3.31981027	-0.04080870	-3.13448455
H	-3.16987430	-5.01228177	-3.76425559
H	-4.83730914	-1.13468986	-4.79021089
H	-4.76690434	-3.62207168	-5.10823562
H	-2.92886621	3.44025915	0.49587195
H	-1.52548137	1.62946273	4.19580273
H	-4.31157748	5.05082841	1.81301509
H	-2.91101281	3.24139316	5.50531801
H	-4.30640601	4.95506041	4.31982510
H	-0.01978720	-3.36806390	3.12456304
H	3.91096408	-2.17602896	1.68677769
H	1.04403653	-5.07491240	4.60653699
H	4.96706875	-3.88353793	3.17129792
H	3.53885591	-5.33649932	4.63351629

WILEY-VCH

Table S14. Cartesian coordinates of [$(NpSi)_4S_6$].

S	2.56657572	-0.34762781	0.37368617
Si	0.90766135	-1.55677507	1.08867223
C	1.66448839	-2.97585560	2.07120157
C	2.57664550	-3.91401085	1.46163269
C	1.33199053	-3.11680137	3.42028426
S	0.35194313	2.50220679	0.54340046
Si	-1.18854865	1.14043693	1.24851806
C	-2.31661478	2.14674098	2.37400511
C	-2.45688481	1.76220520	3.70924811
C	-3.03430157	3.30062954	1.88689282
S	-2.43571355	0.38959040	-0.36577696
Si	-1.04754822	-0.74976473	-1.59030510
C	-2.04711781	-1.44157724	-3.03054235
C	-1.68928807	-1.08750305	-4.33324155
C	-3.16880880	-2.32454235	-2.81607371
S	0.50068757	0.50631836	-2.46113120
Si	1.59012843	1.24979626	-0.73077626
C	2.96196071	2.35585050	-1.39899609
C	3.07798798	2.52935135	-2.77989667
C	3.88979449	3.02380644	-0.51769739
S	-0.22018625	-2.46057182	-0.53486161
S	-0.37154730	-0.46483116	2.46889043
C	4.91976296	3.85928321	-1.09601576
C	4.09135530	3.35079344	-3.34121687
C	4.99267544	4.00143444	-2.51359576
C	-3.89421808	-2.81946069	-3.96591947
C	-2.40536070	-1.57703840	-5.45768652
C	-3.48566747	-2.42563984	-5.27474799
C	-3.88428112	4.02908768	2.80353164
C	-3.29261575	2.48181667	4.60397871
C	-3.99113822	3.59234280	4.15736683
C	1.87213224	-4.16533910	4.21123791
C	3.12357784	-4.97971877	2.27309650
C	2.75009940	-5.07685529	3.64646049
H	2.37400269	2.02323921	-3.45814597
H	4.15359545	3.46502170	-4.43362863
H	5.78239784	4.64083978	-2.93880907
C	-3.60319110	-2.73800965	-1.51952521
H	-0.83506300	-0.41462914	-4.50449525

C	-5.00584389	-3.69302552	-3.77080449
H	-2.09588210	-1.27756738	-6.47002233
H	-4.04787983	-2.81082386	-6.14011456
C	-2.94876919	3.76419708	0.53837771
H	-1.91246389	0.88353835	4.08785058
C	-4.60085739	5.17196547	2.33744146
H	-3.37971352	2.15084994	5.64952112
H	-4.64210233	4.15737526	4.84314701
H	0.63696814	-2.40525920	3.89189843
H	1.58848215	-4.24642844	5.27107335
H	3.17489282	-5.89363966	4.25121118
C	-4.49288250	5.59222900	1.02012832
C	-3.65891134	4.87991793	0.11636900
H	-5.24339314	5.71482480	3.04840622
H	-5.05035410	6.47494404	0.67256231
H	-3.57347779	5.21385354	-0.92839199
H	-2.31045949	3.23044170	-0.17985644
C	2.97039026	-3.84484880	0.09018479
C	4.02714974	-5.91519834	1.68546635
C	3.85160089	-4.77087051	-0.45126285
C	4.38659100	-5.81591574	0.34959392
H	2.57037707	-3.04582604	-0.54996319
H	4.13696687	-4.69273156	-1.51088139
H	5.08417389	-6.54326817	-0.09210480
H	4.43418120	-6.72017282	2.31741794
C	-4.68880013	-3.58912268	-1.36365055
C	-5.39853770	-4.07268379	-2.49595013
H	-3.07074498	-2.37768251	-0.62798426
H	-5.00067107	-3.89067473	-0.35263995
H	-6.25729424	-4.74673812	-2.35829112
H	-5.54688426	-4.06042720	-4.65698220
C	5.84484029	4.52659418	-0.23823134
C	3.84404161	2.90237518	0.90493103
C	5.76912315	4.38465526	1.13935160
C	4.75906900	3.56497367	1.71173325
H	6.62260834	5.15865487	-0.69496128
H	6.48814047	4.90427810	1.79032401
H	3.07223744	2.27408627	1.37170989
H	4.69955088	3.45275066	2.8045247978

Table S15. Cartesian coordinates of $[(PhSn)_4S_6]$.

S	2.79824933	-0.29409155	0.13516696
Sn	1.10707370	-1.54810426	1.36916664
C	2.17773885	-2.93736690	2.60666886
C	3.39026460	-3.48989652	2.14848393
C	1.66397589	-3.29677850	3.86817186
S	0.26781264	2.84855182	0.16691975
Sn	-1.29446256	1.39831154	1.36793585
C	-2.45515095	2.71245045	2.60649048
C	-2.93040293	2.26465885	3.85478732
C	-2.74022120	4.01940298	2.16385233
S	-2.83116286	0.27532662	-0.16369989
Sn	-1.39383416	-1.18299831	-1.50216660
C	-2.66276385	-2.23881557	-2.87459478
C	-2.97294770	-1.66577462	-4.12384074
C	-3.18620635	-3.49780797	-2.51974929
S	0.13955657	0.11608107	-2.90026263
Sn	1.50069047	1.26992620	-1.23309215
C	2.91733929	2.44582955	-2.33630528
C	3.31528244	2.03786257	-3.62473672
C	3.44946706	3.61872324	-1.76501826
S	-0.29440905	-2.87913123	-0.13609149
S	-0.20071645	-0.16201510	2.90072209
C	4.38239220	4.38294090	-2.48711586
C	4.24874470	2.80707693	-4.34092133
C	4.78165503	3.97691500	-3.77230132
C	-4.02233043	-4.18240400	-3.41874428
C	-3.80973116	-2.35606620	-5.01774820
C	-4.33397520	-3.61169873	-4.66494056
C	-3.50345465	4.87763566	2.97420776
C	-3.69302814	3.12842840	4.65993456
C	-3.97948712	4.43218521	4.21955208
C	2.36644325	-4.21234317	4.67084831
C	4.08735119	-4.40471266	2.95650561
C	3.57542051	-4.76573067	4.21488849
H	3.13631193	3.94340794	-0.76008846
H	2.89701211	1.12419925	-4.07615794
H	4.79794173	5.30035321	-2.04299591
H	4.55957727	2.49058498	-5.34829583
H	5.51221386	4.57738646	-4.33541310
H	-2.94100400	-3.95122972	-1.54623598
H	-2.56088022	-0.68416692	-4.40683795
H	-4.43066046	-5.16687619	-3.14368254
H	-4.05185727	-1.91037152	-5.99464646
H	-4.98879010	-4.15007450	-5.36690186
H	-2.36498330	4.37497586	1.19116494
H	-2.70442534	1.24540653	4.20627822
H	-3.72556540	5.89948759	2.63060695
H	-4.06352127	2.78061270	5.63632470
H	-4.57649265	5.10653454	4.85216624
H	0.71933031	-2.86208078	4.23179055
H	3.79849772	-3.20616134	1.16556607
H	1.96714816	-4.49287879	5.65748700
H	5.03566728	-4.83575510	2.60089718
H	4.12362077	-5.48200883	4.84559413

WILEY-VCH

Table S16. Cartesian coordinates of [(NpSn)$_4$S$_6$].

S	2.87258975	-0.31448999	0.33272804
Sn	1.07560590	-1.67923117	1.27168866
C	1.99008730	-3.22904974	2.45146145
C	2.89410352	-4.16547207	1.85025781
C	1.68208439	-3.29797447	3.80645240
S	0.34073313	2.81368655	0.50349220
Sn	-1.31717895	1.27755456	1.43393202
C	-2.57452609	2.41291848	2.76064547
C	-2.63602830	2.03552410	4.09834249
C	-3.32779938	3.53094547	2.27255797
S	-2.74384535	0.36220830	-0.32671008
Sn	-1.20881286	-0.91267766	-1.73802748
C	-2.36244690	-1.75570727	-3.34708679
C	-2.02711124	-1.41106930	-4.65261676
C	-3.44986947	-2.64949529	-3.07457823
S	0.45610905	0.49324727	-2.84704549
Sn	1.70671025	1.40560484	-0.95423280
C	3.20535755	2.66287422	-1.85077201
C	3.23924890	2.76609596	-3.23779744
C	4.14591458	3.37211714	-1.03348844
S	-0.21386574	-2.76801287	-0.49627427
S	-0.32679788	-0.44978587	2.85303484
C	5.13546272	4.20036572	-1.68909315
C	4.21516805	3.58318272	-3.87437140
C	5.14036225	4.28239091	-3.11394631
C	-4.19275493	-3.18630683	-4.19482196
C	-2.76267887	-1.94350812	-5.74837523
C	-3.82046910	-2.81083684	-5.52047897
C	-4.15667279	4.25985199	3.20885500
C	-3.45357968	2.75768765	5.01230934
C	-4.19526843	3.84426011	4.57368304
C	2.26313896	-4.30632439	4.62551167
C	3.48054947	-5.18775583	2.69049292
C	3.14168917	-5.22810179	4.07630986
H	2.51177377	2.21651285	-3.85641005
H	4.22897769	3.65446608	-4.97227402
H	5.89799414	4.91574102	-3.60172060
C	-3.83503812	-3.03503029	-1.75543582
H	-1.18963907	-0.72308216	-4.85075034
C	-5.27866850	-4.07745815	-3.94317905
H	-2.48515831	-1.66134328	-6.77503401
H	-4.39299114	-3.22561426	-6.36501555
C	-3.30036859	3.95957669	0.91141512
H	-2.05288983	1.17469564	4.46286114
C	-4.91366636	5.37571849	2.74142222
H	-3.49154069	2.44684354	6.06710571
H	-4.82948327	4.40657639	5.27698693
H	0.98594516	-2.57322479	4.25819913
H	2.00955600	-4.34765222	5.69539414
H	3.59409232	-6.01046669	4.70583383
C	-4.86262280	5.76424747	1.41045865
C	-4.04865890	5.04909592	0.48922598
H	-5.54148453	5.92429749	3.46109268
H	-5.45147976	6.62663649	1.06371348
H	-4.01104309	5.36055447	-0.56504416
H	-2.67534594	3.41765334	0.18389914
C	3.24330242	-4.13906694	0.46660916
C	4.38268114	-6.12879142	2.10971654
C	4.12400035	-5.06910258	-0.06688331
C	4.69942667	-6.07295946	0.76005788
H	2.80708255	-3.36860208	-0.18892137
H	4.37778626	-5.02850031	-1.13639029
H	5.39653153	-6.80499761	0.32547461
H	4.82399375	-6.90310652	2.75678769
C	-4.89623710	-3.90384258	-1.54509041
C	-5.62523071	-4.43037586	-2.64689317
H	-3.28158811	-2.63712047	-0.89002498
H	-5.17429639	-4.18628663	-0.51902213
H	-6.46485801	-5.11849049	-2.46738683
H	-5.83814909	-4.48085772	-4.80182520
C	6.08059031	4.91324152	-0.89208818
C	4.15443814	3.30141004	0.39203061
C	6.05940723	4.82046932	0.49209888
C	5.08745845	4.00743677	1.13781910
H	6.82991418	5.54079685	-1.39974029
H	6.79421643	5.37552997	1.09431258
H	3.40888580	2.67674293	0.90925672
H	5.07407505	3.93688279	2.23542947

WILEY-VCH

Minimum structures of the alternating (first) and stacking (second) cluster dimers calculated at BP86-D3/cc-pVDZ level of theory are given in Tables S17−S24.

Table S17. Cartesian coordinates of [$(PhSi)_4S_6]_2$ (alternating).

S	1.49970598	-2.07688909	0.08535839
Si	2.26418564	-1.00314978	-1.63830185
C	1.63277414	-1.94950293	-3.13507285
C	1.40656894	-3.34155544	-3.05529689
C	0.89245194	-4.04142830	-4.15851800
C	0.60005603	-3.35894122	-5.35219123
C	0.83331764	-1.97575921	-5.44400077
C	1.34793653	-1.27391858	-4.34234762
H	1.51246350	-0.18794718	-4.41854201
H	0.60864577	-1.43867502	-6.37828795
H	0.19006329	-3.90722957	-6.21427894
H	0.71459912	-5.12529871	-4.08443773
H	1.61713559	-3.88091195	-2.11874729
S	4.43514276	-1.03527933	-1.72548037
Si	5.00950386	0.08441428	0.05278908
C	6.89114776	0.08678208	0.03556018
C	7.60403350	1.12598530	-0.60394917
C	9.00670776	1.09541608	-0.66104341
C	9.71098043	0.02539194	-0.08212653
C	9.01153706	-1.01589306	0.55261124
C	7.60901718	-0.98664959	0.61054921
H	7.06563183	-1.80684575	1.10633869
H	9.56106305	-1.85529532	1.00562972
H	10.81084592	0.00233185	-0.12661156
H	9.55241881	1.91117603	-1.15956685
H	7.05718703	1.96500578	-1.06293059
S	4.40547773	-0.90173536	1.89553275
Si	2.23311171	-0.88635932	1.73953736
C	1.58188837	-1.71754223	3.29446132
C	1.31923402	-3.10520799	3.30837109
C	0.79086315	-3.71584230	4.45686713
C	0.52187040	-2.94765711	5.60303257
C	0.79226834	-1.56814844	5.60199182
C	1.32009570	-0.95518599	4.45437978
H	1.51385874	0.12870991	4.45612456
H	0.58660069	-0.96455031	6.49934940
H	0.10129675	-3.42604496	6.50091778
H	0.58366254	-4.79701337	4.45554193
H	1.51235365	-3.71157713	2.40986254
S	1.43168735	1.12815418	1.78237006
Si	2.20358366	1.98272357	-0.05402215
C	1.52791447	3.73543497	-0.12158288
C	1.27948372	4.35698517	-1.36541425
C	0.73417065	5.64976828	-1.41630456
C	0.43313893	6.33493452	-0.22627782
C	0.68862498	5.72839905	1.01587417
C	1.23417321	4.43585130	1.06925537
H	1.41674396	3.96243537	2.04637405
H	0.45756533	6.26358191	1.94968077
H	-0.00091330	7.34580999	-0.26711541
H	0.53899611	6.12348333	-2.39069013
H	1.49743026	3.82100721	-2.30219916
S	1.46766105	1.00438996	-1.84125819
S	4.37452113	2.16319738	-0.02252926
S	-4.40554116	0.90170798	-1.89556582
Si	-5.00956174	-0.08442104	-0.05281234
C	-6.89120513	-0.08683807	-0.03553761
C	-7.60399851	-1.12579346	0.60448207
C	-9.00666915	-1.09526265	0.66166568
C	-9.71103286	-0.02552624	0.08232681
C	-9.01168269	1.01551015	-0.55292003
C	-7.60916472	0.98630680	-0.61094462
H	-7.06585285	1.80631298	-1.10712830
H	-9.56127971	1.85468708	-1.00626969
H	-10.81089619	-0.00249691	0.12688119
H	-9.55230681	-1.91082712	1.16058922
H	-7.05707955	-1.96458560	1.06379507
S	-4.37456777	-2.16320223	0.02252200
Si	-2.20363334	-1.98272883	0.05402892
C	-1.52796025	-3.73543741	0.12161194
C	-1.23417693	-4.43585651	-1.06921435
C	-0.68863281	-5.72840493	-1.01581137
C	-0.43319832	-6.33494038	0.22635137
C	-0.73427358	-5.64977173	1.41636561
C	-1.27957717	-4.35698551	1.36545359
H	-1.49755379	-3.82100497	2.30222993
H	-0.53914086	-6.12348642	2.39075953
H	0.00084788	-7.34581778	0.26720690
H	-0.45753731	-6.26358922	-1.94960834
H	-1.41671285	-3.96244250	-2.04634037
S	-1.46771466	-1.00437122	1.84125298
Si	-2.26424596	1.00316392	1.63827759
C	-1.63284159	1.94952821	3.13504513
C	-1.40663494	3.34158015	3.05526698
C	-0.89252634	4.04145564	4.15849078
C	-0.60013871	3.35897197	5.35216784
C	-0.83340199	1.97579024	5.44397971
C	-1.34801443	1.27394767	4.34232490
H	-1.51254453	0.18797686	4.41852099
H	-0.60873706	1.43870880	6.37827036
H	-0.19015152	3.90726251	6.21425680
H	-0.71467288	5.12532583	4.08440926
H	-1.61719452	3.88093440	2.11871444
S	-4.43520171	1.03529092	1.72544689
S	-1.49977165	2.07689673	-0.08539192
Si	-2.23317179	0.88634222	-1.73955438
C	-1.58195418	1.71748252	-3.29450374
C	-1.31917245	3.10512384	-3.30843254
C	-0.79079758	3.71570413	-4.45695549
C	-0.52193819	2.94749025	-5.60313311
C	-0.79246839	1.56800763	-5.60207497
C	-1.32028966	0.95509709	-4.45443239
H	-1.51414729	-0.12878209	-4.45616177
H	-0.58690864	0.96438739	-6.49944229
H	-0.10136533	3.42583549	-6.50104136
H	-0.58348957	4.79685478	-4.45564380
H	-1.51219503	3.71151526	-2.40991827
S	-1.43173782	-1.12816776	-1.78236655

Table S18. Cartesian coordinates of [$(PhSi)_4S_6$] (stacking).

S	1.01715024	0.59843551	1.73410988
Si	2.62731818	-0.86493810	1.69681824
C	2.19502463	-2.16992271	2.98040083
C	1.31942625	-3.23316444	2.66172771
C	1.01213525	-4.21267611	3.61702285
C	1.58122167	-4.14620676	4.89975759
C	2.44786690	-3.09139859	5.23113689
C	2.74903023	-2.10479287	4.27874409
H	3.42751817	-1.27920432	4.54597763
H	2.89060968	-3.03374200	6.23734378
H	1.34023521	-4.91664250	5.64809071
H	0.31967764	-5.02753812	3.35939816
H	0.87336392	-3.29818327	1.65770450
S	4.51803325	-0.00584080	2.32417885
Si	4.89789958	1.49919507	0.79278237
C	6.53062639	2.28075614	1.30388835
C	6.56626586	3.50643568	2.00539615
C	7.79161429	4.05167233	2.42198020
C	8.99261543	3.37625343	2.14402665
C	8.96840899	2.15361919	1.45009529
C	7.74427342	1.60764955	1.03274375
H	7.73016187	0.64838711	0.49079865
H	9.90790122	1.62287490	1.23222758
H	9.95298146	3.80448402	2.47045130
H	7.80863154	5.00843089	2.96614313
H	5.62775571	4.03784063	2.23005006
S	3.41456858	3.07098061	0.69908866
Si	1.58268271	1.99236829	0.18453560
C	0.32370077	3.38077218	0.15927662
C	0.19123771	4.19736901	-0.98631444
C	-0.57898242	5.36865770	-0.93381533
C	-1.22041095	5.73668814	0.26112333
C	-1.11502899	4.91783955	1.39632470
C	-0.34484044	3.74510918	1.34862317
H	-0.25062916	3.11501767	2.24640195
H	-1.63519382	5.19359132	2.32556932
H	-1.82343251	6.65513507	0.30120702
H	-0.67798030	6.00024227	-1.82968799
H	0.70657263	3.92022241	-1.91951427
S	1.67346733	1.09618593	-1.79737621
Si	3.28841382	-0.33656000	-1.58098286
C	3.63365262	-1.09004070	-3.26528530
C	4.20313969	-2.37887555	-3.36501127
C	4.62702514	-2.87393361	-4.60815095
C	4.49073182	-2.08292770	-5.76284924
C	3.92416578	-0.80005131	-5.67365058
C	3.49675654	-0.30562489	-4.43080698
H	3.06072512	0.70345946	-4.36417002
H	3.81351256	-0.18052025	-6.57673973
H	4.82933895	-2.46848310	-6.73697716
H	5.06972632	-3.87951340	-4.67590971
H	4.32401535	-2.99568548	-2.46035363
S	2.75318497	-1.92787711	-0.20556473
S	5.21702393	0.57695778	-1.14371098
S	-3.42722681	-2.04316142	-2.33726542
Si	-4.86081446	-1.50095034	-0.81044898
C	-6.49016802	-2.34136073	-1.23069460
C	-6.52345266	-3.51418852	-2.01767075
C	-7.74281434	-4.15931957	-2.28066642
C	-8.94003018	-3.64040326	-1.75783566
C	-8.91790666	-2.47610340	-0.96994419
C	-7.69969734	-1.83002550	-0.70645182
H	-7.68707940	-0.91921274	-0.08651221
H	-9.85438051	-2.06878603	-0.55895759
H	-9.89573985	-4.14638904	-1.96480422
H	-7.75819740	-5.07143731	-2.89678384
H	-5.58764032	-3.92785900	-2.42614070
S	-4.37445275	-2.23875664	1.18297531
Si	-2.48881711	-1.25637715	1.61352720
C	-1.96558029	-1.85015189	3.31966236
C	-2.46729745	-3.06505071	3.83833479
C	-2.09791996	-3.49698634	5.12216206
C	-1.21527225	-2.72520073	5.89595236
C	-0.69771927	-1.52346899	5.38434123
C	-1.07242753	-1.08641785	4.10568341
H	-0.66596551	-0.14051953	3.71662689
H	0.00704771	-0.92527548	5.98041942
H	-0.92133659	-3.06619885	6.90043810
H	-2.50030183	-4.44195144	5.51830103
H	-3.15852142	-3.67597312	3.23641115
S	-2.66589118	0.91341950	1.77586557
Si	-3.30332154	1.46897579	-0.22257139
C	-3.69917641	3.30389672	-0.23870737
C	-3.63605485	4.02880040	-1.44828815
C	-4.10351636	5.35157312	-1.50838195
C	-4.63642203	5.96185099	-0.36021704
C	-4.69903210	5.24959923	0.85071254
C	-4.23534977	3.92615884	0.91050973
H	-4.29907925	3.36635638	1.85711192
H	-5.11530475	5.72606238	1.75152520
H	-5.00640799	6.99769082	-0.40900655
H	-4.05046000	5.90843786	-2.45638742
H	-3.22647027	3.55174913	-2.35244232
S	-5.23638040	0.63445180	-0.78183560
S	-1.73831234	1.09960170	-1.67901772
Si	-1.58922093	-1.07213935	-1.65726749
C	-0.37010553	-1.60461504	-2.97770553
C	-0.30668112	-0.91030491	-4.20680756
C	0.43437011	-1.43382823	-5.27670811
C	1.11530540	-2.65440551	-5.13117471
C	1.07822341	-3.33793985	-3.90574203
C	0.33717934	-2.81832725	-2.83261446
H	0.29650253	-3.36689405	-1.87905775
H	1.62913665	-4.28229388	-3.78391335
H	1.69591342	-3.06353194	-5.97039102
H	0.47966118	-0.88782088	-6.23121698
H	-0.85283854	0.03834924	-4.32917597
S	-0.92057651	-1.86941888	0.23511494

Table S19. Cartesian coordinates of [(NpSi)$_4$S$_6$]$_2$ (alternating).

S	-1.32424564	-1.07316990	-2.00078151
Si	-2.22050522	-2.25698887	-0.41807486
C	-1.42963569	-3.96133947	-0.56329180
C	-0.76164447	-4.48047463	0.54804986
C	-0.02830332	-5.69315576	0.47171388
C	0.05674420	-6.37319255	-0.73267454
C	-0.62438772	-5.89908544	-1.89379900
C	-0.56387070	-6.60701636	-3.13210008
C	-1.26330845	-6.16165164	-4.24501570
C	-2.05378108	-4.98254974	-4.15938314
C	-2.11826249	-4.25990735	-2.97519380
C	-1.40418927	-4.68278048	-1.81165979
H	-2.73427776	-3.35019701	-2.93087217
H	-2.61729676	-4.63489934	-5.03831367
H	-1.21275671	-6.72131329	-5.19138813
H	0.04288747	-7.52505692	-3.18295897
H	0.64794604	-7.29967880	-0.80946886
H	0.48392584	-6.07147344	1.36669649
H	-0.77773108	-3.94032349	1.50729100
S	-4.37249084	-2.51918649	-0.66795970
Si	-5.12907965	-0.48549303	-0.50216299
C	-7.00390483	-0.57650953	-0.71319091
C	-7.59554762	-1.82201770	-0.93453733
C	-8.99965515	-1.95696192	-1.10229902
C	-9.81386806	-0.83674510	-1.04825109
C	-9.26196667	0.46002880	-0.82539670
C	-10.09501350	1.61772482	-0.76920906
C	-9.55336795	2.87605243	-0.55234498
C	-8.14982997	3.02211043	-0.38257808
C	-7.31215878	1.91617330	-0.43180350
C	-7.83256607	0.60403682	-0.65327249
H	-6.22968391	2.05313353	-0.29673403
H	-7.72087998	4.02069857	-0.20984269
H	-10.20623447	3.76110509	-0.51098290
H	-11.18116040	1.49016553	-0.90213334
H	-10.90413235	-0.92992078	-1.17711960
H	-9.43198155	-2.95401266	-1.27477140
H	-6.96882281	-2.72612741	-0.98259943
S	-4.77789260	0.35085369	1.47895798
Si	-2.60638676	0.44089651	1.59925324
C	-2.15222007	1.15588336	3.28277684
C	-1.47512809	2.37659169	3.32700377
C	-0.99262580	2.91223691	4.54948730
C	-1.16614465	2.20524134	5.72860060
C	-1.86892120	0.96314630	5.74373416
C	-2.07531424	0.24040166	6.95748270
C	-2.79783747	-0.94449279	6.96983750
C	-3.34360508	-1.45373531	5.75938090
C	-3.14378026	-0.78551168	4.55843935
C	-2.39677977	0.43167600	4.50557064
H	-3.57354898	-1.19772830	3.63404461
H	-3.92683785	-2.38679601	5.77043167
H	-2.95510420	-1.48738881	7.91425981
H	-1.65783777	0.65103325	7.89057381
H	-0.76932908	2.59835689	6.67823133
H	-0.46612332	3.87618578	4.54578383
H	-1.28606354	2.94014546	2.40030824
S	-1.69887538	-1.53248412	1.54575116
S	-1.75437272	1.78817593	0.14225245
Si	-2.25546621	0.85904983	-1.75581830
C	-1.48949176	1.96844539	-3.07279571
C	-0.58803072	1.40344344	-3.97763810
C	0.12098508	2.20127166	-4.91310657
C	-0.05607321	3.57562925	-4.92182162

C	-0.98095899	4.20275209	-4.03399241
C	-1.18974951	5.61493626	-4.05342428
C	-2.12248871	6.21134751	-3.21647954
C	-2.88523778	5.41092652	-2.32205988
C	-2.69213771	4.03694665	-2.26445424
C	-1.73386054	3.38923097	-3.10375994
H	-3.29042459	3.43626581	-1.56429443
H	-3.63254648	5.88219826	-1.66603451
H	-2.27768686	7.30054216	-3.24722784
H	-0.60108721	6.22309515	-4.75852997
H	0.51119908	4.20672792	-5.62448340
H	0.82089871	1.72155096	-5.61059431
H	-0.39752292	0.31911163	-3.96705579
S	-4.40025059	0.78525689	-2.11518378
S	1.18221247	0.58578907	2.17822675
Si	2.06504308	1.86915830	0.66184443
C	1.29130775	3.56567404	0.93950570
C	0.54315386	4.13738601	-0.09155670
C	-0.13860087	5.37077334	0.08228664
C	-0.07239245	6.02958551	1.29979509
C	0.69744896	5.50191752	2.38000685
C	0.78837869	6.18602760	3.63021957
C	1.55591822	5.67727432	4.66928339
C	2.25570084	4.45187528	4.49757212
C	2.17538235	3.75435426	3.29971999
C	1.40191493	4.25014487	2.20498004
H	2.71739140	2.80403422	3.19297772
H	2.86111342	4.04619187	5.32175472
H	1.62135634	6.21812013	5.62555243
H	0.24060957	7.13415313	3.75094056
H	-0.60952337	6.97953246	1.45177017
H	-0.72660921	5.77871252	-0.75159785
H	0.45611503	3.62676210	-1.06275513
S	4.21510493	2.12646838	0.90226311
Si	4.99291133	0.11338979	0.62793795
C	6.86796082	0.23222892	0.82033926
C	7.48798551	-0.50795885	1.82929126
C	8.89470783	-0.46569296	2.02244275
C	9.68305702	0.32182611	1.19833898
C	9.10165073	1.09783024	0.15156326
C	9.90785745	1.91048451	-0.70107790
C	9.33725338	2.66322992	-1.71677469
C	7.93045881	2.63015018	-1.91675049
C	7.11853886	1.84972116	-1.10504978
C	7.66926189	1.06027536	-0.04907854
H	6.03277430	1.84007961	-1.27754912
H	7.47844133	3.22873049	-2.72198372
H	9.96960062	3.28599335	-2.36759659
H	10.99674882	1.92737301	-0.53469352
H	10.77516666	0.36127230	1.33873117
H	9.34986523	-1.06172945	2.82764626
H	6.88235078	-1.14289358	2.49463094
S	4.63798892	-0.67463345	-1.36833501
Si	2.46554206	-0.70372076	-1.51580303
C	2.05626943	-1.28523557	-3.26188466
C	1.30610957	-2.45193806	-3.42213005
C	0.88513543	-2.89295251	-4.70455452
C	1.21495413	-2.15511438	-5.83035974
C	2.00097126	-0.96799578	-5.72652585
C	2.36260684	-0.21364517	-6.88390513
C	3.14351307	0.92929394	-6.77718789
C	3.58509558	1.36717613	-5.49887470
C	3.23826606	0.66431037	-4.35284113
C	2.44086054	-0.51964033	-4.42304889

H	3.58208751	1.02667091	-3.37379596
H	4.20091339	2.27488780	-5.41329711
H	3.41869211	1.49805487	-7.67829887
H	2.01346984	-0.56319587	-7.86850299
H	0.88001762	-2.47835365	-6.82898861
H	0.28466286	-3.80979138	-4.78374028
H	1.01237365	-3.04758008	-2.54430609
S	1.55675581	1.26435447	-1.35245468
S	1.61045654	-2.14517876	-0.14716820
Si	2.12789214	-1.32082424	1.79446794
C	1.43281824	-2.54694468	3.04665181
C	0.47339381	-2.10054604	3.95768478
C	-0.16208605	-2.99181060	4.86215437
C	0.16402998	-4.33865913	4.84661675

C	1.15494097	-4.84459790	3.95216274
C	1.51044544	-6.22768323	3.94457885
C	2.48804680	-6.70979477	3.08468643
C	3.14263252	-5.82108979	2.18884142
C	2.81000247	-4.47329251	2.16151943
C	1.81311994	-3.93896301	3.03531855
H	3.32338111	-3.80629431	1.45458627
H	3.91503760	-6.20279311	1.50461577
H	2.75517826	-7.77742066	3.09285975
H	0.99527775	-6.90585079	4.64331957
H	-0.33397589	-5.04276085	5.53232427
H	-0.92145347	-2.60026122	5.55318717
H	0.17900552	-1.04010174	3.97820163
S	4.27825481	-1.23979334	2.17011095

Table S20. Cartesian coordinates of $[(NpSi)_4S_6]_2$ (stacking).

S	1.69516822	-2.00385067	0.62028539
Si	1.92343103	-1.51099154	-1.48529132
C	0.63714124	-2.44990473	-2.49204532
C	-0.00630630	-1.75829277	-3.52050695
C	-1.05479207	-2.35139055	-4.26815552
C	-1.50821940	-3.61911388	-3.94176390
C	-0.86373296	-4.38107067	-2.92325961
C	-1.29489685	-5.70346740	-2.60415214
C	-0.60959642	-6.47385489	-1.67544165
C	0.54679932	-5.94830085	-1.03675402
C	0.96916308	-4.65062383	-1.29623524
C	0.26786602	-3.81630272	-2.21939807
H	1.86472019	-4.26359612	-0.78964954
H	1.11649411	-6.57239945	-0.33177786
H	-0.94698360	-7.49604618	-1.44598313
H	-2.17606087	-6.10625321	-3.12807225
H	-2.36752162	-4.05759740	-4.47293672
H	-1.52810687	-1.78193066	-5.07902146
H	0.27596585	-0.71843526	-3.74628770
S	3.82387247	-2.14352039	-2.31414866
Si	5.26677883	-0.94094242	-1.21264565
C	6.97520018	-1.37998066	-1.88795886
C	7.93143338	-1.89587108	-1.01036289
C	9.23444824	-2.24549079	-1.45504072
C	9.57975209	-2.07606211	-2.78653118
C	8.64224562	-1.55168017	-3.72568291
C	8.99242903	-1.37344367	-5.09787591
C	8.07760802	-0.86204627	-6.00641644
C	6.77144640	-0.50821873	-5.57192845
C	6.39927522	-0.66948791	-4.24419642
C	7.31329907	-1.19354222	-3.27905430
H	5.38351511	-0.38750203	-3.93163592
H	6.04625200	-0.10187889	-6.29295782
H	8.35958717	-0.72910133	-7.06193158
H	10.00829873	-1.65142127	-5.42103589
H	10.58749323	-2.34413202	-3.14234913
H	9.96266320	-2.64990244	-0.73605724
H	7.68066302	-2.03805867	0.05241534
S	5.00898336	1.19022343	-1.56652610
Si	3.03178907	1.64836999	-0.76601110
C	2.95413725	3.53740996	-0.90656125
C	4.02911024	4.15203511	-0.24894777
C	4.20316611	5.55926822	-0.25744806
C	3.29842985	6.36066451	-0.93630751
C	2.18394671	5.79053579	-1.61703883
C	1.24160992	6.61697263	-2.29814828
C	0.13655054	6.07060497	-2.93144834
C	-0.06499146	4.66651066	-2.90293285

C	0.83962490	3.83538981	-2.25811674
C	1.99102519	4.35503742	-1.59780466
H	0.64795075	2.75639053	-2.24121026
H	-0.95596540	4.22960942	-3.37755172
H	-0.59375183	6.72146243	-3.43355026
H	1.40243173	7.70631947	-2.29440000
H	3.42434089	7.45506066	-0.95768711
H	5.05930628	6.00363642	0.27192946
H	4.76730097	3.53362794	0.28687240
S	1.44854237	0.58999575	-1.79695049
S	2.98062582	1.28911516	1.37954488
Si	3.28023699	-0.85363527	1.55679026
C	3.19546555	-1.24138899	3.39734769
C	2.24128078	-2.15812966	3.84291491
C	2.10600223	-2.47189824	5.22034413
C	2.92349110	-1.85739689	6.15422406
C	3.91845451	-0.91653824	5.75391629
C	4.76827420	-0.28508384	6.71106681
C	5.74065767	0.62114965	6.31433729
C	5.90093932	0.92681808	4.93566565
C	5.08861845	0.33143065	3.97978396
C	4.07126576	-0.60106823	4.35031219
H	5.23513244	0.58001765	2.91876428
H	6.67699673	1.64047420	4.61997768
H	6.39010897	1.10094694	7.06227029
H	4.63630988	-0.53561098	7.77578981
H	2.81673254	-2.08763323	7.22652716
H	1.33969054	-3.19237211	5.53475877
H	1.56947101	-2.65379766	3.12553826
S	5.26515513	-1.47119831	0.89509329
S	-1.92961840	1.87325587	-1.54011709
Si	-1.70441512	2.20426570	0.60469810
C	-0.68332443	3.76984207	0.83187788
C	0.49385617	3.67591412	1.57555512
C	1.29506151	4.81498928	1.84699221
C	0.89473912	6.06122116	1.39539080
C	-0.31221557	6.21828384	0.65075147
C	-0.73842381	7.50346113	0.20149130
C	-1.91074338	7.65488245	-0.52441931
C	-2.69447025	6.51394913	-0.84289905
C	-2.30549261	5.24849201	-0.42236330
C	-1.11592310	5.05697445	0.34290088
H	-2.92471565	4.37848480	-0.68430957
H	-3.61922251	6.63045274	-1.42812335
H	-2.22976693	8.65268996	-0.86186931
H	-0.11296721	8.37685303	0.44549860
H	1.50422496	6.95427767	1.60381147
H	2.22882781	4.69513095	2.41489068

H	0.82377573	2.70115608	1.96266978
S	-3.60893032	2.65876431	1.56968280
Si	-4.78019347	0.85850831	1.26064009
C	-6.47334468	1.15750206	2.04187562
C	-6.72819740	2.39427836	2.63848163
C	-7.98203819	2.68316231	3.24042472
C	-8.98525207	1.72693087	3.24269645
C	-8.77887288	0.44700075	2.64674832
C	-9.80809358	-0.54197938	2.64534213
C	-9.60195367	-1.78480158	2.06503031
C	-8.35041037	-2.08481745	1.46193707
C	-7.32899156	-1.14478181	1.44629432
C	-7.50344536	0.14623429	2.03326486
H	-6.36865070	-1.39971735	0.97549792
H	-8.18716889	-3.07152716	1.00282684
H	-10.40343165	-2.53904616	2.07047370
H	-10.77305594	-0.29645327	3.11676193
H	-9.96167246	1.94055725	3.70632580
H	-8.14809440	3.66817982	3.70186025
H	-5.94702528	3.17038320	2.64712936
S	-3.90402243	-0.84285034	2.27797374
Si	-1.96541254	-1.09939022	1.31491783
C	-1.31361837	-2.62608049	2.21503252
C	-1.08821447	-3.79149603	1.47969123
C	-0.80317341	-5.02572573	2.12035471
C	-0.79079487	-5.10385432	3.50416432
C	-1.02052785	-3.94478228	4.30401518
C	-1.03124333	-4.01539427	5.73051552
C	-1.21812223	-2.87476583	6.49909940
C	-1.37630285	-1.61266553	5.86525518

C	-1.38915512	-1.51361302	4.48062035
C	-1.24006007	-2.67034064	3.65556211
H	-1.50453664	-0.52686049	4.00890813
H	-1.48993352	-0.70428193	6.47522088
H	-1.22403242	-2.94176651	7.59751636
H	-0.88065404	-4.99634854	6.20900145
H	-0.60553810	-6.06493211	4.00998081
H	-0.61805613	-5.91637297	1.50340790
H	-1.13713533	-3.77478060	0.38036364
S	-0.61708642	0.58807511	1.56281714
S	-2.14658668	-1.63871702	-0.77848924
Si	-3.15627073	0.08932283	-1.63026685
C	-3.43542757	-0.28575932	-3.46001639
C	-2.92657737	0.59871577	-4.41273344
C	-3.08851828	0.36334782	-5.80457629
C	-3.74946476	-0.77494091	-6.24168849
C	-4.28427095	-1.71418905	-5.30918112
C	-4.92637248	-2.91031288	-5.75071772
C	-5.42270972	-3.83078965	-4.83851809
C	-5.30473510	-3.57952727	-3.44419813
C	-4.68813279	-2.42425883	-2.98363349
C	-4.14482232	-1.46486930	-3.89135699
H	-4.60161890	-2.25397885	-1.90127563
H	-5.70329090	-4.30691307	-2.72121759
H	-5.91024455	-4.75228109	-5.19114871
H	-5.01607457	-3.09005036	-6.83376634
H	-3.86443011	-0.97706857	-7.31848947
H	-2.67471719	1.08107759	-6.52857919
H	-2.37664276	1.49570487	-4.08831351
S	-5.14824442	0.45759789	-0.84779364

Table S21. Cartesian coordinates of $[(PhSn)_4S_6]_2$ (alternating).

S	1.41948178	1.66184291	-1.69918952
Sn	2.17873153	-0.62232735	-2.10927277
Sn	2.20633594	2.11792205	0.56467971
S	1.40539653	0.63463288	2.32882700
S	1.37924909	-2.33953776	-0.57478044
Sn	2.19179771	-1.55480432	1.59110019
S	4.63389212	-1.66153583	1.65544443
S	4.62404436	-0.66075269	-2.27281268
S	4.65360203	2.24963873	0.59990310
Sn	5.31956268	-0.01644569	-0.02196207
C	1.39041306	-1.15495553	-4.03019053
C	1.22386355	-0.16691543	-5.01882707
C	0.95829755	-2.47483507	-4.26105338
C	0.61015880	-0.50149526	-6.23886238
H	1.55352664	0.86791387	-4.83664534
C	0.34609600	-2.80190281	-5.48281509
H	1.07725126	-3.24680626	-3.48458217
C	0.16728929	-1.81567835	-6.46872435
H	0.47416292	0.26949992	-7.01303882
H	0.00236793	-3.83208338	-5.66364292
H	-0.31927032	-2.07273027	-7.42172405
C	1.41380267	-2.94513086	3.02599502
C	1.22405909	-4.29290008	2.66659300
C	1.01219725	-2.48225186	4.29345081
C	0.61711777	-5.17616782	3.57679413
H	1.53122613	-4.65523762	1.67302240
C	0.40683706	-3.37074890	5.19832026
H	1.15043973	-1.42594612	4.57283317
C	0.20435212	-4.71473122	4.83887383
H	0.46299086	-6.22998692	3.29751838
H	0.08731096	-3.00993167	6.18819853
H	-0.27668645	-5.40670281	5.54654891

C	1.44433349	4.06178837	1.05078713
C	1.24227630	4.41908251	2.39726995
C	1.06336966	4.93704010	0.01600656
C	0.64395859	5.65327386	2.70648600
H	1.53204082	3.73201675	3.20768887
C	0.46609822	6.16903799	0.33256935
H	1.21071679	4.65485252	-1.03828822
C	0.25150068	6.52476585	1.67567158
H	0.47998590	5.93418139	3.75838051
H	0.16241988	6.85309566	-0.47490570
H	-0.22330352	7.48686271	1.92052931
C	7.46754938	-0.00941562	-0.05276231
C	8.17888481	0.85916890	0.79853430
C	8.15976823	-0.87570616	-0.92196367
C	9.58444430	0.85814481	0.77898046
H	7.64122699	1.54274686	1.47467231
C	9.56539446	-0.87175727	-0.93656110
H	7.60707564	-1.55144335	-1.59385950
C	10.27623508	-0.00677009	-0.08661603
H	10.14088709	1.53782326	1.44242238
H	10.10690730	-1.54696917	-1.61671110
H	11.37683048	-0.00526934	-0.10035412
S	-1.41244445	-1.67265939	1.73929705
Sn	-2.20734606	-2.12760316	-0.51979238
Sn	-2.19358960	0.60801129	2.13568709
S	-1.37942102	2.33291408	0.61235277
S	-1.41443935	-0.64042519	-2.28677498
Sn	-2.17774931	1.55914538	-1.55916008
S	-4.62270270	1.69058785	-1.66897699
S	-4.65436736	-2.25091655	-0.58415076
S	-4.63613443	0.64831717	2.24802364
Sn	-5.32007440	0.03017820	-0.01900941

WILEY-VCH

C	-1.44854684	-4.06967677	-1.01804252
C	-1.02611550	-4.93365932	0.01013470
C	-1.29245330	-4.43787326	-2.36768354
C	-0.43379978	-6.16530842	-0.31641360
H	-1.13744110	-4.64270575	1.06652902
C	-0.69849133	-5.67174932	-2.68701006
H	-1.61551567	-3.76008255	-3.17329828
C	-0.26504904	-6.53193595	-1.66316174
H	-0.09772253	-6.84029088	0.48582389
H	-0.57067364	-5.96142577	-3.74153820
H	0.20595531	-7.49376182	-1.91629975
C	-1.38688343	2.93857093	-2.99670503
C	-0.99801581	2.47045577	-4.26620081
C	-1.17528772	4.28289777	-2.63684657
C	-0.38381712	3.35076172	-5.17312872
H	-1.15245482	1.41643827	-4.54562310
C	-0.55963043	5.15774523	-3.54922950
H	-1.47119823	4.64867993	-1.64115706
C	-0.15986960	4.69132710	-4.81366376
H	-0.07416055	2.98597420	-6.16467292
H	-0.38806658	6.20878869	-3.26965948
H	0.32823002	5.37661815	-5.52299534

C	-1.41506687	1.14421411	4.06017441
C	-1.01194015	2.47130582	4.30163739
C	-1.22432829	0.15197430	5.04028276
C	-0.40407726	2.80149988	5.52485315
H	-1.15059608	3.24695837	3.53217801
C	-0.61536384	0.48952462	6.26178749
H	-1.53165059	-0.88829379	4.84992765
C	-0.20099414	1.81117492	6.50186590
H	-0.08314855	3.83754281	5.71407379
H	-0.46059172	-0.28481031	7.02907650
H	0.28191490	2.07089299	7.45599999
C	-7.46810058	0.03702734	-0.04600896
C	-8.16279878	1.21599035	-0.38142633
C	-8.17691426	-1.13673047	0.27811806
C	-9.56848001	1.21817433	-0.39059838
H	-7.61209649	2.13385593	-0.64174173
C	-9.58254246	-1.12778787	0.26700751
H	-7.63716952	-2.06217199	0.53456714
C	-10.27681800	0.04825904	-0.06585085
H	-10.11202829	2.13839490	-0.65422028
H	-10.13707056	-2.04487089	0.51842195
H	-11.37746249	0.05255116	-0.07407589

Table S22. Cartesian coordinates of [(PhSn)$_4$S$_6$]$_2$ (stacking).

Sn	-1.75512245	0.97561227	1.59394100
Sn	1.68205188	-1.95181646	0.32488213
Sn	5.23577559	-0.67428222	-0.41096217
Sn	-2.01508399	0.41048226	-2.21300618
Sn	3.09554611	1.10799072	2.22903987
Sn	-5.18461427	0.93994740	-0.15120413
Sn	-3.19021808	-2.32485541	0.16037067
Sn	2.22010977	1.42212431	-1.46797692
S	-1.67985014	-1.45289507	1.87569818
S	1.06682346	-0.73766345	-1.69827044
S	-4.28254193	1.35773664	-2.37988527
S	5.39282161	0.45185780	1.76104193
S	-4.03125462	1.87778562	1.78344718
S	1.50096650	-0.73464832	2.43907613
S	2.23467369	2.71628004	0.61547330
S	-5.46717324	-1.46500582	0.23029566
S	3.96846033	-2.73670371	-0.10874226
S	-0.94261023	1.85120112	-0.53171720
S	-2.15485568	-2.03134519	-2.02484517
S	4.53091849	0.89518848	-2.14033196
C	-0.73662784	1.93131943	3.21846326
C	0.57226277	-3.78116264	0.43025610
C	7.22573522	-1.27929465	-0.94955469
C	-1.16017270	0.77919140	-4.14957074
C	3.23940512	2.05700170	4.14571466
C	-7.14768615	1.81307392	-0.14817230
C	-3.45247753	-4.41851999	0.53840618
C	1.56766077	2.79347278	-2.98883502
C	-0.63645504	1.28248272	4.46411666
C	0.20099995	-4.42661885	-0.76473566
C	8.02556759	-0.43724767	-1.74724715
C	-1.52245303	1.95492801	-4.83452167
C	3.12422495	1.28464868	5.31654299
C	-7.72948531	2.22591785	1.06696949
C	-3.45422131	-4.89183615	1.86376451
C	0.76010981	3.90118756	-2.66691459
C	-0.11640033	1.97961989	5.56765348
C	-0.38468971	-5.70315187	-0.70844285
C	9.32992834	-0.83376852	-2.09001097
C	-1.06484801	2.16279283	-6.14612519

C	3.31963462	1.88704154	6.57154232
C	-9.01728062	2.78939446	1.06772276
C	-3.73047127	-6.24610464	2.12029610
C	0.44296124	4.84356592	-3.66030141
C	0.30060015	3.31339063	5.42689868
C	-0.58786682	-6.33216563	0.53013931
C	9.83320304	-2.06599106	-1.63802292
C	-0.24610258	1.20557014	-6.76869087
C	3.62853004	3.25552755	6.65490768
C	-9.72112299	2.93961185	-0.13977486
C	-4.00307263	-7.12272443	1.05629188
C	0.93877550	4.68503201	-4.96512481
C	0.21815521	3.94969412	4.17813461
C	-0.23054093	-5.67770514	1.72041708
C	3.74060782	4.02620925	5.48431308
C	-9.13922310	2.52880462	-1.35162891
C	9.03380985	-2.90618161	-0.84360459
C	0.12378869	0.03912479	-6.07881012
C	-3.99828171	-6.64871066	-0.26741782
C	1.74076727	3.57634934	-5.28399750
C	-0.30556411	3.26333198	3.06897500
C	0.35063618	-4.39927158	1.67588633
C	3.54744090	3.42835628	4.22719991
C	-7.85165410	1.96477077	-1.35920307
C	7.72873533	-2.51539610	-0.49723969
C	-0.33640197	-0.18023496	-4.76897185
C	-3.72438510	-5.29539833	-0.52902421
C	2.05606289	2.62604024	-4.29883723
H	-0.96223951	0.23672973	4.57669624
H	0.36277860	-3.93915807	-1.73782768
H	7.63338870	0.52720909	-2.10733489
H	-2.16605323	2.70803148	-4.35437770
H	2.87860884	0.21283367	5.25543814
H	-7.17986134	2.11482538	2.01519518
H	-3.23714607	-4.20893896	2.70002649
H	0.37752109	4.03639943	-1.64401967
H	-0.03347168	1.47635459	6.54263216
H	-0.68459323	-6.20707535	-1.63946636
H	9.95492817	-0.17673175	-2.71410625
H	-1.34782700	3.08135375	-6.68157682

H	3.23030545	1.28360652	7.48786303
H	-9.47164741	3.11360147	2.01652842
H	-3.73261277	-6.61709870	3.15669068
H	-0.18859448	5.70899596	-3.40793826
H	0.71565014	3.85180208	6.29066572
H	-1.05074708	-7.32836430	0.57014138
H	10.85473244	-2.37440763	-1.90806703
H	0.10965997	1.37241966	-7.79686605
H	3.78516363	3.72453683	7.63833624
H	-10.72929552	3.38136004	-0.13651079
H	-4.22300349	-8.18193851	1.25957999
H	0.69519115	5.42788612	-5.73997819
H	0.56479712	4.98764752	4.06327150
H	-0.40805139	-6.16411662	2.69132864
H	3.98170848	5.09832419	5.54916531

H	-9.68928276	2.64849378	-2.29763155
H	9.42674339	-3.87245133	-0.49186085
H	0.76833299	-0.71080290	-6.56220286
H	-4.21114780	-7.33509781	-1.10127570
H	2.12242573	3.44541397	-6.30762545
H	-0.37275760	3.76721034	2.09315387
H	0.62991954	-3.88819082	2.61039160
H	3.63585295	4.03582836	3.31276565
H	-7.39752096	1.64834695	-2.31170834
H	7.10428310	-3.17945100	0.12135173
H	-0.05170040	-1.10051599	-4.23696127
H	-3.72229490	-4.92834986	-1.56744404
H	2.68832164	1.76209307	-4.55529368

Table S23. Cartesian coordinates of $[(NpSn)_4S_6]_2$ (alternating).

Sn	2.0873546	2.3834615	0.0284209
Sn	-1.7363065	-2.1643846	0.8306481
Sn	-5.3414291	-0.8077556	0.7806772
S	2.1702178	1.6516887	2.3640015
S	-4.0348280	-2.7280220	1.4977455
S	0.9074288	0.8700451	-1.4657284
S	-0.7953537	-0.2055957	1.9416956
S	-5.3611679	-0.5700953	-1.6592870
C	1.0142393	4.2463541	-0.0472426
C	-0.7175330	-3.9709368	1.4203640
C	-7.3783734	-1.2253856	1.3506519
C	1.3539133	5.3335265	0.8230411
C	0.1733994	-3.9407265	2.4890258
C	-8.4292039	-0.2959702	1.0540431
C	0.6133860	6.5685047	0.6885998
C	0.9162685	-5.1024634	2.8371192
C	-9.7799994	-0.6399324	1.4444811
C	-0.4275173	6.6597644	-0.2828123
C	0.7651082	-6.2696567	2.1043612
C	-10.0175521	-1.8801085	2.1101412
C	-0.7292350	5.5877995	-1.1075941
C	-0.1672131	-6.3487815	1.0270317
C	-8.9774381	-2.7551695	2.3853528
C	0.0088832	4.3773297	-0.9993854
C	-0.9480519	-5.1786863	0.6817708
C	-7.6470326	-2.4258550	2.0010316
H	0.3314773	-3.0154698	3.0646939
H	1.6200769	-5.0624467	3.6789951
H	-0.9960078	7.5988771	-0.3572869
H	1.3577794	-7.1634593	2.3563161
H	-11.0496158	-2.1303930	2.4032962
H	-1.5404283	5.6590851	-1.8466033
H	-9.1727100	-3.7082897	2.8997891
H	-0.2395619	3.5399568	-1.6670852
H	-6.8319948	-3.1336044	2.2233767
S	4.4064554	2.8889003	-0.6028255
S	-1.5267473	-1.9074316	-1.5957920
S	-4.6632176	1.2133020	1.9770290
Sn	3.1330571	-0.5777591	2.2019243
Sn	2.2712796	-1.1640031	-1.5035080
Sn	-2.3444983	1.5689806	1.2739985
Sn	-3.0196302	-0.0698827	-2.1611817
Sn	5.4853528	0.7013997	-0.5133385
S	2.1223818	-2.1967774	0.6933493
S	5.5162380	-0.3685949	1.7035815
C	2.5978985	-1.4899941	4.0801979
C	1.3722620	-2.7106831	-2.7005968

S	4.5722085	-0.7835808	-2.2311622
S	-2.4303998	2.0567831	-1.1230178
C	-1.6846317	3.3663049	2.2606559
C	-3.0007656	0.1679658	-4.3102921
C	7.5494185	1.0644431	-1.0186640
C	3.1049231	-2.7794340	4.4459346
C	1.5700984	-0.8938807	4.8051476
C	0.7697103	-2.4253585	-3.9663716
C	1.2398104	-3.9600087	-2.1021476
C	-0.6777375	3.2632067	3.2141254
C	-2.3049636	4.6282072	1.9777533
C	-2.1002194	1.0527749	-4.9892257
C	-3.9560055	-0.5546271	-5.0201032
C	7.9453491	2.3758191	-1.2635069
C	8.4866131	-0.0178583	-1.1018086
C	2.5294886	-3.4419793	5.5972805
H	1.1643999	0.0816381	4.4942548
C	1.0087315	-1.5519268	5.9353794
C	-0.0376829	-3.4552380	-4.5843853
C	0.4793752	-4.9807351	-2.7364463
H	1.6902505	-4.1660785	-1.1178800
H	-0.2175396	2.2887984	3.4328007
C	-0.2173083	4.4162762	3.9071758
C	-1.8464430	5.7907597	2.7058352
C	-2.2382896	1.2122929	-6.4209834
C	-4.0690262	-0.4128169	-6.4313914
H	-4.6456246	-1.2348976	-4.4945660
H	7.2171597	3.2001986	-1.1964986
C	9.2958584	2.6688162	-1.6039761
C	9.8583655	0.2891560	-1.4477979
C	1.4795184	-2.7984738	6.3193969
H	0.1879579	-1.0709228	6.4880744
C	-0.1609224	-4.7224203	-3.9387376
H	0.3791289	-5.9592070	-2.2458228
H	0.6006897	4.3171221	4.6355805
C	-0.7954310	5.6510094	3.6599738
C	-3.2363093	0.4625726	-7.1120947
H	-4.8286975	-0.9949927	-6.9745897
H	9.5923534	3.7111988	-1.7953726
C	10.2280082	1.6459694	-1.6926325
H	1.0388475	-3.3197277	7.1837726
H	-0.7831229	-5.4969233	-4.4145099
H	-0.4435355	6.5488460	4.1896997
H	-3.3277762	0.5863158	-8.2029984
H	11.2749489	1.8666808	-1.9551001
C	0.9343872	7.6637447	1.5438654
C	1.9453047	7.5609879	2.4891266

C	2.3895407	5.2643155	1.8029391
C	2.6800904	6.3502623	2.6164433
H	2.9675356	4.3336428	1.9171501
H	3.4825757	6.2716033	3.3650266
H	2.1816456	8.4155530	3.1412837
H	0.3572564	8.5958432	1.4388845
C	-0.3660071	-7.5568398	0.2920553
C	-1.3173482	-7.6323243	-0.7162670
C	-1.9180461	-5.2955026	-0.3606162
C	-2.1052904	-6.4923423	-1.0365419
H	-2.5302861	-4.4229363	-0.6349333
H	-2.8625499	-6.5539259	-1.8321085
H	-1.4664949	-8.5737517	-1.2665536
H	0.2423002	-8.4370156	0.5546286
C	-10.8393891	0.2710956	1.1528566
C	-10.5888761	1.4724090	0.5054035
C	-8.2092370	0.9501068	0.3927193
C	-9.2618268	1.8133616	0.1236604
H	-7.1874833	1.2303871	0.0897008
H	-9.0673507	2.7676583	-0.3882684
H	-11.4155436	2.1651876	0.2865103
H	-11.8640735	0.0003112	1.4535646
C	3.0032838	-4.7401095	5.9567611
C	3.9935286	-5.3689361	5.2140352
C	4.1239431	-3.4544554	3.7091357
C	4.5564734	-4.7199637	4.0800369
H	4.5685401	-2.9638482	2.8279126
H	5.3369301	-5.2245151	3.4915146
H	4.3428183	-6.3730245	5.4981787
H	2.5581075	-5.2395271	6.8317221
C	-0.6939238	-3.1668931	-5.8181100
C	-0.5451561	-1.9322768	-6.4336346
C	0.9042239	-1.1718729	-4.6298737
C	0.2706075	-0.9311443	-5.8400438
H	1.5172498	-0.3794213	-4.1694600
H	0.3869724	0.0432684	-6.3345933
H	-1.0634018	-1.7188797	-7.3799218
H	-1.3226759	-3.9478033	-6.2746890
C	-2.4494283	7.0559199	2.4408322
C	-3.4680371	7.1813370	1.5067416
C	-3.3581085	4.7934456	1.0284505
C	-3.9272024	6.0376465	0.7976518
H	-3.7232355	3.9201576	0.4660308
H	-4.7367652	6.1382815	0.0593913
H	-3.9238322	8.1645461	1.3141928
H	-2.0846208	7.9352606	2.9947889
C	-1.3457672	2.0883766	-7.1105887
C	-0.3448593	2.7704182	-6.4310728
C	-1.0528134	1.7607801	-4.3300307
C	-0.1922683	2.5954790	-5.0273703
H	-0.9013195	1.6165367	-3.2511176
H	0.6189413	3.1087969	-4.4895387
H	0.3402925	3.4354514	-6.9782415
H	-1.4593398	2.2028191	-8.2003774
C	10.8064050	-0.7741306	-1.5353663
C	10.4293503	-2.0874250	-1.2942742
C	8.1351410	-1.3807901	-0.8623144
C	9.0819169	-2.3910628	-0.9553438
H	7.0953755	-1.6357028	-0.6011207
H	8.7864866	-3.4340410	-0.7668847
H	11.1708338	-2.8975190	-1.3657097
H	11.8476819	-0.5299906	-1.7995379

Table S24. Cartesian coordinates of [(NpSn)$_4$S$_6$]$_2$ (stacking).

Sn	1.7489252	1.8659662	-1.2974236
Sn	-2.6582011	-2.1907994	1.6035694
Sn	-5.5788950	-0.1583348	0.2383670
S	1.2090544	1.7754018	1.0870941
S	-5.0959065	-2.0495622	1.7209726
S	1.3344736	-0.0470740	-2.7521256
S	-1.4903391	-0.2178178	2.4447276
S	-4.9528476	-0.6153720	-2.0900454
C	0.8586779	3.6360277	-2.1426962
C	-2.2187253	-3.6560676	3.1357370
C	-7.7302214	-0.0123447	0.2148432
C	0.0953216	3.5121641	-3.2987634
C	-3.0282682	-3.5763433	4.2669859
C	-8.4696722	-0.9529320	0.9247474
C	-0.4434994	4.6676360	-3.9283532
C	-2.7691040	-4.3969606	5.4005750
C	-9.8923235	-0.9090257	0.9131077
C	-0.1982178	5.9272443	-3.4019951
C	-1.6938489	-5.2739273	5.3952897
C	-10.5519419	0.0722343	0.1883408
C	0.5925326	6.0917170	-2.2253055
C	-0.8380138	-5.3781133	4.2576094
C	-9.8266891	1.0526662	-0.5534344
C	1.1273622	4.9202829	-1.5634676
C	-1.1080443	-4.5597068	3.0937118
C	-8.3796903	1.0171838	-0.5435013
H	-0.1083760	2.5240140	-3.7395218
H	-3.8752321	-2.8719653	4.2980757
H	-7.9607317	-1.7427454	1.5006154
H	-1.0475439	4.5489860	-4.8396477
H	-3.4213389	-4.3206452	6.2835821
H	-10.4624029	-1.6603232	1.4803371
H	-0.6064062	6.8260602	-3.8912594
H	-1.4794674	-5.9007508	6.2753438
H	-11.6528610	0.1095425	0.1732296
S	4.1486362	2.4579994	-1.4337886
S	-1.8939387	-2.7704130	-0.6380259
S	-4.6532396	1.9176888	1.0974273
Sn	2.3160139	-0.2622331	1.8556373
Sn	2.5589573	-1.8546001	-1.6543306
Sn	-2.2102288	1.6111629	1.0009479
Sn	-2.5234891	-0.8270300	-1.9824478
Sn	5.2821648	0.5602987	-0.4405269
S	1.7031584	-2.2623183	0.5965687
S	4.7294941	0.1183343	1.9051229
C	1.6490288	-0.6485294	3.8647802
C	2.3873596	-3.5386085	-3.0053538
S	4.9777768	-1.5129873	-1.7105498
S	-1.5911010	1.3385539	-1.3415763
C	-1.5272951	3.6030433	1.4461433
C	-1.6316140	-1.2286227	-3.8976298
C	7.3830238	1.0437335	-0.4880718
C	0.8953366	-1.7937962	4.0980084
C	1.9016766	0.2982345	4.9096330
C	3.2035934	-3.4478909	-4.1315577
C	1.4901420	-4.6427391	-2.8418046
C	-1.7299196	4.5335549	0.4302074
C	-0.9177015	3.9755839	2.6855313
C	-1.5536872	-0.1775750	-4.8688725
C	-0.9232818	-2.4132399	-4.0631329

C	7.7558935	2.2844286	-0.9963372
C	8.3667849	0.1191496	-0.0039777
H	0.7184019	-2.5220699	3.2919655
C	0.3275436	-2.0340480	5.3790346
C	1.3323246	0.0398225	6.2159121
C	3.1599147	-4.4502622	-5.1401493
H	3.8936429	-2.5974999	-4.2534769
C	1.4439962	-5.6559372	-3.8761207
H	-2.1845754	4.2315901	-0.5270145
C	-1.3374903	5.8872909	0.6133093
C	-0.4849098	5.3465332	2.8457046
C	-0.6578966	-0.3490637	-5.9917593
C	-0.0822018	-2.5935284	-5.1963307
H	-0.9728361	-3.2112073	-3.3055777
H	6.9908372	2.9867647	-1.3650405
C	9.1276310	2.6606826	-1.0462163
C	9.7598563	0.5091502	-0.0591381
H	-0.2832961	-2.9342966	5.5371506
C	0.5361992	-1.1292861	6.4095234
H	3.8126615	-4.3591895	-6.0214108
C	2.2952695	-5.5280281	-5.0148805
H	-1.5020858	6.6131497	-0.1952199
C	-0.7204787	6.2782135	1.7911665
C	0.0670799	-1.5717930	-6.1211150
H	0.4709167	-3.5356863	-5.3139092
H	9.4050149	3.6457774	-1.4510708
C	10.1043995	1.7897879	-0.5872849
H	0.0932463	-1.3051823	7.4027459
H	2.2501382	-6.3027407	-5.7966444
H	-0.3930435	7.3208842	1.9251201
H	0.7513275	-1.6916566	-6.9757964
H	11.1679742	2.0749490	-0.6230958
C	0.8701398	7.3830261	-1.6831116
C	1.6420451	7.5251861	-0.5390241
C	1.8985488	5.1052847	-0.3759360
C	2.1529170	6.3744208	0.1216292
H	2.2893880	4.2287420	0.1629295
H	2.7464742	6.4874749	1.0407606
H	1.8520553	8.5272577	-0.1350310
H	0.4608483	8.2677888	-2.1964841
C	0.2916970	-6.2503349	4.2429747
C	1.1240567	-6.3239850	3.1349400
C	-0.2397070	-4.6766920	1.9692467
C	0.8537462	-5.5310408	1.9865289
H	-0.4357346	-4.0582360	1.0810132
H	1.5140170	-5.5859049	1.1090386
H	1.9970346	-6.9936366	3.1426443
H	0.4950267	-6.8613406	5.1366549

C	-10.4903398	2.0674016	-1.3065643
C	-9.7682699	3.0114375	-2.0224244
C	-7.6704403	2.0063143	-1.2897577
C	-8.3464111	2.9794075	-2.0119435
H	-6.5685128	1.9997485	-1.2887529
H	-7.7775108	3.7315085	-2.5787844
H	-10.2932039	3.7879888	-2.5991723
H	-11.5918412	2.0858120	-1.3083996
C	1.5927350	0.9597472	7.2769768
C	2.3907263	2.0777765	7.0737438
C	2.6962245	1.4710730	4.7325260
C	2.9445429	2.3348056	5.7892506
H	3.1298099	1.6877110	3.7432139
H	3.5640586	3.2290585	5.6280060
H	2.5898492	2.7718963	7.9043085
H	1.1550188	0.7575537	8.2675057
C	0.5333857	-6.7466809	-3.7385832
C	-0.3001752	-6.8469437	-2.6337306
C	0.6236070	-4.7859114	-1.7189260
C	-0.2536125	-5.8564224	-1.6148861
H	0.6376900	-4.0156677	-0.9343990
H	-0.9188494	-5.9313706	-0.7424716
H	-1.0017407	-7.6897874	-2.5442456
H	0.5020590	-7.5075873	-4.5344782
C	0.1614409	5.7270663	4.0605410
C	0.3583364	4.8091339	5.0827670
C	-0.7112650	3.0645679	3.7633172
C	-0.0922011	3.4685196	4.9356260
H	-1.0516508	2.0219540	3.6663461
H	0.0587092	2.7463712	5.7502175
H	0.8610500	5.1143072	6.0130235
H	0.4974103	6.7705888	4.1715426
C	-0.5280573	0.7092704	-6.9401256
C	-1.2666334	1.8777068	-6.8132224
C	-2.3097328	1.0296867	-4.7784583
C	-2.1760746	2.0313370	-5.7306213
H	-3.0204860	1.1630503	-3.9471744
H	-2.7815842	2.9460019	-5.6458770
H	-1.1570779	2.6837413	-7.5543736
H	0.1680412	0.5773618	-7.7835029
C	10.7535962	-0.3982228	0.4164118
C	10.4002081	-1.6388785	0.9273638
C	8.0408267	-1.1648617	0.5287603
C	9.0320232	-2.0232632	0.9833027
H	6.9861170	-1.4796194	0.5799053
H	8.7560479	-3.0077948	1.3896570
H	11.1769712	-2.3286844	1.2908246
H	11.8109526	-0.0923749	0.3697228

Minimum structures of the cluster monomers calculated at DFT-PBE level of theory with a plane waves basis set and employed for the calculation of the optical response are given in Tables S25 and S26.

Table S25. Cartesian coordinates of [(PhSi)$_4$S$_6$].

Si	4.237155	4.292046	4.329780
Si	6.680245	6.625975	4.329780
Si	6.625546	4.237533	6.697234
Si	4.291854	6.680509	6.697234
S	5.458710	5.459021	2.997820
S	5.458710	5.459021	8.029275
S	2.922998	5.502447	5.518219
S	7.994401	5.415595	5.518219
S	5.415265	2.923295	5.508795

S	5.502135	7.994726	5.508795
C	3.186557	3.204852	3.230171
C	7.730842	7.713169	3.230171
C	7.712393	3.186862	7.797124
C	3.205006	7.731159	7.797124
C	3.824078	2.261930	2.402154
C	7.093321	8.656091	2.402154
C	8.652191	3.824611	8.628517
C	2.265208	7.093410	8.628517

C	3.077222	1.436860	1.560313
C	7.840177	9.481161	1.560313
C	9.476922	3.077670	9.470619
C	1.440477	7.840372	9.470619
C	1.683663	1.543186	1.533670
C	9.233737	9.374856	1.533670
C	9.373465	1.683818	9.494047
C	1.543934	9.234203	9.494047
C	1.039449	2.475879	2.349873
C	9.877951	8.442163	2.349873
C	8.443874	1.039396	8.674469
C	2.473546	9.878625	8.674469
C	1.785970	3.302246	3.193120
C	9.131450	7.615775	3.193120
C	7.617763	1.786003	7.831041
C	3.299637	9.132039	7.831041
H	4.912661	2.171522	2.415736
H	6.004738	8.746499	2.415736
H	8.740358	4.913395	8.617366
H	2.177041	6.004626	8.617366
H	3.583528	0.709770	0.923698
H	7.333872	10.208272	0.923698
H	10.201501	3.584138	10.109926
H	0.715898	7.333904	10.109926
H	1.099586	0.898095	0.875496
H	9.817813	10.019926	0.875496
H	10.018306	1.099682	10.152402
H	0.899093	9.818339	10.152402
H	-0.048047	2.561371	2.330986
H	10.965447	8.356671	2.330986
H	8.360498	-0.048300	8.690925
H	2.556902	10.966321	8.690925
H	1.270000	4.027495	3.825395
H	9.647399	6.890546	3.825395
H	6.894899	1.269892	7.196154
H	4.022500	9.648129	7.196154

Table S26. Cartesian coordinates of [(NpSi)$_4$S$_6$].

Si	5.996220	7.557732	14.051818
Si	8.185820	6.971769	11.512204
Si	7.768060	4.676868	13.963092
Si	5.289380	5.233095	11.697180
S	7.403720	8.549331	12.755314
S	9.266260	5.509014	12.661946
S	6.657820	6.094095	10.277432
S	6.216100	3.670422	12.857042
S	6.961600	6.125259	15.335078
S	4.353460	6.711222	12.949684
C	5.270120	8.883882	15.167834
C	8.624700	3.364389	14.999930
C	3.917860	4.419828	10.703066
C	9.426340	7.771407	10.349284
C	10.153640	7.017108	9.363420
C	9.643260	9.140208	10.447822
C	11.084040	7.706874	8.507884
C	10.003940	5.614917	9.184252
C	9.830380	1.284213	16.497998
C	10.333280	2.604945	16.626896
C	8.772580	1.011339	15.659952
C	10.558160	9.811158	9.605992
C	11.262940	9.106860	8.655768
C	11.424400	2.891364	17.489538
C	9.727980	3.670695	15.871152
C	11.808260	6.975927	7.529148
C	11.634120	5.616114	7.386280
C	10.723100	4.933320	8.223424
C	8.175200	2.052456	14.914878
C	11.912300	4.174401	17.614520
C	11.321820	5.224842	16.876376
C	10.260060	4.979352	16.029376
C	3.713380	3.053064	10.846594
C	3.076440	5.162199	9.802672
C	2.701780	2.372937	10.132452
C	1.886660	3.065916	9.265784
C	2.047660	4.463172	9.077244
C	1.209720	5.182359	8.184308
C	1.366520	6.539547	8.001884
C	2.373940	7.231392	8.711362
C	3.204600	6.561324	9.586544
C	5.707780	10.193631	15.014802
C	4.277600	8.590302	16.167184
C	5.203880	11.244765	15.813006
C	4.252280	10.984113	16.773372
C	3.766960	9.666153	16.976586
C	2.785580	9.392250	17.965816
C	2.314880	8.111817	18.162848
C	2.812880	7.051506	17.372476
C	3.767040	7.284753	16.402848
H	10.299980	0.486570	17.077852
H	10.699520	10.887093	9.715772
H	9.099840	9.727326	11.190278
H	11.972400	9.615858	7.999596
H	12.509080	7.516341	6.889190
H	12.196060	5.065914	6.630404
H	10.585580	3.857133	8.110498
H	9.308180	5.062848	9.816180
H	7.339320	1.802913	14.258882
H	8.389700	-0.005502	15.564494
H	11.870880	2.070075	18.054234
H	12.751320	4.381629	18.280108
H	11.707820	6.240276	16.975508
H	9.823820	5.808684	15.472512
H	4.342600	2.474913	11.525602
H	2.573740	1.299039	10.273956
H	1.101580	2.549799	8.708766
H	0.434980	4.635078	7.643020
H	0.716580	7.080822	7.313086
H	2.497680	8.305563	8.567350
H	3.972940	7.120260	10.120814
H	6.460380	10.433535	14.261566
H	5.573800	12.259548	15.661008
H	3.855280	11.789526	17.395246
H	2.410100	10.220973	18.569914
H	1.560800	7.914165	18.925874
H	2.440740	6.038130	17.528918
H	4.133700	6.448008	15.808166

WILEY-VCH

6 Calculation of Rotation Profiles

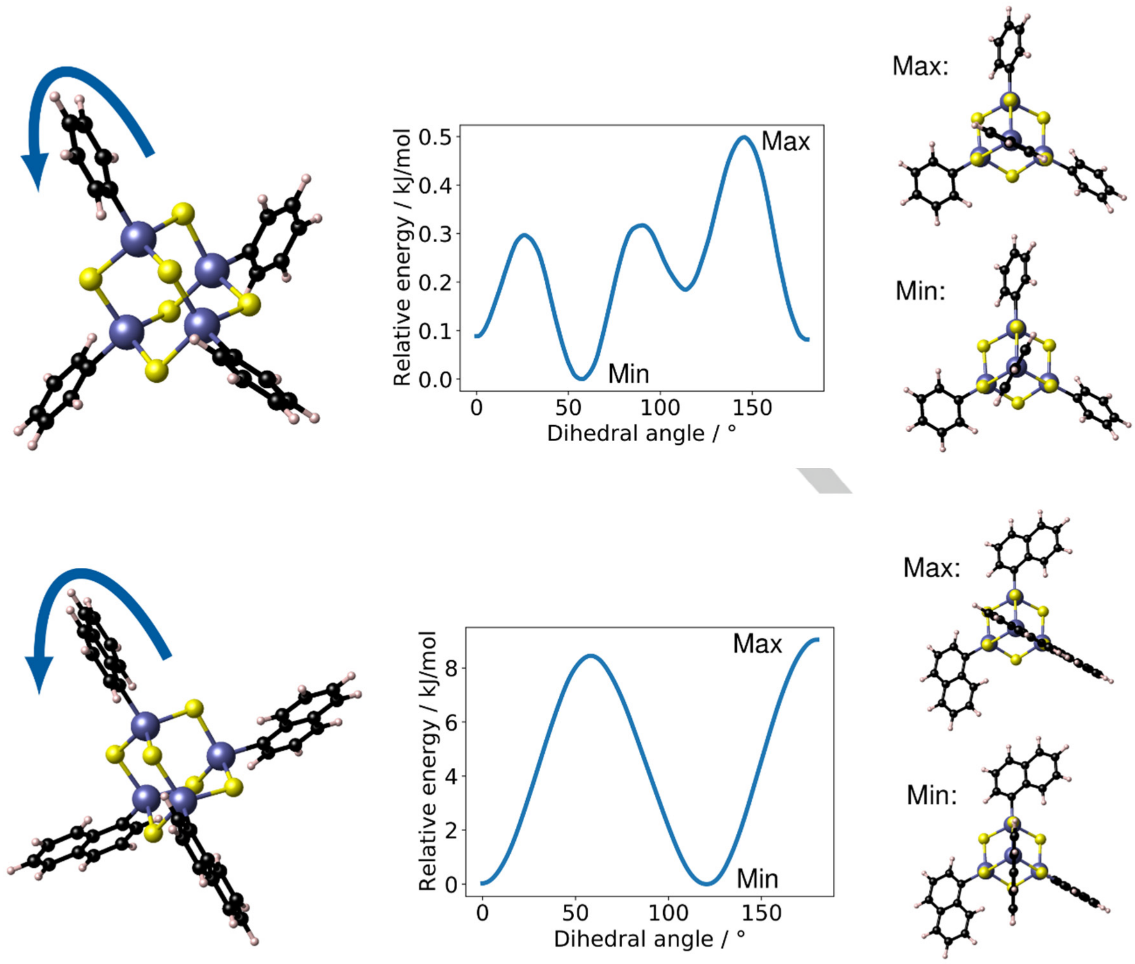

Figure S12. Scan of the phenyl and naphtyl rotation for [$(PhSn)_4S_6$] and [$(NpSn)_4S_6$] clusters. The corresponding scans for Si/S core clusters are given in Figure 4 in the main document.

WILEY

7 Analysis of the Structures

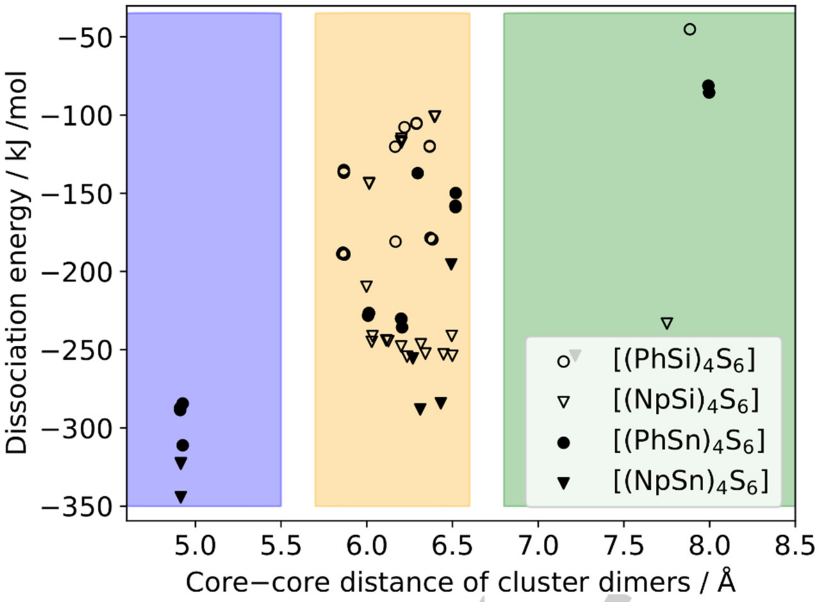

Figure S13. Dissociation energy of cluster dimers, plotted against the corresponding core−core distance calculated at the BP86-D3/cc-pVDZ(-PP) level of theory. Small core−core distances are marked by a purple background, medium core−core distances are marked by an orange background and large core−core distances by a green background.

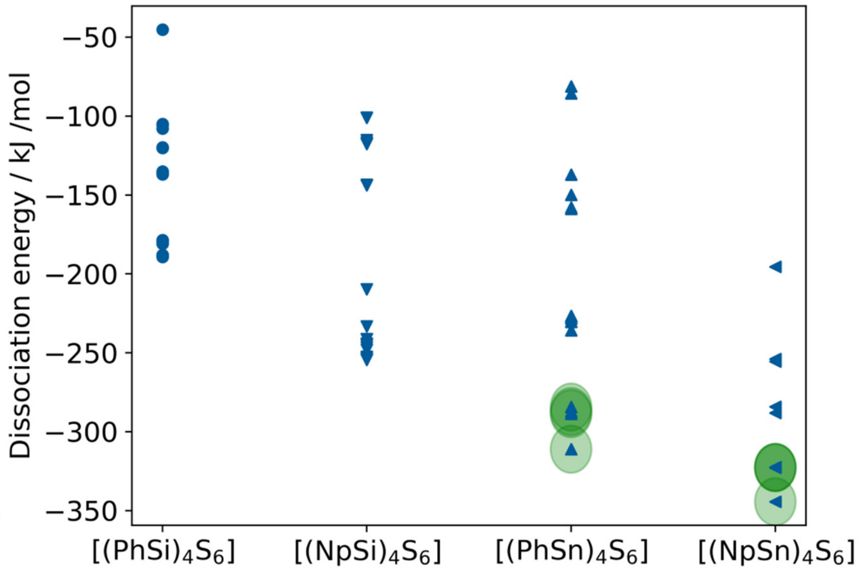

Figure S14. Dissociation energies of the calculated cluster dimers. The fused cluster dimers are marked with a green circle.

8 Calculation of Quasiparticle Gaps

The calculation of the quasiparticle gaps is performed by total energy calculations of charged molecules. Due to the interactions with the periodic images, the total energy of charged systems depends on the cell size. In order to correct the calculated excitation energies, the gaps were determined for cubic cells with edge L = 20, 25, 30, 35, 40 Å (Figure S15). The quasiparticle gap values depend linearly on $1/L$, and the extrapolation to $L \rightarrow \infty$ leads to the values of 6.5366 eV for [$(PhSi)_4S_6$] and 5.0909 eV for [$(NpSi)_4S_6$] clusters.

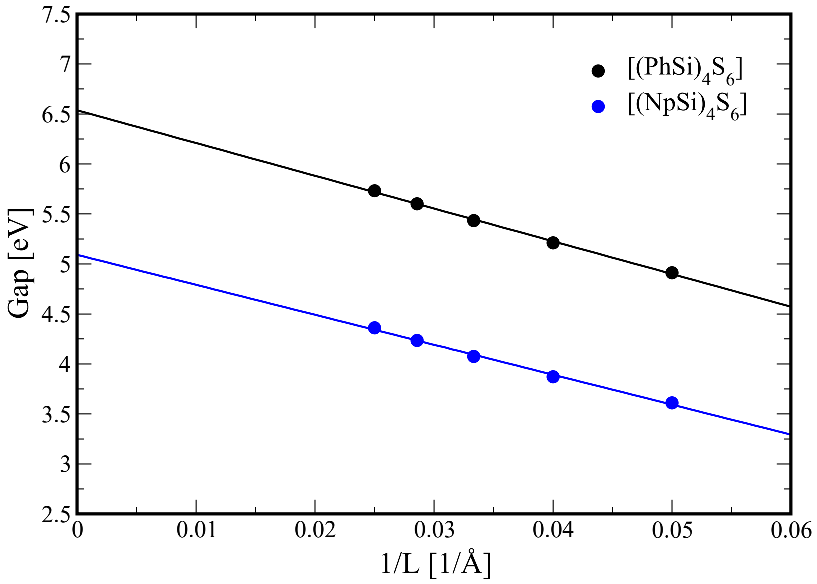

Figure S15. Dependence of the calculated quasiparticle gaps on the cell size.

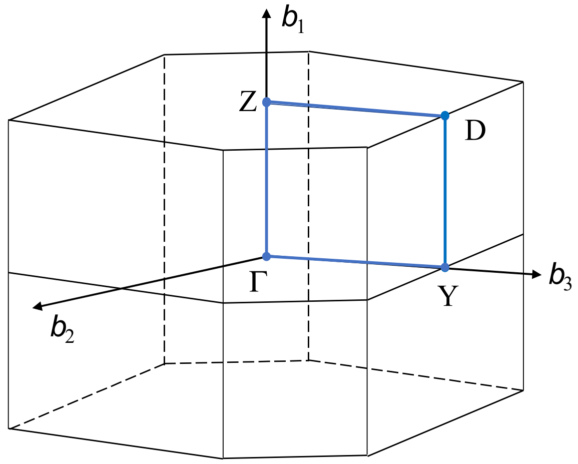

Figure S16. First Brillouin zone of crystals comprising [$(PhSi)_4S_6$] and [$(NpSi)_4S_6$] molecules. The path employed for the band structure calculations is highlighted.

9 Analysis of the Influence of Crystal Solvent

In order to clarify whether the solvent has any effect on the optical response of the clusters, we calculated the dielectric function of a supercell modelling toluene incorporated in the crystalline [$(NpSi)_4S_6$] structure, and of a supercell modelling an ideal [$(NpSi)_4S_6$] crystal without toluene. The corresponding absorption spectra are shown in Figure 17. The calculations are performed both with the atomic positions as determined by X-ray diffraction experiments (solid lines) and with the DFT equilibrium geometry (dashed lines). Although the structural optimization redistributes the spectral weight of the different features, the extinction coefficient calculated with and without toluene does not substantially differ. This demonstrates that the optical response of the molecular cluster is not affected by the presence of solvent.

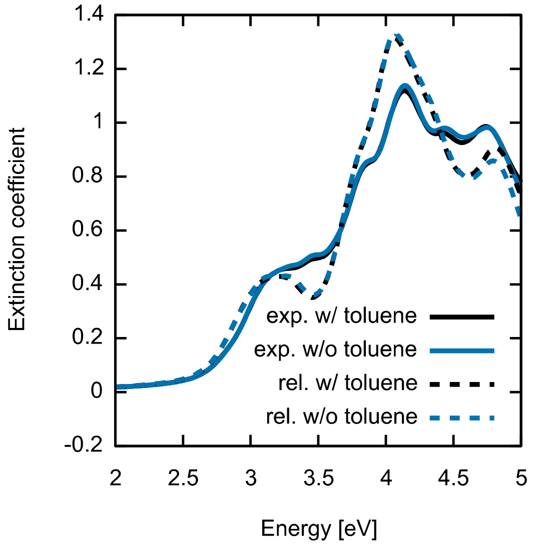

Figure S17. Extinction coefficient of the [$(NpSi)_4S_6$] molecular crystals calculated within the IPA at the DFT equilibrium geometry (dotted line) and at the experimentally determined atomic positions (solid line). Black and blue curves refer to supercell with and without toluene solvent.

WILEY-VCH

10 References for the Supporting Information

[1] B. M. Moore, S. M. Ramirez, G. R. Yandek, T. S. Haddad, J. M. Mabry, *J. Organomet. Chem.* **2011**, *696*, 2676–2680.

[2] T. S. Haddad, B. D. Viers, S. H. Phillips, *J. Inorg. Organomet. Polym.* **2001**, *11*, 155–164.

[3] N. W. Rosemann, J. P. Eußner, E. Dornsiepen, S. Chatterjee, S. Dehnen, *J. Am. Chem. Soc.* **2016**, *138*, 16224–16227

[4] P. Braunstein, H. Lehner, D. Matt, *Inorg. Synth.* **1990**, *27*, 218–221.

[5] G. Brauer, *Handbuch der Präparativen Anorganischen Chemie;* Ferdinand Enke Verlag, Stuttgart, **1975**.

[6] a) G. M. Sheldrick, *Acta Crystallogr, Sect. A* **2015**, *71*, 3–8; b) G. M. Sheldrick, *Acta Crystallogr, Sect. C* **2015**, *71*, 3–8; c) O. V. Dolomanov, L. J. Bourhis, R. J. Gildea, J. A. K. Howard, H. Puschmann, *J. Appl. Crystallogr.* **2009**, *42*, 339–341.

[7] Gaussian 09, Revision A.02, M. J. Frisch, G. W. Trucks, H. B. Schlegel, G. E. Scuseria, M. A. Robb, J. R. Cheeseman, G. Scalmani, V. Barone, G. A. Petersson, H. Nakatsuji, et al., Gaussian, Inc., Wallingford CT, **2016**.

[8] a) S. Grimme, J. Antony, S. Ehrlich, H. Krieg, *The Journal of Chemical Physics* **2010**, *132*, 154104; b) S. Grimme, S. Ehrlich, L. Goerigk, *J. Comput. Chem.* **2011**, *32*, 1456–1465; c) A. D. Becke, *Phys. Rev. A* **1988**, *38*, 3098–3100; d) J. P. Perdew, W. Yue, *Phys. Rev. B* **1986**, *33*, 8800–8802.

[9] R. A. Kendall, T. H. Dunning, R. J. Harrison, *The Journal of Chemical Physics* **1992**, *96*, 6796–6806.

[10] B. Metz, H. Stoll, M. Dolg, *The Journal of Chemical Physics* **2000**, *113*, 2563–2569.

[11] P. Pracht, F. Bohle, S. Grimme, *Phys. Chem. Chem. Phys.* **2020**, *22*, 7169–7192.

[12] a) S. Grimme, C. Bannwarth, P. Shushkov, *Journal of chemical theory and computation* **2017**, *13*, 1989–2009; b) C. Bannwarth, S. Ehlert, S. Grimme, *Journal of chemical theory and computation* **2019**, *15*, 1652–1671.

[13] I. Boldog, A. B. Lysenko, E. B. Rusanov, A. N. Chernega, K. V. Domasevitch, *Acta crystallographica. Section C, Crystal structure communications* **2009**, *65*, o248-o252.

[14] a) G. Kresse, J. Furthmüller, *Computational Materials Science* **1996**, *6*, 15–50; b) G. Kresse, J. Furthmüller, *Phys. Rev. B* **1996**, *54*, 11169–11186.

[15] P. E. Blöchl, *Phys. Rev. B* **1994**, *50*, 17953–17979.

[16] J. P. Perdew, K. Burke, M. Ernzerhof, *Physical review letters* **1996**, *77*, 3865–3868.

[17] E. Dornsiepen, F. Dobener, S. Chatterjee, S. Dehnen, *Angew. Chem.* **2019**, *131*, 17197–17202; *Angew. Chem. Int. Ed.* **2019**, *58*, 17041–17046.

[18] M. Gajdoš, K. Hummer, G. Kresse, J. Furthmüller, F. Bechstedt, *Phys. Rev. B* **2006**, *73*, 045112.

[19] A. Riefer, S. Sanna, A. Schindlmayr, W. G. Schmidt, *Phys. Rev. B* **2013**, *87*, 195208.

[20] C. Attaccalite, M. Grüning, *Phys. Rev. B* **2013**, *88*, 235113.

[21] A.L. Spek, *Acta Crystallogr., Sect. C: Struct. Chem.* **2015**, *71*, 9-18.

WILEY-VCH
